# Fast estimation of time-varying infectious disease transmission rates

**DOI:** 10.1371/journal.pcbi.1008124

**Published:** 2020-09-21

**Authors:** Mikael Jagan, Michelle S. deJonge, Olga Krylova, David J. D. Earn

**Affiliations:** 1 Department of Mathematics & Statistics, McMaster University, Hamilton, Ontario, Canada; 2 M.G. DeGroote Institute for Infectious Disease Research, McMaster University, Hamilton, Ontario, Canada; York University, CANADA

## Abstract

Compartmental epidemic models have been used extensively to study the historical spread of infectious diseases and to inform strategies for future control. A critical parameter of any such model is the transmission rate. Temporal variation in the transmission rate has a profound influence on disease spread. For this reason, estimation of time-varying transmission rates is an important step in identifying mechanisms that underlie patterns in observed disease incidence and mortality. Here, we present and test fast methods for reconstructing transmission rates from time series of reported incidence. Using simulated data, we quantify the sensitivity of these methods to parameters of the data-generating process and to mis-specification of input parameters by the user. We show that sensitivity to the user’s estimate of the initial number of susceptible individuals—considered to be a major limitation of similar methods—can be eliminated by an efficient, “peak-to-peak” iterative technique, which we propose. The method of transmission rate estimation that we advocate is extremely fast, for even the longest infectious disease time series that exist. It can be used independently or as a fast way to obtain better starting conditions for computationally expensive methods, such as iterated filtering and generalized profiling.

## Author summary

Many pathogens cause recurrent epidemics. Patterns of recurrence are strongly affected by seasonality of the transmission rate, which can arise from seasonal changes in weather and host population behaviour (*e.g*., aggregation of children in schools). To understand and predict recurrent epidemic patterns, it is essential to reconstruct the time-varying transmission rate, which is never observed directly. Existing transmission rate estimation methods tend to fall into one of two categories: accurate but too slow to apply to long time series of reported incidence, or fast but inaccurate unless the number of individuals initially susceptible to infection is known precisely. Here, we introduce and compare fast methods inspired by the algorithm that Fine and Clarkson pioneered in the early 1980s. The method that we suggest accurately reconstructs seasonally varying transmission rates, even with crude information about the initial size of the susceptible population.

## 1 Introduction

The transmission rate of an infectious disease is a salient quantity in the study of epidemics. Changes in the transmission rate over time greatly influence the spread of infection [1, 2]. Quantifying how it changes over time can elucidate factors governing disease spread (*e.g*., weather [3], contact patterns [4]), inform epidemic forecasts, and suggest strategies for epidemic control [5].

In practice, we do not observe transmission directly. Instead, we observe the number of cases of infection (disease incidence) or number of deaths from infection (disease mortality) reported over time, and must reconstruct time-varying transmission rates from these data [6–13]. Utilizing historical mortality records, it is possible to identify patterns in transmission dating far back in time. Most notably, the London Bills of Mortality and the Registrar General’s Weekly Returns enable investigation of transmission patterns continuously from the mid-17th century to the present, for a number of infectious diseases including cholera [14] and smallpox [15].

A mechanistic understanding of long infectious disease time series—three centuries of weekly data in the case of smallpox [15]—requires methods of transmission rate estimation that are both accurate and fast, and therefore tractable for long time scales. Simulation-based methods of transmission rate estimation from reported incidence or mortality have been developed using the susceptible-infected-removed (SIR) model for infectious disease dynamics [16]. Markov chain Monte Carlo (MCMC [17, 18]) and sequential Monte Carlo (as in iterated filtering [8, 19, 20]) methods are statistically rigorous, but not tractable for long time scales owing to high computational cost. Generalized profiling [21, 22], which combines trajectory and gradient matching, is faster, but still too slow for convenient exploration of time series spanning hundreds of years. (Several CPU hours were required to apply generalized profiling to just 26 years of weekly data [22].)

In comparison, Finkenstädt and Grenfell’s popular “time series SIR” (tSIR) method [7, 23] is extremely fast, using a simple discretization of a continuous-time SIR model to reduce transmission rate estimation to a local regression problem. However, the tSIR method assumes that the duration of infection is equal to the time step, that natural death of susceptible individuals can be ignored, and that cumulative incidence approximates cumulative births. The latter two assumptions are reasonable for pre-vaccination measles, when most susceptible individuals were eventually infected [6]. However, in many contexts (*e.g*., with pathogens less transmissible than measles), susceptible mortality over time scales of interest and the difference between incidence and births are non-negligible.

In their unpublished PhD and MSc theses, Krylova (Ch. 4 in [24]) and deJonge [25] separately modified a fast discretization method originally proposed by Fine and Clarkson [6]. Krylova’s approach has been employed to estimate the amplitude of seasonal variation in measles transmission in 20th century New York City [9]. Unlike the tSIR method and unlike Fine and Clarkson, Krylova’s and deJonge’s methods do not place constraints on the infectious period or ignore susceptible mortality.

Here, we present a new algorithm based on deJonge’s method and compare its performance to the methods of Fine and Clarkson and Krylova. Our main investigative approach is to apply each method to simulated reported incidence data with known underlying transmission rate, so that error in transmission rate estimates can be quantified exactly.

Our analysis of the methods reveals a shared sensitivity to process and observation error. We mitigate this issue by introducing a smoothing step. The methods are additionally sensitive to error in the user’s estimate of the initial number of susceptible individuals, which is rarely known with any precision. We propose a fast, iterative technique for estimating this parameter from time series of incidence, births, and natural mortality, eliminating a long-standing barrier to the use of fast methods of transmission rate reconstruction.

## 2 Methods

In §§2.1 and 2.2 below, we present three fast methods for estimating time-varying transmission rates, based on a mechanistic model of disease spread. In §§2.3–2.7, we outline our systematic analysis of the sensitivity of the methods to parameters of the data-generating process and to error in the user-specified values of input parameters. Finally, in §2.8, we introduce peak-to-peak iteration (PTPI), a technique for estimating the initial number of susceptible individuals. Essential notation is summarized in Table 1.

**Table 1:** Notation. Unless otherwise stated, simulations of reported incidence time series use the reference values listed here. If a symbol is to be interpreted differently in relation to disease incidence and disease mortality data, then the correct definition is indicated by (I) and (M), respectively.

Symbol	Name	Definition	Ref. val.	Unit	Notes
*t*_*k*_	*k*th observation time	Time of the *k*th observation in time series data, for *k* = 0, …, *n*.	*t*_0_ + *k*Δ*t*	years	
*t*_0_	Transient period	Duration of the transient in system (1) that is ignored in simulations of reported incidence, before observations are recorded.	2000	years	System (1) is numerically integrated between *t* = 0 years and *t* = *t*_0_, and observed starting at *t* = *t*_0_. This is done so that simulations reflect dynamics near the attractor of system (1).
Δ*t*	Observation interval	Time between successive observations in time series data.	1	weeks	Disease mortality is reported weekly in the London Bills of Mortality.
*n*	Time series length	Time between the initial and final observations in time series data, in units Δ*t*, given by (*t_n_* − *t*_0_)/Δ*t*.	1042	—	If Δ*t* = 1 week, then 1042Δ*t* = ⌊20 × 365/7⌋Δ*t* ≃ 20 years.
[·]_Δ*t*_	Nearest *k*Δ*t* rounding	For time lengths *t*, $[t{]}_{\Delta t}=[\frac{t}{\Delta t}]\Delta t$, where [⋅] denotes nearest integer rounding.	—	—	
〈⋅〉	Long-term averaging	For functions *x*(*t*), $\langle x\rangle ={\text{lim}}_{t\to \infty }\frac{1}{t}\int_{0}^{t}x\left(s\right)\;\text{d}s$. For sequences *x*_*k*_, $\langle x\rangle ={\text{lim}}_{n\to \infty }\frac{1}{n}\sum_{k=0}^{n}x_{k}$.	—	—	
(*S*(*t*), *I*(*t*), *R*(*t*))	State	Number of (susceptible, infected, removed) individuals in the population at time *t*.	—	—	“Removed” individuals have either recovered from the disease and gained permanent immunity or died from the disease.
*N*(*t*)	Population size	*S*(*t*) + *I*(*t*) + *R*(*t*).	—	—	
*B*(*t*)	Births	Number of births that occur during the time interval [*t* − Δ*t*, *t*).	—	—	
*Q*(*t*)	Cumulative incidence	Number of susceptibles who become infected during the time interval [*t*_0_, *t*).	—	—	
*Z*(*t*)	Incidence	Number of susceptibles who become infected during the time interval [*t* − Δ*t*, *t*).	*Q*(*t*) − *Q*(*t* − Δ*t*)	—	
*C*(*t*)	Reported disease (I) incidence or (M) mortality	Number of (I) infections or (M) disease-induced deaths reported during the time interval [*t* − Δ*t*, *t*).	Eq (31)	—	*C* is an abbreviation of “cases”, which are reported as infections or as deaths.
(*S*_0_, *I*_0_, *R*_0_)	Initial state (*t* = *t*_0_)	(*S*(*t*_0_), *I*(*t*_0_), *R*(*t*_0_)).	(S*, I*, R*)	—	(S*, I*, R*) denotes the state of system (1) after numerical integration between *t* = 0 years and *t* = *t*_0_ with seasonally forced transmission rate *β*(*t*) (Eq (27)), constant vital rates *ν*_c_ and *μ*_c_, and initial state (*t* = 0 years) $\left(\hat{S},\hat{I},\hat{R}\right)$ (Eq (32); see below).
*N*_0_	Initial population size (*t* = *t*_0_)	*N*(*t*_0_).	S* + I* + R*	—	
$\left(\hat{S},\hat{I},\hat{R}\right)$	Endemic equilibrium	Endemic equilibrium of system (1) with constant transmission rate (*β* ≡ 〈*β*〉) and constant vital rates (*ν* ≡ *μ* ≡ *μ*_c_).	Eq (32)	—	
${\hat{N}}_{0}$	Initial population size (*t* = 0 years)	*N*(0).	10^6^	—	
*x*_*k*_	Estimation input/output	Within an estimation algorithm (Boxes 1–3), the supplied or estimated value of *x*(*t*_*k*_) (*x* = *C*, *B*, *μ*, *Z*, *S*, *I*, *β*).	—	varies	
*ν*(*t*)	Birth rate	Number of births per unit time relative to ${\hat{N}}_{0}$, at time *t*.	*ν*_c_	year^−1^	In simulations of reported incidence, *ν*(*t*) is modeled as a constant *ν*_c_. In general, estimation of *β*(*t*) from data does not require the underlying *ν*(*t*) to be constant.
*ν*_c_	Birth rate (constant)	See *ν*(*t*) above.	0.04	year^−1^	
*μ*(*t*)	Natural mortality rate	Number of natural deaths per unit time *per capita*, at time *t*.	*μ*_c_	year^−1^	In simulations of reported incidence, *μ*(*t*) is modeled as a constant *μ*_c_. In general, estimation of *β*(*t*) from data does not require the underlying *μ*(*t*) to be constant.
*μ*_c_	Natural mortality rate (constant)	See *μ*(*t*) above.	0.04	year^−1^	
*t*_gen_	Mean generation interval	Mean time between onset of infection (in infector) and subsequent transmission of infection (by infector) [26, 27].	13	days	The reference value is the sum of the observed mean latent and infectious periods, which for measles are 8 days and 5 days, respectively [16].
*γ*	Removal rate	Number of removals (recoveries or deaths from disease) per unit time per infected.	1/*t*_gen_	day^−1^	
*p*_rep_	Case reporting probability	(I) Probability that an infection is reported, or (M) the case fatality ratio times the probability that a death from disease is reported.	0.25 or 1	—	If we simulate data with under-reporting, then we use *p*_rep_ = 0.25 as a reference value. Otherwise, we set *p*_rep_ = 1.
*t*_rep_	Mean case reporting delay	Mean time between infection and reporting of (I) infection or (M) disease-induced death.	2 or 0	weeks	If we simulate data with reporting delays, then we use *t*_rep_ = 2 weeks as a reference value. Otherwise, we set *t*_rep_ = 0 weeks.
*β*(*t*)	Transmission rate	Number of infections per unit time per susceptible per infected, at time *t*.	Eq (27)	year^−1^	In simulations of reported incidence, *β*(*t*) is modeled by the seasonal forcing function defined in Eq (27). In general, estimation of *β*(*t*) from data does not require it vary seasonally or even periodically.
〈*β*〉	Mean transmission rate	Continuous-time average of the seasonally forced *β*(*t*), equal to ${\text{lim}}_{t\to \infty }\frac{1}{t}\int_{0}^{t}\beta \left(s\right)\;\text{d}s$.	β*	year^−1^	β* ≈ 5.6 × 10^−4^year^−1^ is the value of 〈*β*〉 that satisfies Eq (2) with $R_{0}=20$, *ν*_c_ = *μ*_c_ = 0.04 year^−1^, *t*_gen_ = *γ*^−1^ = 13 days, and ${\hat{N}}_{0}=10^{6}$.
$R_{0}$	Basic reproduction number	Number of individuals that a typical infected person is expected to infect in an otherwise completely susceptible population.	Eq (2)	—	For measles in the 20th century, $R_{0}\approx 20$ [16].
*α*	Seasonal amplitude	Amplitude of the seasonally forced *β*(*t*) relative to 〈*β*〉.	0.08	—	For measles, *α* ≈ 0.08 [2]. We require *α* ∈ [0, 1] to ensure that the seasonal forcing function defined in Eq (27) is non-negative.
*ϕ*(*t*; *ϵ*)	Environmental noise (realized)	Phase shift in the seasonally forced *β*(*t*), at time *t*.	*Normal*(0, *ϵ*^2^)	—	*ϕ* is a realization of a continuous-time stochastic process defined by a set {Φ(*t*; *ϵ*)} of independent and *Normal*(0, *ϵ*^2^)-distributed random variables.
*ϵ*	Standard deviation of environmental noise	See *ϕ*(*t*; *ϵ*) above.	0.5	—	
*q*	Loess smoothing parameter	Rough number of nearest neighbours weighted in local regression (*i.e*., when fitting loess curves to time series), determining the degree of smoothing.	—	—	See §2.2.6 for an exact definition.

### 2.1 Model of disease transmission

We assume that the principal mechanisms of disease spread in the focal population are captured by the SIR model [16], formulated with time-varying rates of birth, death, and transmission. Expressing the model as a system of ordinary differential equations, we write

$$\frac{\text{d}S}{\text{d}t}=\nu \left(t\right){\hat{N}}_{0}-\beta \left(t\right)SI-\mu \left(t\right)S\;,$$ (1a)

$$\frac{\text{d}I}{\text{d}t}=\beta (t)SI-\gamma I-\mu (t)I\;,$$ (1b)

$$\frac{\text{d}R}{\text{d}t}=\gamma I-\mu (t)R\;,$$ (1c)

where *S*, *I*, and *R* are the numbers of individuals who are susceptible, infected, and removed, respectively; *N* = *S* + *I* + *R* is the population size; and ${\hat{N}}_{0}=N\left(0\right)$ is the population size at an initial time, defined to be 0 years for simplicity. (We reserve the notation *N*_0_ for *N*(*t*_0_), where *t*_0_ > 0 years is the length of a transient; see Table 1.)

The time-varying parameters are

*ν*(*t*) birth rate, the number of births per unit time relative to ${\hat{N}}_{0}$;
*μ*(*t*) natural mortality rate, the number of natural deaths per unit time *per capita* (*i.e*., relative to *N*); and
*β*(*t*) transmission rate, the number of infections per unit time per susceptible per infected.

The constant parameter *γ* is the rate of removal from the infected compartment (due to recovery or death from disease) per infected individual.

In Eq (1a) and Eq (1b), we use mass action incidence *β*(*t*)*SI* rather than standard incidence *β*(*t*)*SI*/*N*. Mass action incidence is essential for reproducing transitions in epidemic patterns resulting from changes in the birth rate [2, 28]. In Eq (1a), we write the net birth rate as $\nu \left(t\right){\hat{N}}_{0}$ rather than *ν*(*t*). This formulation is for convenience: the factor of ${\hat{N}}_{0}$ does not affect dynamics, but ensures that *ν*(*t*) and *μ*(*t*) have the same scale.

The SIR model (1) assumes that the focal population is homogeneously mixed and subject to the mass action principle, which states that incidence is proportional to the product of the densities of susceptibles and infecteds [16]. The model further assumes that the latent period (time from infection to onset of infectiousness) can be ignored and that the infectious period (time from onset of infectiousness to recovery or death from disease) is exponentially distributed [29]. The distributions of the latent and infectious periods affect disease dynamics [28, 30, 31], but Krylova and Earn [28] showed that the effect on long-term dynamical structure is typically small when the mean generation interval is fixed (see Fig 11 in [28]). For this reason, we assign the mean generation interval implied by the SIR model (1) (*t*_gen_ = *γ*^−1^) the value of the sum of the observed mean latent and infectious periods. This sum is the true mean generation interval if the latent and infectious periods are both exponentially distributed, and is a good estimate of the true mean generation interval more generally [28].

Transmissibility of infection is typically measured by the basic reproduction number $R_{0}$, defined as the number of individuals that a typical infected person is expected to infect in an otherwise completely susceptible population [16]. If the birth and death rates are constant (*ν* ≡ *ν*_*c*_ and *μ* ≡ *μ*_c_), and if the transmission rate has a well-defined average 〈*β*〉 [32], then the basic reproduction number for the SIR model (1) can be written as [28]

$$\begin{array}{c} R_{0}=\frac{\nu_{\text{c}}{\hat{N}}_{0}}{\mu_{\text{c}}}\cdot \frac{\left\langle \beta \right\rangle }{\gamma +\mu_{\text{c}}}\;. \end{array}$$ (2)

### 2.2 Estimating *β*(*t*) from time series data

Here, we examine three fast methods for estimating time-varying transmission rates *β*(*t*). The methods take as input (i) time series of reported disease incidence or disease mortality, (ii) time series of births and natural mortality, and (iii) values for input parameters, such as the mean generation interval *t*_gen_. By assumption, the time series data are available at discrete, equally spaced time points

$$\begin{array}{c} t_{k}=t_{0}+k\Delta t\;,\;k=0,\ldots ,n\;, \end{array}$$ (3)

where Δ*t* is the observation interval. The methods return as output a time series estimate of *β*(*t*), denoted by ${\left\{\left(t_{k},\beta_{k}\right)\right\}}_{k=0}^{n}$ or simply *β*_*k*_, which can be averaged (§2.2.5) or smoothed (§2.2.6) to distill temporal patterns of interest.

Missing data must be imputed: the three methods are recursive, so they break down as soon as they encounter a missing value. Imputation can be accomplished most simply via linear interpolation between available data. More sophisticated techniques accounting for uncertainty in missing values are described in [33].

#### 2.2.1 FC method

We review the method first described by Fine and Clarkson [6], referred to here as the “FC method”. Let *S*(*t*) and *I*(*t*) be the number of susceptibles and infecteds in the population at time *t*. *S* decreases when susceptibles become infected or die and increases when susceptibles are born. Let *Z*(*t*) and *B*(*t*) be the number of infections and births, respectively, that occur during the time interval [*t* − Δ*t*, *t*). Assuming that natural mortality was negligible, Fine and Clarkson reconstructed *S* from *Z* and *B* with the recursion

$$\begin{array}{c} S(t+\Delta t)\approx S(t)+B(t+\Delta t)-Z(t+\Delta t)\;. \end{array}$$ (4)

Fine and Clarkson further assumed that the observation interval Δ*t* was equal to the mean generation interval *t*_gen_, so that prevalence could be approximated by incidence. That is,

$$\begin{array}{c} I(t)\approx Z(t) \end{array}$$ (5)

for all *t*. They derived an expression for *Z*(*t* + Δ*t*) via the mass action principle

$$\begin{array}{c} Z(t+\Delta t)\approx \beta (t)S(t)I(t)\Delta t\;. \end{array}$$ (6)

Rearranging Eq (6), they obtained an estimate of *β*(*t*), given by

$$\begin{array}{c} \beta \left(t\right)\approx \frac{Z(t+\Delta t)}{S(t)I(t)\Delta t}\;. \end{array}$$ (7)

Fine and Clarkson applied Eqs (4), (5), and (7) to estimate *S*(*t*_*k*_), *I*(*t*_*k*_), and *β*(*t*_*k*_) (for *k* = 0, …, *n*), after specifying (i) the initial number of susceptibles *S*_0_ = *S*(*t*_0_), and (ii) values of *Z*(*t*_*k*_) and *B*(*t*_*k*_) from incidence and birth data, respectively.

A limitation of the FC method is the constraint requiring Δ*t* = *t*_gen_. For some diseases, this is a minor issue, because incidence and birth data can be aggregated so that the time between successive aggregates is approximately equal to *t*_gen_. For example, the mean generation interval of measles is approximately two weeks, so Fine and Clarkson [6] aggregated pairs of weekly observations. A second, more serious limitation is the assumption, implicit in Eqs (4) and (5), that natural mortality is negligible. We discuss this issue in §3.1.

#### 2.2.2 S method

Krylova (Ch. 4 in [24]) generalized the FC method in order to eliminate the constraint requiring Δ*t* = *t*_gen_ and account for natural mortality. Her approach is based on the SEIR model, which distinguishes “exposed” individuals in the latent stage of infection from infectious individuals. Here, we adapt Krylova’s approach to the SIR model (1) and refer to our approach as the “S method.”

We define *S*, *I*, *Z*, and *B* as in the FC method. Let *μ*(*t*) be the *per capita* natural mortality rate at time *t*, and let *Q*(*t*) be the total number of infections occurring between the initial observation time *t*_0_ and current time *t* (*i.e*., cumulative incidence). The observation interval Δ*t* is no longer constrained to be equal to the mean generation interval *t*_gen_.

We reconstruct *S* recursively by discretizing Eq (1a):

$$\begin{array}{c} S(t+\Delta t)\approx S(t)+B(t+\Delta t)-Z(t+\Delta t)-\mu (t)S(t)\Delta t\;. \end{array}$$ (8)

Eq (8) is the result of applying the forward Euler method for numerical integration to Eq (1a), and replacing the incidence and birth terms with *Z*(*t* + Δ*t*) and *B*(*t* + Δ*t*), respectively. Eq (8) is identical to Eq (4) of the FC method, except that it includes a natural mortality term.

In order to estimate *β*(*t*), we note that, by definition, d*Q*/d*t* is the rate at which individuals enter the infected compartment. From Eq (1b), this is

$$\begin{array}{c} \frac{\text{d}Q}{\text{d}t}=\beta \left(t\right)S\left(t\right)I\left(t\right)\;. \end{array}$$ (9)

If the mean generation interval *t*_gen_ is short enough that *I* and *μ* are roughly constant between times *t* and *t* + *t*_gen_, then d*I*/d*t* ≈ 0 in that interval, and using Eq (1b) we can write

$$\begin{array}{c} \beta \left(t\right)S\left(t\right)I\left(t\right)\approx (\gamma +\mu (t))I\left(t\right)\approx (\gamma +\mu (t+t_{\text{gen}}))I\left(t+t_{\text{gen}}\right)\;. \end{array}$$ (10)

In this case, d*Q*/d*t* is also (approximately) the rate at which individuals leave the infected compartment, *t*_gen_ time after infection:

$$\begin{array}{c} \frac{\text{d}Q}{\text{d}t}\approx (\gamma +\mu (t+t_{\text{gen}}))I\left(t+t_{\text{gen}}\right)\;. \end{array}$$ (11)

Note that Eq (11) is also valid if the generation interval is narrowly distributed around its mean *t*_gen_ (even if *t*_gen_ is long).

Discretizing Eqs (9) and (11) using forward Euler, we obtain two approximations of *Z*(*t* + Δ*t*):

$$\begin{array}{c} Z\left(t+\Delta t\right)=Q\left(t+\Delta t\right)-Q\left(t\right)\approx \{\begin{array}{cc} \beta (t)S(t)I(t)\Delta t & \text{from}\;\text{Eq}\;(9),\\ (\gamma +\mu (t+t_{\text{gen}}))I\left(t+t_{\text{gen}}\right)\Delta t & \text{from}\;\text{Eq}\;(11). \end{array} \end{array}$$ (12)

Rearranging Eq (12) yields an estimate of *β*(*t*), given by

$$\begin{array}{c} \beta \left(t\right)\approx \frac{Z(t+\Delta t)}{S(t)I(t)\Delta t}\;, \end{array}$$ (13)

and an estimate of *I*(*t*), given by

$$\begin{array}{c} I\left(t\right)\approx \frac{Z(t+\Delta t-t_{\text{gen}})}{(\gamma +\mu (t))\Delta t}\;. \end{array}$$ (14)

Since data are available only at the observation times *t*_*k*_ (Eq (3)), the value of *Z*(*t* + Δ*t* − *t*_gen_) in Eq (14) will be observed only if *t*_gen_ is an integer multiple of Δ*t*. In general, *t*_gen_ is not divisible by Δ*t*. Therefore, in practice, we replace *t*_gen_ in *Z*(*t* + Δ*t* − *t*_gen_) with the nearest integer multiple of Δ*t*, denoted here by [*t*_gen_]_Δ*t*_:

$$\begin{array}{c} I\left(t\right)\approx \frac{Z(t+\Delta t-t_{\text{gen}})}{(\gamma +\mu (t))\Delta t}\;. \end{array}$$ (15)

Thus, the S method is defined by Eq (13), coupled with Eqs (8) and (15) for the reconstruction of *S* and *I*. The S method requires users to specify (i) input parameters *S*_0_ = *S*(*t*_0_) and *t*_gen_ = *γ*^−1^, and (ii) values of *Z*(*t*_*k*_), *B*(*t*_*k*_), and *μ*(*t*_*k*_) from incidence, birth, and natural mortality data, respectively.

The FC method is a special case of the S method, obtained by setting Δ*t* = *t*_gen_ and *μ*(*t*) ≡ 0.

#### 2.2.3 SI method

DeJonge [25] improved Krylova’s method (Ch. 4 in [24]) by reconstructing *I* directly from Eq (1b) instead of relying on the approximation in Eq (11). Here, we improve deJonge’s discretization, which employs the forward Euler method, by instead combining forward and backward Euler. One way to do this is to use the trapezoidal method: whereas forward and backward Euler take *f* ′(*t*)Δ*t* and *f* ′(*t* + Δ*t*)Δ*t*, respectively, to approximate integrals $\int_{t}^{t+\Delta t}f^{\prime }\left(\tau \right)\;\text{d}\tau$, the trapezoidal method takes the average $\frac{1}{2}\left[f^{\prime }\left(t\right)+f^{\prime }\left(t+\Delta t\right)\right]\Delta t$, which is less prone to error. Our discretization, which we call the “SI method”, is consistently more accurate than deJonge’s and others (see §S9 of S1 Text for a comparison of nine possible algorithms). Numerically integrating Eq (1a) and Eq (1b) using the trapezoidal method, and replacing the incidence and birth terms with *Z*(*t* + Δ*t*) and *B*(*t* + Δ*t*), respectively, we obtain

$$\begin{array}{c} S\left(t+\Delta t\right)\approx \frac{[1-\frac{1}{2}\mu \left(t\right)\Delta t]S\left(t\right)+B\left(t+\Delta t\right)-Z\left(t+\Delta t\right)}{1+\frac{1}{2}\mu \left(t+\Delta t\right)\Delta t} \end{array}$$ (16)

and

$$\begin{array}{c} I\left(t+\Delta t\right)\approx \frac{[1-\frac{1}{2}(\gamma +\mu (t))\Delta t]I\left(t\right)+Z\left(t+\Delta t\right)}{1+\frac{1}{2}(\gamma +\mu (t+\Delta t))\Delta t}\;. \end{array}$$ (17)

Eq (17) eliminates an important problem with Eq (15) of the S method, which estimates *I*(*t*) ≈ 0 if *Z*(*t* + Δ*t* − [*t*_gen_]_Δ*t*_) = 0, leading to division by zero in Eq (13).

Discretizing Eq (9) using forward and backward Euler, we obtain two approximations of *Z*(*t* + Δ*t*):

$$\begin{array}{c} Z\left(t+\Delta t\right)=Q\left(t+\Delta t\right)-Q\left(t\right)\approx \{\begin{array}{cc} \beta (t)S(t)I(t)\Delta t & \text{from}\;\text{forward}\;\text{Euler},\\ \beta (t+\Delta t)S(t+\Delta t)I(t+\Delta t)\Delta t & \text{from}\;\text{backward}\;\text{Euler}. \end{array} \end{array}$$ (18)

Rearranging Eq (18) yields two estimates of *β*(*t*),

$$\begin{array}{c} \beta \left(t\right)\approx \{\begin{array}{cc} \frac{Z(t+\Delta t)}{S(t)I(t)\Delta t} & \text{from}\;\text{forward}\;\text{Euler},\\ \frac{Z(t)}{S(t)I(t)\Delta t} & \text{from}\;\text{backward}\;\text{Euler}, \end{array} \end{array}$$ (19)

whose average supplies a more accurate estimate (see §S9 of S1 Text), given by

$$\begin{array}{c} \beta \left(t\right)\approx \frac{Z(t)+Z(t+\Delta t)}{2S(t)I(t)\Delta t}\;. \end{array}$$ (20)

Thus, the SI method is defined by Eq (20), coupled with Eqs (16) and (17) for the reconstruction of *S* and *I*. Compared to the S method, the SI method, in principle, requires one additional input parameter, namely the initial number of infecteds *I*_0_ = *I*(*t*_0_). In §3.6, we show that, in practice, this additional information is not necessary.

#### 2.2.4 Estimating true incidence from reported incidence

Let *C*(*t*) be the number of infections reported during the time interval [*t* − Δ*t*, *t*). We estimate true incidence *Z* from reported incidence *C* via

$$\begin{array}{c} Z\left(t\right)\approx \frac{1}{p_{\text{rep}}}C\left(t+[t_{\text{rep}}{]}_{\Delta t}\right)\;, \end{array}$$ (21)

where *p*_rep_ is the probability that an infection is reported and [*t*_rep_]_Δ*t*_ is the mean time between infection and reporting, rounded to the nearest integer multiple of the observation interval Δ*t*.

Eq (21) has the limitation that multiplying by $p_{\text{rep}}^{-1}$ does not correct for under-reporting if, by chance, *C*(*t* + [*t*_rep_]_Δ*t*_) = 0. In this situation, not only is the result *Z*(*t*) ≈ 0 incorrect, but we divide by zero in the FC and S methods when we substitute Eqs (5) and (15) in Eqs (7) and (13), respectively. If *C*(*t* + [*t*_rep_]_Δ*t*_) = *C*(*t* + [*t*_rep_]_Δ*t*_ + Δ*t*) = 0, then the SI method also suffers: Eq (20) gives *β*(*t*) ≈ 0. To circumvent these issues, we replace zeros in reported incidence time series by linearly interpolating between nonzero values prior to estimating true incidence using Eq (21). We do not replace leading and trailing zeros.

If what we observe is deaths from disease, rather than infections, then we have the complication that only a fraction of infections end in death. In this situation, we can still use Eq (21) to estimate *Z*, provided we interpret (i) *C* as reported disease mortality, (ii) *p*_rep_ as the case fatality ratio times the probability that a death from disease is reported, and (iii) *t*_rep_ as the mean time between infection and reporting of disease-induced death.

A more sophisticated method of inferring true incidence from reported data is described in [34].

#### 2.2.5 Averaging raw estimates of *β*(*t*)

Given fixed time series data and input parameters, the FC, S, and SI methods return estimates of *β*(*t*) that are entirely determined (not random). In the absence of additional data observed from the same population, it is difficult to assign confidence to the output.

However, if an estimate $\tilde{\beta }\left(t\right)$ is approximately periodic (with apparent period *T*) and contains *m* complete cycles, and if we assume *β*(*t*) is truly periodic, then we can view $\tilde{\beta }\left(t\right)$ as containing a sample of *m* estimates of the true cycle, with some variance, and use its mean as an estimator instead of any one of the *m* cycles. For such an estimate $\tilde{\beta }\left(t\right)$ defined on the interval [*t*_0_, *t*_0_ + *mT*), the mean and variance are given by

$$\bar{x}\left(t\right)=\frac{1}{m}\sum_{i=0}^{m-1}\tilde{\beta }\left(t+iT\right)\;,\;t\in \left[t_{0},t_{0}+T\right)\;,$$ (22a)

$$s^{2}\left(t\right)=\frac{1}{m-1}\sum_{i=0}^{m-1}{\left[\tilde{\beta }\left(t+iT\right)-\bar{x}\left(t\right)\right]}^{2}\;,\;t\in \left[t_{0},t_{0}+T\right)\;.$$ (22b)

In §3.3, we apply the S and SI methods to simulated data to estimate an underlying, seasonally forced *β*(*t*) (Eq (27)), which has a period of 1 year. We linearly interpolate the raw time series estimate *β*_*k*_ and compute the average 1-year cycle in the interpolant *β*_int_(*t*) using Eq (22a). Comparing this average to the true 1-year cycle, we are able to assess bias in the two methods.

Note that $\bar{x}\left(t\right)$ and *s*^2^(*t*) can be used to obtain a formal, likelihood-based measure of confidence in estimates $\tilde{\beta }\left(t\right)$ (see §2.3.4 in [35]).

#### 2.2.6 Smoothing raw estimates of *β*(*t*)

Process and observation error introduce random fluctuations in reported incidence on top of longer-term (*e.g*., seasonal) variation. In §3.2, we show that noise in reported incidence is spuriously amplified in *β*_*k*_, the raw time series estimate of *β*(*t*).

To distill temporal patterns of interest from the noise, we fit a smooth loess (short for local regression; see Ch. 8.1 in [36]) curve *β*_loess_(*t*; *q*) to the points ${\left\{\left(t_{k},\beta_{k}\right)\right\}}_{k=0}^{n}$ and use *β*_loess_(*t*; *q*) as our final estimate of *β*(*t*). Here, *q* ∈ {5, …, *n* + 1} is an integer-valued parameter controlling the degree of smoothing. At times *t* ∈ [*t*_0_, *t*_*n*_], the fitted value *β*_loess_(*t*; *q*) is obtained as follows:

1. Order the distances *d*_*k*_ = |*t*_*k*_ − *t*| of the time points *t*_*k*_ (Eq (3)) from *t*, letting $d_{k_{i}}$ denote the *i*th smallest distance (for *i* = 1, …, *n* + 1).
2. Fit a quadratic polynomial *p*_2_(*t*) to the points ${\left\{\left(t_{k},\beta_{k}\right)\right\}}_{k=0}^{n}$. This is done by weighted least squares using tricube weights
  $$\begin{array}{c} w_{k}=\{\begin{array}{cc} {\left(1-{\left(\frac{d_{k}}{d_{k_{q}}}\right)}^{3}\right)}^{3} & \text{if}\;0\leq d_{k}<d_{k_{q}}\;,\\ 0 & \text{if}\;d_{k}\geq d_{k_{q}}\;. \end{array} \end{array}$$ (23)
  Hence only time points *t*_*k*_ nearer to *t* than the *q*th nearest time point are weighted in the fit.
3. Define *β*_loess_(*t*; *q*) = *p*_2_(*t*).

Typically, smoother fits are obtained with greater *q* [36, 37].

The optimal value of *q* for a given time series *β*_*k*_, denoted by *q*_opt_, is that which minimizes error in *β*_loess_(*t*; *q*) relative to *β*(*t*). In §3.4, we estimate *β*(*t*) from simulated data, smooth *β*_*k*_ using each value of *q* on a grid, and use our knowledge of *β*(*t*) to determine *q*_opt_. We show that it is possible for smoothing to eliminate much of the error in *β*_*k*_ attributable to process and observation error. Thus, in §2.2.7, we explicitly define the FC, S, and SI methods with loess smoothing as a final step.

In practice, *β*(*t*) is not known, so we cannot determine *q*_opt_. In this case, *q*_opt_ can be estimated using statistical methods, such as time series cross-validation [38]. However, reasonable results can be obtained much more quickly by inspecting *β*_loess_(*t*; *q*) directly and increasing *q* from 4 until a desirable degree of smoothing is achieved (*e.g*., until noise on the time scale of weeks is visibly reduced, and patterns on the time scale of months are easier to discern).

#### 2.2.7 Summary

In Boxes 1–3 below, we summarize the three methods derived in §§2.2.1–2.2.6 for estimating time-varying transmission rates *β*(*t*) from time series data with observation times *t*_*k*_ (Eq (3)). We use the notation *x*_*k*_ to refer to the value supplied or computed for *x*(*t*_*k*_) within the estimation algorithms (*x* = *C*, *B*, *μ*, *Z*, *S*, *I*, *β*).

**Box 1. FC method (Fine & Clarkson 1982 [6])**

$$Z_{k}\;\leftarrow \;\frac{1}{p_{\text{rep}}}C_{k+r},\;\text{where}\;r=\frac{{\left[t_{\text{rep}}\right]}_{\Delta t}}{\Delta t},$$ (24a)

$$S_{k}\;\leftarrow \;S_{k-1}+B_{k}-Z_{k}\;,$$ (24b)

$$I_{k}\;\leftarrow \;Z_{k}\;,$$ (24c)

$$\beta_{k}\;\leftarrow \;\frac{Z_{k+1}}{S_{k}I_{k}\Delta t}\;,$$ (24d)

where Δ*t* is assumed to be roughly equal to *t*_gen_, and natural mortality is assumed to be negligible. Users must specify:

- a time series ${\left\{\left(t_{k},C_{k}\right)\right\}}_{k=0}^{n}$ of reported incidence or reported disease mortality, with zeros replaced via linear interpolation between nonzero values;
- a time series ${\left\{\left(t_{k},B_{k}\right)\right\}}_{k=0}^{n}$ of births;
- input parameters *S*_0_, *t*_gen_, *p*_rep_, and *t*_rep_.

**Box 2. S method (adapted from Krylova 2011 [24])**

$$Z_{k}\;\leftarrow \;\frac{1}{p_{\text{rep}}}C_{k+r}\;,\;\text{where}\;r=\frac{{\left[t_{\text{rep}}\right]}_{\Delta t}}{\Delta t},$$ (25a)

$$S_{k}\;\leftarrow \;S_{k-1}+B_{k}-Z_{k}-\mu_{k-1}S_{k-1}\Delta t\;,$$ (25b)

$$I_{k}\;\leftarrow \;\frac{Z_{k+1-g}}{(\gamma +\mu_{k})\Delta t}\;,\;\text{where}\;g=\frac{{\left[t_{\text{gen}}\right]}_{\Delta t}}{\Delta t}\;,$$ (25c)

$$\beta_{k}\;\leftarrow \;\frac{Z_{k+1}}{S_{k}I_{k}\Delta t}\;,$$ (25d)

$$\beta_{\text{loess}}\left(t;q\right)\;\leftarrow \;\text{loess}\;\text{curve}\;\text{fit}\;\text{to}\;\text{time}\;\text{series}\;{\left\{\left(t_{k},\beta_{k}\right)\right\}}_{k=0}^{n}\;.$$ (25e)

Users must specify:

- a time series ${\left\{\left(t_{k},C_{k}\right)\right\}}_{k=0}^{n}$ of reported incidence or reported disease mortality, with zeros replaced via linear interpolation between nonzero values;
- a time series ${\left\{\left(t_{k},B_{k}\right)\right\}}_{k=0}^{n}$ of births;
- a time series ${\left\{\left(t_{k},\mu_{k}\right)\right\}}_{k=0}^{n}$ of the *per capita* natural mortality rate;
- input parameters *S*_0_, *t*_gen_ = *γ*^−1^, *p*_rep_, and *t*_rep_;
- loess smoothing parameter *q*.

**Box 3. SI method (adapted from deJonge 2014 [25])**

$$Z_{k}\;\leftarrow \;\frac{1}{p_{\text{rep}}}C_{k+r}\;,\;\text{where}\;r=\frac{{\left[t_{\text{rep}}\right]}_{\Delta t}}{\Delta t}\;,$$ (26a)

$$S_{k}\;\leftarrow \;\frac{[1-\frac{1}{2}\mu_{k-1}\Delta t]S_{k-1}+B_{k}-Z_{k}}{1+\frac{1}{2}\mu_{k}\Delta t}\;,$$ (26b)

$$I_{k}\;\leftarrow \;\frac{[1-\frac{1}{2}(\gamma +\mu_{k-1})\Delta t]I_{k-1}+Z_{k}}{1+\frac{1}{2}(\gamma +\mu_{k})\Delta t}\;,$$ (26c)

$$\beta_{k}\;\leftarrow \;\frac{Z_{k}+Z_{k+1}}{2S_{k}I_{k}\Delta t}\;,$$ (26d)

$$\beta_{\text{loess}}\left(t;q\right)\;\leftarrow \;\text{loess}\;\text{curve}\;\text{fit}\;\text{to}\;\text{time}\;\text{series}\;{\left\{\left(t_{k},\beta_{k}\right)\right\}}_{k=0}^{n}\;.$$ (26e)

Users must specify:

- a time series ${\left\{\left(t_{k},C_{k}\right)\right\}}_{k=0}^{n}$ of reported incidence or reported disease mortality, with zeros replaced via linear interpolation between nonzero values;
- a time series ${\left\{\left(t_{k},B_{k}\right)\right\}}_{k=0}^{n}$ of births;
- a time series ${\left\{\left(t_{k},\mu_{k}\right)\right\}}_{k=0}^{n}$ of the *per capita* natural mortality rate;
- input parameters *S*_0_, *I*_0_, *t*_gen_ = *γ*^−1^, *p*_rep_, and *t*_rep_;
- loess smoothing parameter *q*.

In Box 4, we provide instructions for input specification based on our analysis of the methods.

**Box 4. Instructions for input specification**

- *β*_*k*_ is sensitive to mis-specification of *S*_0_, but not *I*_0_ (*cf*. §3.6.1). If the user’s estimate of *S*_0_ is uncertain, and if the incidence time series *Z*_*k*_ is roughly periodic, then a more accurate estimate of *S*_0_ may be obtained via peak-to-peak iteration (PTPI; *cf*. §3.7).
- If *S*_*k*_ is negative for any *k*, then it is likely that the case reporting probability *p*_rep_ was underestimated or that births were systematically under-reported by *B*_*k*_. This can be resolved by correcting the estimate of *p*_rep_ or correcting *B*_*k*_, then restarting the algorithm. Users should apply close to the minimal correction necessary to prevent negative *S*_*k*_.
- *q* must be tuned to the *β*_*k*_ time series. An estimate of *q*_opt_ can be obtained using statistical methods, such as time series cross-validation [38]. However, *q* can be tuned quickly through visual inspection of *β*_loess_(*t*; *q*): one can increase *q* from 5 until a desirable degree of smoothing is achieved (*e.g*., until noise on the time scale of weeks is visibly reduced, and patterns on the time scale of months are easier to discern).

### 2.3 Simulating reported incidence data

In order to compare the performance of the FC, S, and SI methods in estimating *β*(*t*), we apply the methods to simulated reported incidence data with known underlying *β*(*t*). Here, we outline our methods for simulating these data using the SIR model (1).

#### 2.3.1 Seasonal forcing of *β*(*t*) with environmental stochasticity

We reproduce seasonal fluctuation in the transmission rate by modeling *β*(*t*) in Eq (1) as a sinusoidal forcing function with period equal to one year:

$$\begin{array}{c} \beta \left(t\right)=\left\langle \beta \right\rangle (1+\alpha \;\text{cos}\;(\frac{2\pi t}{\text{1}\;\text{year}}))\;. \end{array}$$ (27)

Here, *α* ∈ [0, 1] is the amplitude of seasonal forcing relative to the mean 〈*β*〉. We introduce stochastic fluctuation by adding a randomly generated phase shift:

$$\begin{array}{c} \beta_{\phi }\left(t\right)=\left\langle \beta \right\rangle (1+\alpha \;\text{cos}\;(\frac{2\pi t}{\text{1}\;\text{year}}+\phi \left(t;\epsilon \right)))\;. \end{array}$$ (28)

*ϕ* is a realization of a continuous-time stochastic process consisting of independent, Normal(0, *ϵ*^2^)-distributed random variables. It models ***environmental stochasticity*** leading to random noise in the transmission rate. Modeling environmental stochasticity with a random phase shift rather than additive noise conveniently avoids negative *β*_*ϕ*_(*t*): *β*_*ϕ*_(*t*) oscillates between 〈*β*〉(1 − *α*) and 〈*β*〉(1 + *α*) regardless of the distribution of the noise. In practice, we take the values of *ϕ* at times *t*_*k*_ (Eq (3)) and linearly interpolate to obtain values in between. This helps to make simulations of Eqs (1) and (9) with adaptive time steps (*cf*. §2.3.2) reproducible.

#### 2.3.2 Generating incidence time series with demographic stochasticity

We supplement Eq (1) with Eq (9), so that trajectories of the resulting system record changes in cumulative incidence *Q*. In this system, we employ the noisy transmission rate *β*_*ϕ*_(*t*) (Eq (28)) and constant vital rates *ν*_c_ and *μ*_c_. We then either (i) numerically integrate the differential equations to approximate their solution, or (ii) treat the system more realistically as an event-driven, continuous-time Markov process (with event rates specified by terms in the differential equations) and use the adaptive tau-leaping algorithm for stochastic simulation [39, 40]. The latter approach accounts for ***demographic stochasticity*** in disease dynamics. We prevent disease fadeout in simulations with demographic stochasticity by setting the rates of infected recovery and death to zero whenever *I* = 1.

In both methods of simulation, we record the state of the system at times *t*_*k*_ (Eq (3)), choosing initial state

$$\begin{array}{c} (\begin{array}{c} S(t_{0})\\ I(t_{0})\\ R(t_{0})\\ Q(t_{0}) \end{array})=(\begin{array}{c} S_{0}\\ I_{0}\\ R_{0}\\ 0 \end{array})\;, \end{array}$$ (29)

where *S*_0_ + *I*_0_ + *R*_0_ = *N*_0_ = *N*(*t*_0_). Finally, we derive incidence *Z* from *Q* via first differences:

$$\begin{array}{c} Z(t)=Q(t)-Q(t-\Delta t)\;. \end{array}$$ (30)

#### 2.3.3 Introducing observation error

Observation error due to under-reporting (*p*_rep_ < 1) and reporting delays (*t*_rep_ > 0 weeks) creates discrepancies between true incidence *Z* and reported incidence *C*. We introduce random observation error to simulated incidence time series with delayed binomial sampling:

$$\begin{array}{c} C\left(t+[t_{\text{rep}}{]}_{\Delta t}\right)∼\text{Binomial}(Z\left(t\right),p_{\text{rep}})\;. \end{array}$$ (31)

For simulations without observation error, we set *p*_rep_ < 1 and *t*_rep_ > 0 weeks.

#### 2.3.4 Parametrization

The simulation method outlined in §§2.3.1–2.3.3 is parametrized by

$$\begin{array}{cc} \text{disease}\;\text{parameters}\; & \left\langle \beta \right\rangle ,\;\alpha ,\;\epsilon ,\;t_{\text{gen}}\;;\\ \text{population}\;\text{parameters}\; & {\hat{N}}_{0}=N\left(0\right),\;S_{0}=S\left(t_{0}\right),\;I_{0}=I\left(t_{0}\right),\;\nu_{\text{c}},\;\mu_{\text{c}},\;;\;\text{and}\\ \text{reporting}\;\text{parameters}\; & p_{\text{rep}},\;t_{\text{rep}},\;t_{0},\;\Delta t,\;n\;. \end{array}$$

For most simulations, we assign parameters the reference values listed in Table 1. We consider different values when we investigate the sensitivity of *β*(*t*) estimates to data-generating parameters (*cf*. §2.6.1).

We bypass transient dynamics by choosing *t*_0_ = 2000 years and numerically integrating system (1) between 0 years and *t*_0_ in order to obtain a point (S*, I*, R*) very near the attractor. For this step, we exclude environmental noise, defining *β*(*t*) as in Eq (27), and take the initial state to be the endemic equilibrium of the unforced system (system (1) with *β* ≡ 〈*β*〉 and *ν* ≡ *μ* ≡ *μ*_c_):

$$\begin{array}{c} (\begin{array}{c} S(0)\\ I(0)\\ R(0) \end{array})=(\begin{array}{c} \hat{S}\\ \hat{I}\\ \hat{R} \end{array})=(\begin{array}{c} \frac{{\hat{N}}_{0}}{R_{0}}\\ {\hat{N}}_{0}(1-\frac{1}{R_{0}})(\frac{\mu_{\text{c}}}{\gamma +\mu_{\text{c}}})\\ {\hat{N}}_{0}(1-\frac{1}{R_{0}})(\frac{\gamma }{\gamma +\mu_{\text{c}}}) \end{array})\;. \end{array}$$ (32)

### 2.4 Creating mock birth and natural mortality time series

In addition to reported incidence data, the FC, S, and SI methods require time series of births and the *per capita* natural mortality rate. For simplicity, we create mock time series by (i) choosing constant vital rates $\nu_{\text{c}}^{\prime }$ and $\mu_{\text{c}}^{\prime }$, then (ii) setting $B_{k}=\nu_{\text{c}}^{\prime }{\hat{N}}_{0}\Delta t$ and $\mu_{k}=\mu_{\text{c}}^{\prime }$ for all *k*. Note that $\nu_{\text{c}}^{\prime }{\hat{N}}_{0}\Delta t$ is the result of integrating the net birth rate in the SIR model (1), given by $\nu \left(t\right){\hat{N}}_{0}$, between successive observation times using $\nu \equiv \nu_{\text{c}}^{\prime }$.

We specify $\nu_{\text{c}}^{\prime }=\nu_{\text{c}}$ and $\mu_{\text{c}}^{\prime }=\mu_{\text{c}}$, where *ν*_c_ and *μ*_c_ are the data-generating vital rates (*cf*. §2.3.4), except when we investigate the sensitivity of *β*(*t*) estimates to incorrect vital data (*cf*. §2.6.2). For example, to model under-reporting of births, we simply set $\nu_{\text{c}}^{\prime }<\nu_{\text{c}}$.

### 2.5 Measuring *β*(*t*) estimation error

When we simulate reported incidence data, the underlying transmission rate *β*(*t*) is defined beforehand via Eq (27) and known for all *t*. We use this knowledge to quantify the error in estimates of *β*(*t*) obtained from the data. Specifically, given an estimate $\tilde{\beta }\left(t\right)$ defined at time points *t*_*k*_ (Eq (3)), we compute the relative root mean square error (RRMSE), defined as

$$\begin{array}{c} \text{RRMSE}\left(\beta ,\tilde{\beta }\right)≔\sqrt{\frac{1}{n+1}\sum_{k=0}^{n}{\left(\frac{\beta \left(t_{k}\right)-\tilde{\beta }\left(t_{k}\right)}{\bar{\beta }}\right)}^{2}}\;, \end{array}$$ (33)

where

$$\begin{array}{c} \bar{\beta }≔\frac{1}{n+1}\sum_{k=0}^{n}\beta \left(t_{k}\right)\;. \end{array}$$ (34)

Note that by “underlying transmission rate” we mean the transmission rate *excluding* environmental noise. Although we simulate data using the noisy *β*_*ϕ*_(*t*), defined in Eq (28), our aim is to reconstruct the noiseless *β*(*t*), defined in Eq (27).

### 2.6 Sensitivity analysis

Error in *β*(*t*) estimation from reported incidence data depends on how the data were generated. The number of cases reported over time is influenced by features of the disease (*e.g*., the natural history of infection), population (*e.g*., contact patterns), and case reporting (*e.g*., the frequency and accuracy of reports). In our simulations of reported incidence, there are 14 ***data-generating parameters*** (*cf*. §2.3.4), whose values are summarized in the vector

$$\begin{array}{c} \theta =(\left\langle \beta \right\rangle ,\;\alpha ,\;\epsilon ,\;{\hat{N}}_{0},\;S_{0},\;I_{0},\;\nu_{\text{c}},\;\mu_{\text{c}},\;t_{\text{gen}},\;p_{\text{rep}},\;t_{\text{rep}},\;t_{0},\;\Delta t,\;n)\;. \end{array}$$ (35)

Estimation error also depends on how accurately certain data-generating parameters are specified by users of the FC, S, and SI methods. The initial observation time *t*_0_, observation interval Δ*t*, and time series length *n* are always known exactly. Other parameters (〈*β*〉, *α*, *ϵ*, ${\hat{N}}_{0}$, *ν*_c_, and *μ*_c_) influence our simulations of reported incidence, but in practice are not parameters of the FC, S, and SI methods. In practice, users are required to specify only *S*_0_, *t*_gen_, *p*_rep_, *t*_rep_, and (with the SI method) *I*_0_. However, when we test the methods here, we do specify vital rates *ν*_c_ and *μ*_c_ in order to create mock (constant) birth and natural mortality time series (*cf*. §2.4). The specified values of these 7 ***input parameters*** are summarized in the vector

$$\begin{array}{c} \theta^{\prime }=\left(S_{0}^{\prime },\;I_{0}^{\prime },\;\nu_{\text{c}}^{\prime },\;\mu_{\text{c}}^{\prime },\;t_{\text{gen}}^{\prime },\;p_{\text{rep}}^{\prime },\;t_{\text{rep}}^{\prime }\right)\;. \end{array}$$ (36)

First, we investigate the sensitivity of the methods to the data-generating parameter values ***θ***. Then, we examine their sensitivity to error in the user’s specification ***θ***′ of the input parameters. Here, we describe our analysis using the notation $\tilde{\beta }\left(t;\theta ,\theta^{\prime }\right)$ to refer to transmission rate estimates constructed with user input ***θ***′, from data generated by parameter values ***θ***.

#### 2.6.1 Sensitivity to data-generating parameters

In §3.5, we consider the ideal situation in which the input ***θ***′ corresponds exactly to the data-generating ***θ***. In this case, how sensitive is error in $\tilde{\beta }\left(t;\theta ,\theta^{\prime }\right)$ to ***θ***? For example, is *β*(*t*) estimated more accurately for diseases with longer mean generation interval *t*_gen_, *etc*.? To answer these questions, we perform the following steps on a grid of data-generating parameter values ***θ***:

1. Simulate 1000 reported incidence time series using ***θ***.
2. Create corresponding mock (constant) birth and natural mortality time series (*cf*. §2.4), specifying $\nu_{\text{c}}^{\prime }=\nu_{\text{c}}$ and $\mu_{\text{c}}^{\prime }=\mu_{\text{c}}$ in the input ***θ***′.
3. Estimate *β*(*t*) from the simulated data, specifying $S_{0}^{\prime }=S_{0}$, $I_{0}^{\prime }=I_{0}$, $t_{\text{gen}}^{\prime }=t_{\text{gen}}$, $p_{\text{rep}}^{\prime }=p_{\text{rep}}$, and $t_{\text{rep}}^{\prime }=t_{\text{rep}}$ in the input ***θ***′.
4. Compute the median RRMSE in the estimates $\tilde{\beta }\left(t_{k};\theta ,\theta^{\prime }\right)$ (1000 estimates corresponding to 1000 simulations).

We repeat this analysis 6 times, corresponding to 2 methods of *β*(*t*) estimation (S or SI) and 3 methods of data simulation:

- *without* demographic stochasticity and *without* observation error (fixing *p*_rep_ = 1, *t*_rep_ = 0 weeks),
- *with* demographic stochasticity but *without* observation error (fixing *p*_rep_ = 1, *t*_rep_ = 0 weeks), or
- *with* demographic stochasticity and *with* observation error (fixing *p*_rep_ = 0.25 unless sensitivity to *p*_rep_ is considered, *t*_rep_ = 2 weeks).

Environmental stochasticity (*ϵ* = 0.5) is included in all simulations.

#### 2.6.2 Sensitivity to mis-specification of input parameters

In §3.6, we fix the data-generating ***θ*** and consider the more realistic situation in which components of the input ***θ***′ differ from the corresponding components of ***θ*** by a potentially large factor. In this case, how sensitive is error in $\tilde{\beta }\left(t;\theta ,\theta^{\prime }\right)$ to error in ***θ***′? For example, how important is having an accurate estimate of *t*_gen_, *etc*.? To answer these questions, we perform the following steps:

1. Simulate 1000 reported incidence time series using fixed data-generating parameter values ***θ***. (We assign the reference values listed in Table 1.)
2. For each point on a grid of input parameter values ***θ***′:
  - Create mock (constant) birth and natural mortality time series, taking $\nu_{\text{c}}^{\prime }$ and $\mu_{\text{c}}^{\prime }$ from the input ***θ***′.
  - Estimate *β*(*t*) from the simulated data, taking $S_{0}^{\prime }$, $I_{0}^{\prime }$, $t_{\text{gen}}^{\prime }$, $p_{\text{rep}}^{\prime }$, and $t_{\text{rep}}^{\prime }$ from the input ***θ***′.
  - Compute the median RRMSE in the estimates $\tilde{\beta }\left(t_{k};\theta ,\theta^{\prime }\right)$ (1000 estimates corresponding to 1000 simulations).

We repeat this analysis 6 times, as outlined at the end of §2.6.1.

### 2.7 Asymptotic analysis

Here, we examine analytically the propagation of input error to the output of the SI method. (Similar expressions for propagated errors are obtained by analyzing the S method.) Our analysis here supports numerical results presented in §3.6 concerning the sensitivity of *β*(*t*) estimation error to mis-specification of input parameters.

#### 2.7.1 Explicit solutions of the (*S*_*k*_, *I*_*k*_) difference equations

The SI method uses Eq (26a) to Eq (26c) to recursively reconstruct *S*(*t*) and *I*(*t*) from time series of reported incidence, births, and natural mortality. After substitution of Eq (26a), Eq (26b) and Eq (26c) can be written as

$$\begin{array}{cc} S_{k+1} & =\frac{1-\frac{1}{2}\mu_{k}\Delta t}{1+\frac{1}{2}\mu_{k+1}\Delta t}S_{k}+\frac{B_{k+1}-\frac{1}{p_{\text{rep}}}C_{k+1+r}}{1+\frac{1}{2}\mu_{k+1}\Delta t}\;,\;k=0,1,\ldots \;, \end{array}$$ (37a)

$$I_{k+1}=\frac{1-\frac{1}{2}\left(\gamma +\mu_{k}\right)\Delta t}{1+\frac{1}{2}\left(\gamma +\mu_{k+1}\right)\Delta t}I_{k}+\frac{\frac{1}{p_{\text{rep}}}C_{k+1+r}}{1+\frac{1}{2}\left(\gamma +\mu_{k+1}\right)\Delta t}\;,\;k=0,1,\ldots \;,$$ (37b)

where *r* = [*t*_rep_]_Δ*t*_/Δ*t* is the mean case reporting delay in units of the observation interval, rounded to the nearest integer. Eq (37) are linear, first order difference equations, whose explicit solutions are obtained using standard algebraic techniques (see Eq 1.2.4 in [41]) and given by

$$\begin{array}{cc} S_{k} & =S_{0}\prod_{j=0}^{k-1}\frac{1-\frac{1}{2}\mu_{j}\Delta t}{1+\frac{1}{2}\mu_{j+1}\Delta t}+\sum_{i=0}^{k-1}\left(B_{i+1}-\frac{1}{p_{\text{rep}}}C_{i+1+r}\right)\prod_{j=i+1}^{k-1}\frac{1-\frac{1}{2}\mu_{j}\Delta t}{1+\frac{1}{2}\mu_{j+1}\Delta t}\;,\;k=0,1,\ldots \;, \end{array}$$ (38a)

$$\begin{array}{cc} I_{k} & =I_{0}\prod_{j=0}^{k-1}\frac{1-\frac{1}{2}\left(\gamma +\mu_{j}\right)\Delta t}{1+\frac{1}{2}\left(\gamma +\mu_{j+1}\right)\Delta t}+\sum_{i=0}^{k-1}\frac{1}{p_{\text{rep}}}C_{i+1+r}\prod_{j=i+1}^{k-1}\frac{1-\frac{1}{2}\left(\gamma +\mu_{j}\right)\Delta t}{1+\frac{1}{2}\left(\gamma +\mu_{j+1}\right)\Delta t}\;,\;k=0,1,\ldots \;, \end{array}$$ (38b)

with the conventions $\sum_{i=b}^{a}x_{i}=0$ and $\prod_{i=b}^{a}x_{i}=1$ if *a* < *b*. As we show in §2.7.2, explicit solutions of Eq (37) facilitate asymptotic analysis.

#### 2.7.2 Propagation of input error to (*S*_*k*_, *I*_*k*_)

We consider the special case in which the vital rates are constant and set $B_{k}=\nu_{\text{c}}{\hat{N}}_{0}\Delta t$ and *μ*_*k*_ = *μ*_c_ for all *k* (*cf*. §2.4). Then Eq (38) simplify to

$$\begin{array}{c} \begin{array}{cc} & S_{k}\left(S_{0},\nu_{\text{c}},\mu_{\text{c}},p_{\text{rep}}\right)\\ & =S_{0}{\left(\frac{1-\frac{1}{2}\mu_{\text{c}}\Delta t}{1+\frac{1}{2}\mu_{\text{c}}\Delta t}\right)}^{k}+\sum_{i=0}^{k-1}\frac{\nu_{\text{c}}{\hat{N}}_{0}\Delta t-\frac{1}{p_{\text{rep}}}C_{i+1+r}}{1+\frac{1}{2}\mu_{\text{c}}\Delta t}{\left(\frac{1-\frac{1}{2}\mu_{\text{c}}\Delta t}{1+\frac{1}{2}\mu_{\text{c}}\Delta t}\right)}^{k-1-i} \end{array} \end{array}$$ (39a)

$$\begin{array}{c} \begin{array}{cc} & I_{k}\left(I_{0},\mu_{\text{c}},t_{\text{gen}},p_{\text{rep}}\right)\\ & =I_{0}{\left(\frac{1-\frac{1}{2}\left(\gamma +\mu_{\text{c}}\right)\Delta t}{1+\frac{1}{2}\left(\gamma +\mu_{\text{c}}\right)\Delta t}\right)}^{k}+\sum_{i=0}^{k-1}\frac{\frac{1}{p_{\text{rep}}}C_{i+1+r}}{1+\frac{1}{2}\left(\gamma +\mu_{\text{c}}\right)\Delta t}{\left(\frac{1-\frac{1}{2}\left(\gamma +\mu_{\text{c}}\right)\Delta t}{1+\frac{1}{2}\left(\gamma +\mu_{\text{c}}\right)\Delta t}\right)}^{k-1-i} \end{array} \end{array}$$ (39b)

where we have made explicit the dependence of *S*_*k*_ and *I*_*k*_ on input parameters *S*_0_, *I*_0_, *ν*_c_, *μ*_c_, *t*_gen_ = *γ*^−1^, and *p*_rep_. Using Eq (39), we can derive exact expressions for the error propagated to *S*_*k*_ and *I*_*k*_ in the SI method as a result of assigning an incorrect value to an input parameter.

If the initial number of susceptibles is truly *S*_0_, but we specify $S_{0}^{\prime }=\omega S_{0}$, where *ω* > 0, then the error propagated to *S*_*k*_ is

$$\begin{array}{c} \begin{array}{cc} \text{Err}(S_{k},S_{0}\leftarrow \omega S_{0}) & =S_{k}\left(\omega S_{0},\nu_{\text{c}},\mu_{\text{c}},p_{\text{rep}}\right)-S_{k}\left(S_{0},\nu_{\text{c}},\mu_{\text{c}},p_{\text{rep}}\right)\\ & =\left(\omega -1\right)S_{0}{\left(\frac{1-\frac{1}{2}\mu_{\text{c}}\Delta t}{1+\frac{1}{2}\mu_{\text{c}}\Delta t}\right)}^{k}\\ & =\left(\omega -1\right)S_{0}{\left(\frac{1-\frac{\Delta t}{2t_{\text{life}}}}{1+\frac{\Delta t}{2t_{\text{life}}}}\right)}^{k}\;\overset{k\to \infty }{\to }0\;, \end{array} \end{array}$$ (40)

where $t_{\text{life}}=\mu_{\text{c}}^{-1}$ is the life expectancy in the population. Similarly, specifying $I_{0}^{\prime }=\omega I_{0}$ for *I*_0_ yields an error

$$\begin{array}{c} \begin{array}{cc} \text{Err}(I_{k},I_{0}\leftarrow \omega I_{0}) & =I_{k}\left(\omega I_{0},\mu_{\text{c}},t_{\text{gen}},p_{\text{rep}}\right)-I_{k}\left(I_{0},\mu_{\text{c}},t_{\text{gen}},p_{\text{rep}}\right)\\ & =\left(\omega -1\right)I_{0}{\left(\frac{1-\frac{1}{2}\left(\gamma +\mu_{\text{c}}\right)\Delta t}{1+\frac{1}{2}\left(\gamma +\mu_{\text{c}}\right)\Delta t}\right)}^{k}\\ & =\left(\omega -1\right)I_{0}{\left(\frac{1-\frac{\Delta t}{2t_{\text{inf}}}}{1+\frac{\Delta t}{2t_{\text{inf}}}}\right)}^{k}\;\overset{k\to \infty }{\to }\;0 \end{array} \end{array}$$ (41)

in *I*_*k*_, where *t*_inf_ = (*γ* + *μ*_c_)^−1^ is the mean time between infection and removal from the infected compartment, accounting for the possibility of natural death during infection. Eqs (40) and (41) show that the errors propagated to *S*_*k*_ and *I*_*k*_ vanish as *k* → ∞; we exploit this fact to improve susceptible reconstruction (*cf*. §2.8).

Mis-specifying *ν*_c_ by assigning a value $\nu_{\text{c}}^{\prime }=\omega \nu_{\text{c}}$ creates an error in *S*_*k*_ that increases in magnitude over time and converges to a limit:

$$\begin{array}{c} \begin{array}{cc} & \text{Err}(S_{k},\nu_{\text{c}}\leftarrow \omega \nu_{\text{c}})\\ & =S_{k}\left(S_{0},\omega \nu_{\text{c}},\mu_{\text{c}},p_{\text{rep}}\right)-S_{k}\left(S_{0},\nu_{\text{c}},\mu_{\text{c}},p_{\text{rep}}\right)\\ & =\sum_{i=0}^{k-1}\frac{\left(\omega -1\right)\nu_{\text{c}}{\hat{N}}_{0}\Delta t}{1+\frac{1}{2}\mu_{\text{c}}\Delta t}{\left(\frac{1-\frac{1}{2}\mu_{\text{c}}\Delta t}{1+\frac{1}{2}\mu_{\text{c}}\Delta t}\right)}^{k-1-i}\\ & =\frac{\left(\omega -1\right)\nu_{\text{c}}{\hat{N}}_{0}\Delta t}{1+\frac{1}{2}\mu_{\text{c}}\Delta t}\sum_{i=0}^{k-1}{\left(\frac{1-\frac{1}{2}\mu_{\text{c}}\Delta t}{1+\frac{1}{2}\mu_{\text{c}}\Delta t}\right)}^{i}\\ & =\frac{\left(\omega -1\right)\nu_{\text{c}}{\hat{N}}_{0}}{\mu_{\text{c}}}[1-{\left(\frac{1-\frac{1}{2}\mu_{\text{c}}\Delta t}{1+\frac{1}{2}\mu_{\text{c}}\Delta t}\right)}^{k}]\;\overset{k\to \infty }{\to }\;\left(\omega -1\right)\nu_{\text{c}}{\hat{N}}_{0}t_{\text{life}}\;. \end{array} \end{array}$$ (42)

Unlike Eq (42), the exact expression for Err(*S*_*k*_, *μ*_c_ ← *ωμ*_c_) is not readily simplified and is difficult to interpret:

$$\begin{array}{c} \begin{array}{cc} & \text{Err}(S_{k},\mu_{\text{c}}\leftarrow \omega \mu_{\text{c}})\\ & =S_{k}\left(S_{0},\nu_{\text{c}},\omega \mu_{\text{c}},p_{\text{rep}}\right)-S_{k}\left(S_{0},\nu_{\text{c}},\mu_{\text{c}},p_{\text{rep}}\right)\\ & =S_{0}{\left(\frac{1-\frac{1}{2}\omega \mu_{\text{c}}\Delta t}{1+\frac{1}{2}\omega \mu_{\text{c}}\Delta t}\right)}^{k}+\sum_{i=0}^{k-1}\frac{\nu_{\text{c}}{\hat{N}}_{0}\Delta t-\frac{1}{p_{\text{rep}}}C_{i+1+r}}{1+\frac{1}{2}\omega \mu_{c}\Delta t}{\left(\frac{1-\frac{1}{2}\omega \mu_{\text{c}}\Delta t}{1+\frac{1}{2}\omega \mu_{\text{c}}\Delta t}\right)}^{k-1-i}\\ & \;-S_{0}{\left(\frac{1-\frac{1}{2}\omega \mu_{\text{c}}\Delta t}{1+\frac{1}{2}\omega \mu_{\text{c}}\Delta t}\right)}^{k}-\sum_{i=0}^{k-1}\frac{\nu_{\text{c}}{\hat{N}}_{0}\Delta t-\frac{1}{p_{\text{rep}}}C_{i+1+r}}{1+\frac{1}{2}\mu_{c}\Delta t}{\left(\frac{1-\frac{1}{2}\mu_{\text{c}}\Delta t}{1+\frac{1}{2}\mu_{\text{c}}\Delta t}\right)}^{k-1-i}\;. \end{array} \end{array}$$ (43)

However, if *C*_*k*_ has a well-defined long-term average 〈*C*〉 (this will be true if, for instance, *C*_*k*_ is periodic), then Err(*S*_*k*_, *μ*_c_ ← *ωμ*_c_) has a well-defined long-term average 〈Err(*S*_*k*_, *μ*_c_ ← *ωμ*_c_)〉 with a simple form. Replacing *C*_*i*+1+*r*_ in Eq (43) with 〈*C*〉, simplifying the resulting expression, then taking the limit as *k* → ∞, we obtain

$$\begin{array}{c} \begin{array}{cc} & \left\langle \text{Err}(S_{k},\mu_{\text{c}}\leftarrow \omega \mu_{\text{c}})\right\rangle \\ & =\underset{k\to \infty }{\text{lim}}\{S_{0}{\left(\frac{1-\frac{1}{2}\omega \mu_{\text{c}}\Delta t}{1+\frac{1}{2}\omega \mu_{\text{c}}\Delta t}\right)}^{k}+\frac{\nu_{\text{c}}{\hat{N}}_{0}\Delta t-\frac{1}{p_{\text{rep}}}\left\langle C\right\rangle }{\omega \mu_{\text{c}}\Delta t}[1-{\left(\frac{1-\frac{1}{2}\omega \mu_{\text{c}}\Delta t}{1+\frac{1}{2}\omega \mu_{\text{c}}\Delta t}\right)}^{k}]\\ & \;-S_{0}{\left(\frac{1-\frac{1}{2}\mu_{\text{c}}\Delta t}{1+\frac{1}{2}\mu_{\text{c}}\Delta t}\right)}^{k}-\frac{\nu_{\text{c}}{\hat{N}}_{0}\Delta t-\frac{1}{p_{\text{rep}}}\left\langle C\right\rangle }{\mu_{\text{c}}\Delta t}[1-{\left(\frac{1-\frac{1}{2}\mu_{\text{c}}\Delta t}{1+\frac{1}{2}\mu_{\text{c}}\Delta t}\right)}^{k}]\}\\ & =(\frac{1}{\omega }-1)(\nu_{\text{c}}{\hat{N}}_{0}\Delta t-\frac{1}{p_{\text{rep}}}\left\langle C\right\rangle )\frac{t_{\text{life}}}{\Delta t}\;, \end{array} \end{array}$$ (44)

We can similarly show the following, still assuming that 〈*C*〉 is well-defined:

$$\langle \text{Err}(I_{k},\mu_{\text{c}}\leftarrow \omega \mu_{\text{c}})\rangle =\frac{\left\langle C\right\rangle \left(t_{\text{inf}}^{\prime }-t_{\text{inf}}\right)}{p_{\text{rep}}\Delta t}\;,\;t_{\text{inf}}^{\prime }={\left(\gamma +\omega \mu_{\text{c}}\right)}^{-1}\;,$$ (45)

$$\langle \text{Err}(I_{k},t_{\text{gen}}\leftarrow \omega t_{\text{gen}})\rangle =\frac{\left\langle C\right\rangle \left(t_{\text{inf}}^{\prime }-t_{\text{inf}}\right)}{p_{\text{rep}}\Delta t}\;,\;t_{\text{inf}}^{\prime }={\left(\frac{1}{\omega }\gamma +\mu_{\text{c}}\right)}^{-1}\;,$$ (46)

$$\langle \text{Err}(S_{k},p_{\text{rep}}\leftarrow \omega p_{\text{rep}})\rangle =(1-\frac{1}{\omega })\frac{\left\langle C\right\rangle t_{\text{life}}}{p_{\text{rep}}\Delta t}\;,$$ (47)

$$\langle \text{Err}(I_{k},p_{\text{rep}}\leftarrow \omega p_{\text{rep}})\rangle =(\frac{1}{\omega }-1)\frac{\left\langle C\right\rangle t_{\text{inf}}}{p_{\text{rep}}\Delta t}\;.$$ (48)

Here, $t_{\text{inf}}^{\prime }$ is the (incorrect) mean time spent infected that results when *ωμ*_c_ is incorrectly specified for *μ*_c_ (Eq (45)) or *ωt*_gen_ is incorrectly specified for *t*_gen_ (Eq (46)).

#### 2.7.3 Propagation of error in (*S*_*k*_, *I*_*k*_) to *β*_*k*_

Let *β*_*k*_(*Z*_*k*_, *Z*_*k*+1_, *S*_*k*_, *I*_*k*_) be the raw SI method estimate of *β*(*t*_*k*_), given by the right hand side of Eq (26d). Suppose that, due to propagated error (*cf*. §2.7.2), the arguments are incorrect by a factor, so that

$$\begin{array}{c} Z_{k}=\omega_{Z}Z\left(t_{k}\right)\;,\;Z_{k+1}=\omega_{Z}Z\left(t_{k+1}\right)\;,\;S_{k}=\omega_{S}S\left(t_{k}\right)\;,\;I_{k}=\omega_{I}I\left(t_{k}\right)\;, \end{array}$$ (49)

where *ω*_*Z*_, *ω*_*S*_, *ω*_*I*_ > 0. Then the computed *β*_*k*_ will have relative error

$$\begin{array}{c} \frac{\beta_{k}(Z_{k},Z_{k+1},S_{k},I_{k})-\beta_{k}(Z\left(t_{k}\right),Z\left(t_{k+1}\right),S\left(t_{k}\right),I\left(t_{k}\right))}{\beta_{k}(Z\left(t_{k}\right),Z\left(t_{k+1}\right),S\left(t_{k}\right),I\left(t_{k}\right))}=\frac{\omega_{Z}}{\omega_{S}\omega_{I}}-1\;. \end{array}$$ (50)

Hence severe underestimation of *S*_*k*_ or *I*_*k*_ (*ω*_*S*_ ≪ 1 or *ω*_*I*_ ≪ 1) causes the relative error in *β*_*k*_ to blow up.

### 2.8 Estimating *S*_0_ via peak-to-peak iteration

Reconstruction of susceptibles *S*(*t*) is a necessary step in the reconstruction of *β*(*t*) using the FC, S, and SI methods. In §3.6, we show that susceptible reconstruction requires accurate specification of the initial number of susceptibles *S*_0_ = *S*(*t*_0_). However, reliable estimates of *S*_0_ have, to this point, been difficult to obtain in practice.

We propose a technique for iteratively improving estimates of *S*_0_, requiring only incidence, birth, and natural mortality data at times *t*_*k*_ (Eq (3)). Crucially, our technique, which we call “peak-to-peak iteration” (PTPI), enables accurate susceptible reconstruction without direct observation of the susceptible population size at the initial time.

Our approach is motivated by application of the SI method to simulated data. When we incorrectly guessed the value of *S*_0_ and attempted to reconstruct *S*(*t*) via Eq (26b), the absolute error in the reconstruction ${\left\{\left(t_{k},S_{k}\right)\right\}}_{k=0}^{n}$ decreased monotonically over time (*k*). (Eq (40) shows that the error propagated from *S*_0_ to *S*_*k*_ vanishes as *k* → ∞.) Consequently, if the underlying dynamics are at least approximately periodic, and if *t*_0_ and *t*_*n*_ occur at the same phase of the cycle, then *S*_*n*_ is actually a better estimate of *S*_0_ than our initial guess. In this situation, instead of reconstructing *β*(*t*) directly, we can use *S*_*n*_ as an updated estimate of *S*_0_, and reconstruct *S*(*t*) more accurately. This procedure can be repeated any number of times, and, with simulated data, we observe rapid convergence to an accurate estimate of *S*_0_ (*cf*. §3.7).

The key point is that the reconstructed final state can be used as an improved estimate of the initial state only if the initial and final states occur at the same phase of the cycle. This will not be true unless the observation period (the time between the first and last observations in time series data) is an integer multiple of the period of the underlying dynamics. We can ensure this by choosing appropriate times at which to start and stop *S*(*t*) reconstruction. In noisy periodic data, the points in a cycle that are easiest to identify robustly are the peaks. Consequently, we ignore observations (i) prior to the time *t*_*a*_ of the first peak in the incidence time series and (ii) after the time *t*_*b*_ of the last peak that occurs near an integer multiple of the apparent period after the first peak. For the truncated time series, the iterations converge to an accurate estimate of *S*(*t*_*a*_) starting from an initial guess, and we recover the corresponding accurate estimate of *S*_0_ by solving Eq (26b) backwards in time, from *t*_*a*_ to *t*_0_:

$$\begin{array}{c} S_{k-1}\approx \frac{[1+\frac{1}{2}\mu_{k}\Delta t]S_{k}-B_{k}+Z_{k}}{1-\frac{1}{2}\mu_{k-1}\Delta t}\;. \end{array}$$ (51)

The complete PTPI algorithm, which consists of finding *t*_*a*_ and *t*_*b*_ (truncation step) and estimating *S*_0_ (iteration step), is outlined in Boxes 5 and 6 below. In §3.7, we assess the performance of PTPI by applying the technique to simulated data with known underlying *S*_0_, starting from an incorrect initial estimate of *S*_0_.

**Box 5. Peak-to-peak iteration: Truncation step**

**Goal**: Given a roughly periodic time series ${\left\{\left(t_{k},Z_{k}\right)\right\}}_{k=0}^{n}$ of incidence, we want to find the time *t*_*a*_ of the first peak and the time *t*_*b*_ of the last peak occurring at the same phase of the cycle. These times are necessary for the iteration step (Box 6).

**Algorithm**:

- Smooth the raw incidence time series *Z*_*k*_ by applying a (2*ℓ*_1_ + 1)-point central moving average, computed via
  $$\begin{array}{c} {\bar{Z}}_{k}=\frac{1}{2\ell_{1}+1}\sum_{i=-\ell_{1}}^{\ell_{1}}Z_{k+i}\;,\;k=\ell_{1},\ldots ,n-\ell_{1}\;. \end{array}$$ (52)
  Choose minimal *ℓ*_1_ large enough to remove spurious peaks in *Z*_*k*_ caused by noise, while retaining true peaks.
- Identify the period *T* of the smoothed incidence time series ${\left\{\left(t_{k},{\bar{Z}}_{k}\right)\right\}}_{k=\ell_{1}}^{n-\ell_{1}}$ from its power spectrum, and calculate the number of embedded cycles, given by $m=\lfloor \frac{t_{n-\ell_{1}}-t_{\ell_{1}}}{T}\rfloor$.
- Construct the set I indexing peaks in ${\left\{\left(t_{k},{\bar{Z}}_{k}\right)\right\}}_{k=\ell_{1}}^{n-\ell_{1}}$:
  $$\begin{array}{c} I=\{k\in \left\{\ell_{1}+\ell_{2},\ldots ,n-\ell_{1}-\ell_{2}\right\}\;:\;{\bar{Z}}_{k}>{\bar{Z}}_{k\pm i}\;\text{for}\;\text{all}\;i=1,\ldots ,\ell_{2}\}\;. \end{array}$$ (53)
  Choose minimal *ℓ*_2_ large enough to ensure that I indexes true peaks in ${\bar{Z}}_{k}$, but not spurious peaks caused by noise (any that remain after smoothing).
- Define $T=\{t_{k}:k\in I\}$, the set of times of peaks in ${\bar{Z}}_{k}$, and record the time of the first peak, given by $t_{a}=\min\left(T\right)$.
- For *i* = 0, …, *m*, define *τ*_*i*_ = *t*_*a*_+ *iT* and find the element of T nearest *τ*_*i*_, namely $\underset{\tau \in T}{\text{arg}\;\text{min}}|\tau_{i}-\tau |$. The resulting subset $T_{\text{phase}}$ should contain successive time points that are roughly one period apart, *i.e*., the corresponding peaks in ${\bar{Z}}_{k}$ should occur at the same phase of the cycle.
- Record the time of the last such peak, given by $t_{b}=\text{max}\left(T_{\text{phase}}\right)$.

**Box 6. Peak-to-peak iteration: Iteration step**

**Goal**: We want to produce an accurate estimate of the initial number of susceptibles *S*_0_ = *S*(*t*_0_), given

- a roughly periodic time series ${\left\{\left(t_{k},Z_{k}\right)\right\}}_{k=0}^{n}$ of incidence,
- a time series ${\left\{\left(t_{k},B_{k}\right)\right\}}_{k=0}^{n}$ of births,
- a time series ${\left\{\left(t_{k},\mu_{k}\right)\right\}}_{k=0}^{n}$ of the *per capita* natural mortality rate,
- times *t*_*a*_ and *t*_*b*_ as defined in the truncation step (Box 5), and
- an initial estimate of *S*_0_.

**Algorithm**:

- Define an initial estimate of *S*(*t*_*a*_). (We use the initial estimate of *S*_0_.)
- Reconstruct *S*(*t*) between times *t*_*a*_ and *t*_*b*_ using Eq (26b), starting with the current estimate of *S*(*t*_*a*_).
- Update the estimate of *S*(*t*_*a*_) with the estimate of *S*(*t*_*b*_) obtained in (ii).
- Repeat (ii) and (iii) until the sequence of estimates of *S*(*t*_*a*_) converges (to within a desirable tolerance).
- Reconstruct *S*(*t*) between times *t*_0_ and *t*_*a*_ using Eq (51), starting with the final estimate of *S*(*t*_*a*_) obtained in (iv). The reconstruction is performed backwards in time, from *t*_*a*_ to *t*_0_.
- Record the estimate of *S*_0_ = *S*(*t*_0_) computed in (v). This value can be passed back to Eq (26b), allowing for reconstruction of *S*(*t*) between times *t*_0_ and *t*_*n*_, as usual.

## 3 Results

In §3.1, we compare the performance of the FC, S, and SI methods in estimating *β*(*t*) from an idealized reported incidence time series. In §3.2, we show how process and observation error create spurious noise in estimates of *β*(*t*). In §§3.3 and 3.4, we examine averaging and smoothing as ways to distill temporal patterns of interest from noisy estimates of *β*(*t*). In §§3.5 and 3.6, we summarize our systematic analysis of the sensitivity of *β*(*t*) estimation error to data-generating parameters and to mis-specification of input parameters by the user. In §3.7, addressing apparent sensitivity to mis-specification of the initial number of susceptibles *S*_0_, we assess the performance of PTPI as a method of estimating *S*_0_. Finally, in §3.8, we report the run times of the S and SI methods and PTPI.

The results reported here are entirely reproducible using the annotated R code available in S1 File.

### 3.1 Example of *β*(*t*) estimation using the FC, S, and SI methods

**Fig 1:**
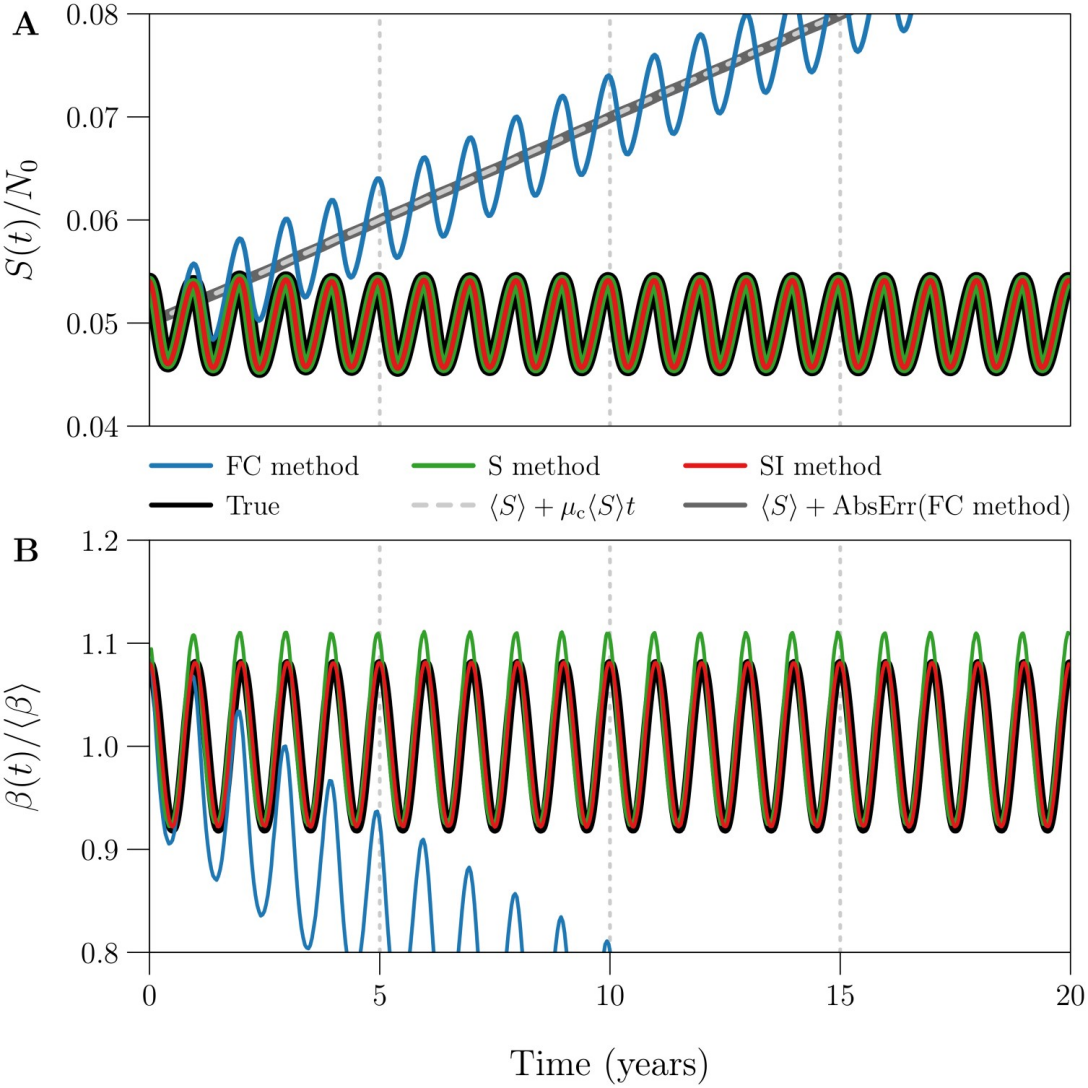
Example of *S*(*t*) and *β*(*t*) estimation using the FC, S, and SI methods. Plotted are the susceptible population size *S*(*t*) and seasonally forced transmission rate *β*(*t*) (Eq (27)) underlying 20 years of weekly reported incidence, together with time series estimates *S*_*k*_ and *β*_*k*_ obtained from the data by the FC [blue], S [green], and SI [red] methods. The reported incidence time series (Δ*t* = 1 week, *n* = ⌊20 × 365/7⌋ = 1042) was simulated without process or observation error (*ϵ* = 0, *p*_rep_ = 1), using reference values (Table 1) for all other data-generating parameters. The three estimation methods were applied without input error, *i.e*., all input parameters were assigned their true (data-generating) values. **[Panel A]** *S*(*t*) scaled by 1/*N*_0_, describing the number of susceptibles as a proportion of the initial population size. Grey lines show that the absolute error in the FC method estimate of *S*(*t*) increases linearly as *μ*_c_ 〈*S*〉*t*, where *μ*_c_ is the constant *per capita* natural mortality rate and 〈*S*〉 is the continuous-time average of *S*(*t*). **[Panel B]** *β*(*t*) scaled by 1/〈*β*〉, describing the transmission rate relative to its mean. RRMSE (Eq (33)) in the *β*_*k*_ time series generated by the (FC, S, SI) method is roughly (0.3355, 0.0240, 0.0021).

We applied the FC, S, and SI methods without input error to estimate *S*(*t*) and *β*(*t*) from an idealized reported incidence time series, simulated without process or observation error. The time series estimates *S*_*k*_ and *β*_*k*_ are shown in Fig 1. The S and SI methods estimated *S*(*t*) and *β*(*t*) accurately at every time point, whereas the FC method captured seasonality but failed otherwise. In the FC method, *S*_*k*_ neglects natural mortality (Eq (24b)), so it increases without bound while *β*_*k*_ decays to zero due to division by *S*_*k*_ (Eq (24d)).

Fig 1A confirms that the absolute error in the FC method estimate of *S*(*t*) increases linearly as *μ*_c_ 〈*S*〉*t*, where *μ*_c_ is the constant *per capita* natural mortality rate and 〈*S*〉 is the continuous-time average of *S*(*t*). In practice, the FC method fails whenever natural mortality in the underlying population is non-negligible. Since the S and SI methods address this limitation at effectively no computational cost, we do not present further analysis of the FC method.

In Fig 1B, the SI method estimate of *β*(*t*) was very accurate (RRMSE ≈ 0.2%), whereas the S method estimate peaked too early and too high (RRMSE ≈ 2.4%).

### 3.2 Effects of process and observation error

**Fig 2:**
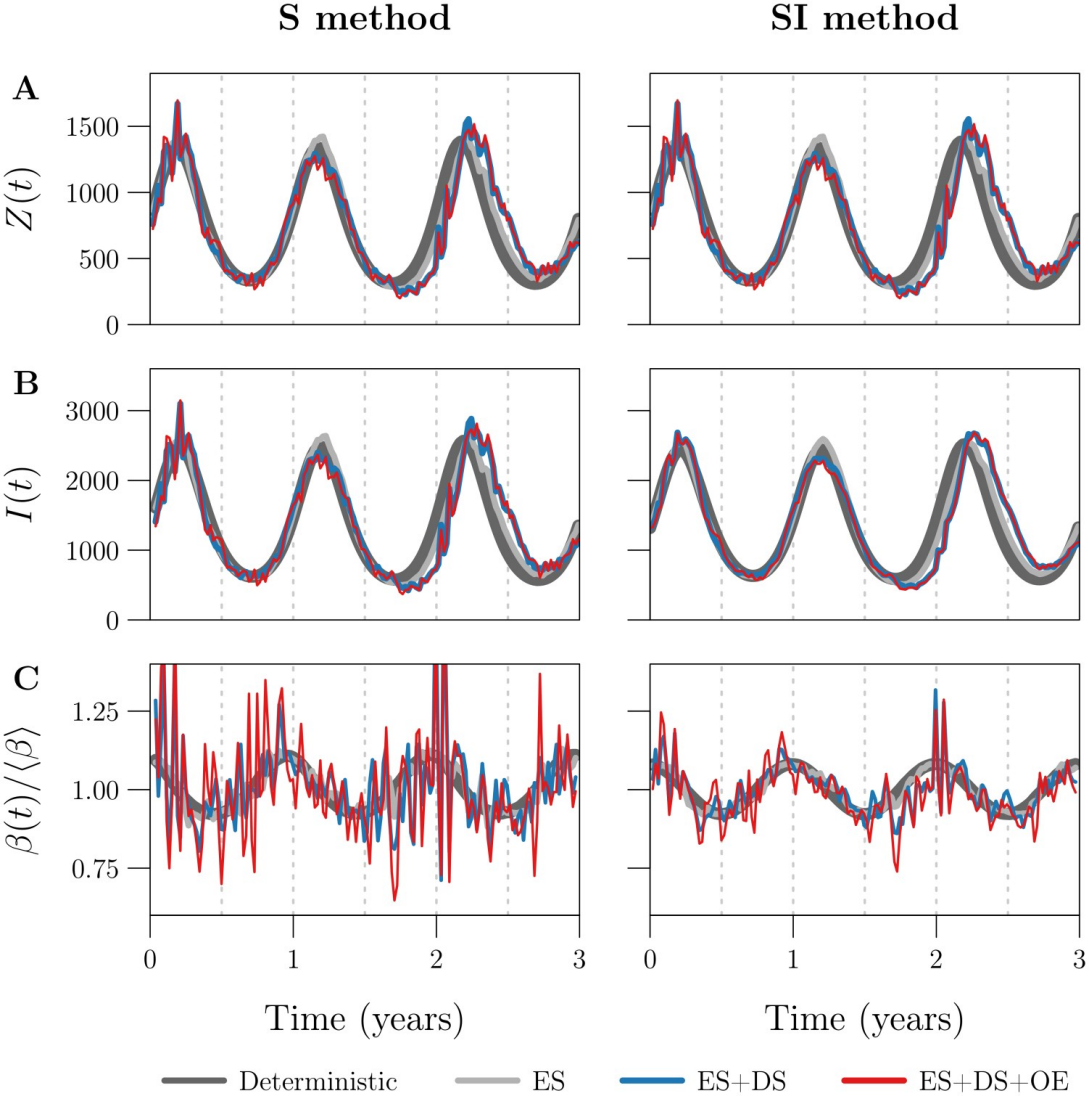
Effects of process and observation error on the S and SI methods. Plotted are the estimates **[Row A]** *Z*_*k*_, **[Row B]** *I*_*k*_, and **[Row C]** *β*_*k*_ of true incidence *Z*(*t*), prevalence *I*(*t*), and the seasonally forced transmission rate *β*(*t*) (Eq (27)) obtained by applying the **[Left]** S and **[Right]** SI methods without input error to each of four simulated reported incidence time series (indicated by the legend; Δ*t* = 1 week, *n* = ⌊3 × 365/7⌋ = 156). The first simulation was purely deterministic [dark grey] (*ϵ* = 0, *p*_rep_ = 1), while the remaining three accounted for (i) environmental stochasticity [ES, light grey] (*ϵ* = 0.5, *p*_rep_ = 1), (ii) ES and demographic stochasticity [ES+DS, blue] (*ϵ* = 0.5, *p*_rep_ = 1), or (iii) ES, DS, and observation error [ES+DS+OE, red] (*ϵ* = 0.5, *p*_rep_ = 0.25). Reference values (Table 1) were assigned to all other data-generating parameters, in all four simulations. The left and right panels in Row A are identical, because the S and SI methods compute *Z*_*k*_ identically (compare Eqs (25a) and (26a)). RRMSE in the *β*_*k*_ time series is (0.0239, 0.0375, 0.1126, 0.1432) with the S method and (0.0021, 0.0153, 0.0494, 0.0591) with the SI method (order follows the legend). Note that the underlying *β*(*t*) was the same in all simulations; it is not plotted in Row C, but is close to perfectly represented by the dark grey curve in the right panel (RRMSE ≈ 0.2%). Due to process error, the underlying *Z*(*t*) and *I*(*t*) (also not shown) varied between the deterministic, ES, and ES+DS simulations.

We applied the S and SI methods without input error to four reported incidence time series *C*_*k*_, simulated using the same parameter values but with different levels of process and observation noise. The first simulation was purely deterministic, while the remaining three included (i) environmental stochasticity [ES], (ii) ES and demographic stochasticity [ES+DS], or (iii) ES, DS, and observation error [ES+DS+OE]. Fig 2 shows the resulting estimates *Z*_*k*_, *I*_*k*_, and *β*_*k*_ of true incidence *Z*(*t*), prevalence *I*(*t*), and the seasonally forced transmission rate *β*(*t*).

Noise of any type introduces random fluctuations in *C*_*k*_ on top of longer-term (*e.g*., seasonal) variation. Noise in *C*_*k*_ is propagated to *Z*_*k*_ (Fig 2A) and *I*_*k*_ (Fig 2B), because (i) in both the S and SI methods, we scale *C*_*k*+*r*_ by a constant factor of $p_{\text{rep}}^{-1}\geq 1$ to compute *Z*_*k*_ (Eqs (25a) and (26a)); (ii) in the S method, we scale *C*_*k*+1−*g*+*r*_ by a constant factor of [*p*_rep_(*γ* + *μ*_k_)Δ*t*]^−1^ to compute *I*_*k*_ (Eq (25c) after substitution of Eq (25a)); and (iii) in the SI method, *I*_*k*_ contains a weighted sum of *C*_*i*_ terms (Eq (38b)).

Noise in *Z*_*k*_ and *I*_*k*_ is amplified in *β*_*k*_ (Fig 2C), distorting the correct temporal pattern, for the following reason. When *Z* and *I* are close to zero, small absolute changes in either yield large relative changes in the ratio *Z*/*I* and in turn *β*_*k*_, which contains a factor of *Z*_*k*+1_/*I*_*k*_ in the S method (Eq (25d)) and (*Z*_*k*_ + *Z*_*k*+1_)/(2*I*_*k*_) in the SI method (Eq (26d)). Hence low amplitude noise in *Z*_*k*_ and *I*_*k*_ appears as spurious, higher amplitude noise in *β*_*k*_. This is an important issue in practice, because the incidence of endemic diseases is typically very small relative to the population size, and periodic fluctuations bringing incidence even closer to zero are common for many diseases [4, 14, 42].

Fig 2 shows that the SI method is much better than the S method at resisting noise propagation. One reason is the effective smoothing of incidence in the SI method, which replaces *Z*_*k*+1_ with (*Z*_*k*_ + *Z*_*k*+1_)/2 in the computation of *β*_*k*_ (compare Eqs (25d) and (26d)). We expose a second reason in §3.2.1 below by comparing the variance in *I*_*k*_ induced by observation error, between the two methods. (We expect similar results for process error.)

#### 3.2.1 Propagation of noise from *C*_*k*_ to *I*_*k*_

Consider the S and SI method estimates of prevalence *I*(*t*_*k*_),

$$I_{k}^{\text{[S]}}=\frac{C_{k+1-g+r}}{p_{\text{rep}}\left(\gamma +\mu_{\text{c}}\right)\Delta t}\;,$$ (54a)

$$I_{k}^{\text{[SI]}}=I_{0}{\left(\frac{1-\frac{1}{2}\left(\gamma +\mu_{\text{c}}\right)\Delta t}{1+\frac{1}{2}\left(\gamma +\mu_{\text{c}}\right)\Delta t}\right)}^{k}+\sum_{i=0}^{k-1}\frac{C_{i+1+r}}{p_{\text{rep}}\left[1+\frac{1}{2}\left(\gamma +\mu_{\text{c}}\right)\Delta t\right]}{\left(\frac{1-\frac{1}{2}\left(\gamma +\mu_{\text{c}}\right)\Delta t}{1+\frac{1}{2}\left(\gamma +\mu_{\text{c}}\right)\Delta t}\right)}^{k-1-i}\;.$$ (54b)

Here, *g* = [*t*_gen_]_Δ*t*_/Δ*t* and *r* = [*t*_rep_]_Δ*t*_/Δ*t* are the mean generation interval and case reporting delay in units of the observation interval, rounded to the nearest integer. These estimates are obtained from Eq (25c) (after substitution of Eqs (25a)) and (38b) when we assume a constant natural mortality rate *μ*_c_. Following §2.3.3, suppose reported incidence is generated from true incidence *Z*(*t*_*k*_) via $C_{k+r}\overset{\text{ind.}}{∼}\text{Binomial}\left(Z\left(t_{k}\right),p_{\text{rep}}\right)$. Then the variance of *C*_*k*+*r*_ is

$$\begin{array}{c} \text{Var}\left(C_{k+r}\right)=Z\left(t_{k}\right)p_{\text{rep}}\left(1-p_{\text{rep}}\right)\;. \end{array}$$ (55)

It follows from Eqs (54) and (55) and the identity Var(*aX*) = *a*^2^ Var(*X*) that

$$\text{Var}(I_{k}^{\left[\text{S}\right]})=\frac{\left(1-p_{\text{rep}})Z(t_{k+1-g}\right)}{p_{\text{rep}}{\left[(\gamma +\mu_{\text{c}})\Delta t\right]}^{2}},$$ (56a)

$$Var(I_{k}^{\text{[SI]}})=\sum_{i=0}^{k-1}\frac{\left(1-p_{\text{rep}}\right)Z\left(t_{i+1}\right)}{p_{\text{rep}}{\left[1+\frac{1}{2}\left(\gamma +\mu_{\text{c}}\right)\Delta t\right]}^{2}}{\left(\frac{1-\frac{1}{2}\left(\gamma +\mu_{\text{c}}\right)\Delta t}{1+\frac{1}{2}\left(\gamma +\mu_{\text{c}}\right)\Delta t}\right)}^{2(k-1-i)}\;.$$ (56b)

If *Z*(*t*) has a well-defined average 〈*Z*〉, then replacing instances of *Z* in Eq (56) with 〈*Z*〉 and taking the limit as *k* → ∞, we obtain the average variances

$$\langle Var(I_{k}^{\text{[S]}})\rangle =\frac{(1-p_{\text{rep}})\langle Z\rangle }{p_{\text{rep}}{\left[(\gamma +\mu_{\text{c}})\Delta t\right]}^{2}},$$ (57a)

$$\begin{array}{cc} \left\langle Var(I_{k}^{\text{[SI]}})\right\rangle & =\underset{k\to \infty }{\text{lim}}\{\frac{\left(1-p_{\text{rep}}\right)\left\langle Z\right\rangle }{p_{\text{rep}}{\left[1+\frac{1}{2}\left(\gamma +\mu_{\text{c}}\right)\Delta t\right]}^{2}}\sum_{i=0}^{k-1}{\left(\frac{1-\frac{1}{2}\left(\gamma +\mu_{\text{c}}\right)\Delta t}{1+\frac{1}{2}\left(\gamma +\mu_{\text{c}}\right)\Delta t}\right)}^{2i}\}\\ & =\underset{k\to \infty }{\text{lim}}\{\frac{\left(1-p_{\text{rep}}\right)\left\langle Z\right\rangle }{2p_{\text{rep}}\left(\gamma +\mu_{\text{c}}\right)\Delta t}[1-{\left(\frac{1-\frac{1}{2}\left(\gamma +\mu_{\text{c}}\right)\Delta t}{1+\frac{1}{2}\left(\gamma +\mu_{\text{c}}\right)\Delta t}\right)}^{2k}]\}\\ & =\frac{\left(1-p_{\text{rep}}\right)\left\langle Z\right\rangle }{2p_{\text{rep}}\left(\gamma +\mu_{\text{c}}\right)\Delta t}\;. \end{array}$$ (57b)

Comparing these with 〈Var(*C*_*k*_)〉 = 〈*Z*〉*p*_rep_(1 − *p*_rep_) using reference parameter values *t*_gen_ = *γ*^−1^ = 13 days, *μ*_c_ = 0.04year^−1^, and Δ*t* = 1 week, we obtain

$$\frac{\left\langle \text{Var}(I_{k}^{\left[\text{S}\right]})\right\rangle }{\left\langle \text{Var}(C_{k}\text{)}\right\rangle }=\frac{1}{p_{\text{rep}}^{2}{\left[(\gamma +\mu_{\text{c}})\Delta t\right]}^{2}}=\frac{1}{p_{\text{rep}}^{2}}{\left(\frac{t_{\text{inf}}}{\Delta t}\right)}^{2}\approx \frac{3.44}{p_{\text{rep}}^{2}},$$ (58a)

$$\frac{\left\langle \text{Var}(I_{k}^{\left[\text{S}\right]})\right\rangle }{\left\langle \text{Var}(C_{k}\text{)}\right\rangle }=\frac{1}{2p_{\text{rep}}^{2}{\left[(\gamma +\mu_{\text{c}})\Delta t\right]}^{2}}=\frac{1}{2p_{\text{rep}}^{2}}{\left(\frac{t_{\text{inf}}}{\Delta t}\right)}^{2}\approx \frac{0.93}{p_{\text{rep}}^{2}},$$ (58b)

where *t*_inf_ = (*γ* + *μ*_c_)^−1^ is the mean time spent infected. Hence, while both the S and SI methods suffer from propagation of noise from reported incidence *C*_*k*_ to estimated prevalence *I*_*k*_, particularly for *p*_rep_ ≪ 1, the S method tends to be much worse (by a factor of 3.44/0.93 ≈ 3.7 in this example). Comparative resistance to noise propagation is a distinct advantage of the SI method over the S method.

### 3.3 Averaging the raw estimate of *β*(*t*)

**Fig 3:**
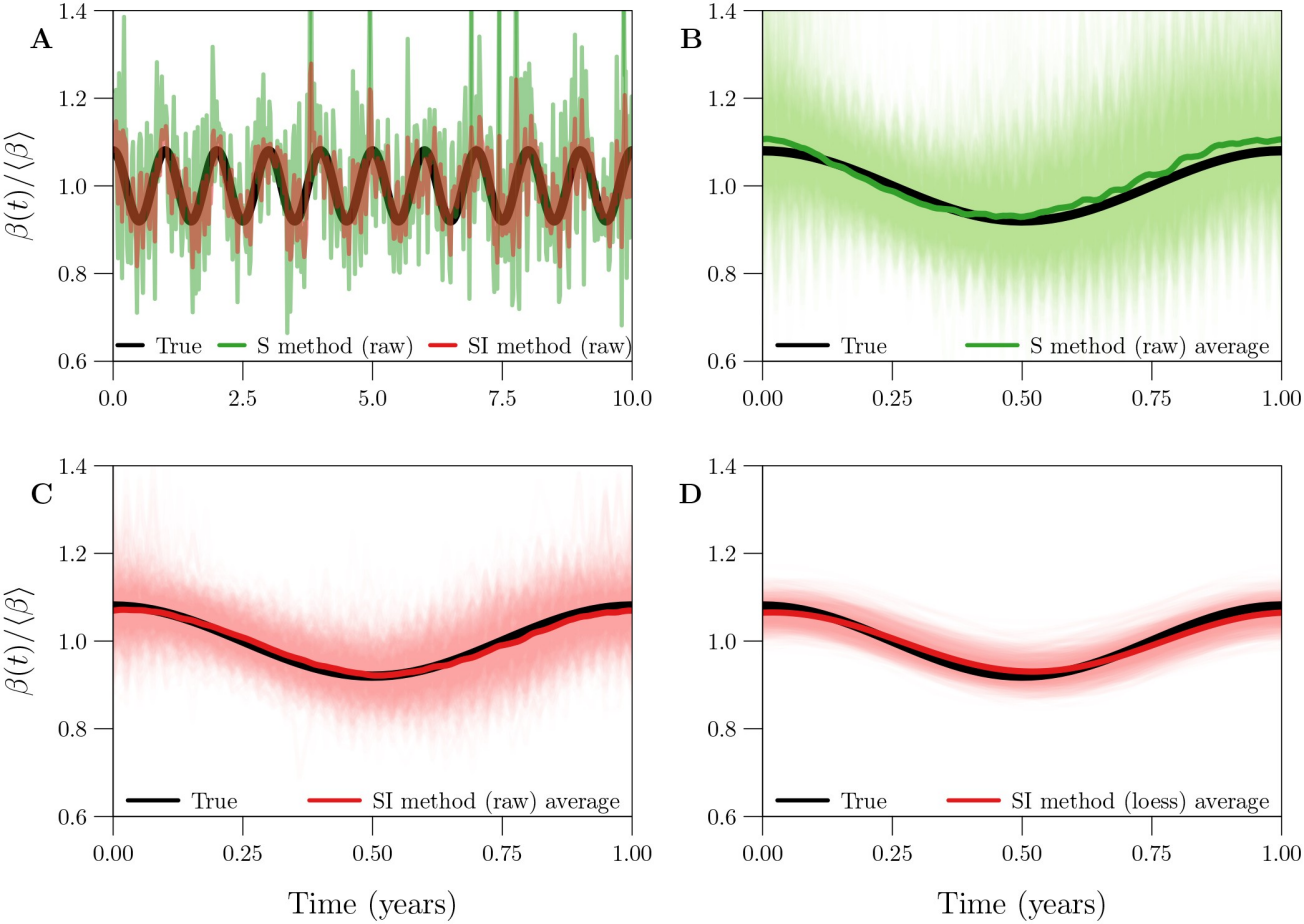
Bias and variance in 1-year cycles embedded in three estimates of a seasonally forced *β*(*t*). **[Panel A]** In black, the seasonally forced *β*(*t*) (Eq (27)) underlying 1000 years of simulated reported incidence data. In (transparent) colour, raw estimates *β*_*k*_ obtained from the data by the S [green] and SI [red] methods, both applied without input error. Only the first 10 of 1000 years are shown. **[Panels B and C]** In black, the true 1-year cycle in the seasonally forced *β*(*t*). In light (transparent) colour, the 1000 1-year cycles embedded in the linear interpolant *β*_int_(*t*) of *β*_*k*_. In dark colour, the average 1-year cycle (Eq (22a)) in *β*_int_(*t*). Results are shown for both the S [Panel B, green] and SI [Panel C, red] methods. **[Panel D]** Like Panel C, except for a smooth loess curve *β*_loess_(*t*; *q*) (*q* = 53) fit to *β*_*k*_, instead of the interpolant *β*_int_(*t*). **[Details]** A reported incidence time series with 1000 years of weekly observations (Δ*t* = 1 week, *n* = 52153) was simulated with environmental noise in transmission (*ϵ* = 0.5), demographic stochasticity, and random under-reporting of cases (*p*_rep_ = 0.25), using reference values (Table 1) for the remaining parameters.

Fig 3A displays two raw estimates *β*_*k*_ (S and SI methods, applied without input error) of a seasonally forced *β*(*t*), each spanning 1000 years (only the first 10 years are shown). The estimates embed 1000 1-year cycles, which are displayed in Fig 3B and 3C together with their 1-year average (*cf*. §2.2.5).

Both estimates suffered from spurious noise distorting the correct seasonal pattern, caused by process and observation error in the data-generating process (*cf*. §3.2). As in Fig 2C, the variance was markedly smaller with the SI method. Averaging the embedded 1-year cycles recovered the true 1-year cycle from the noise. In the absence of input error, the S method appears to carry a slight bias (peaking early and too high, as in Fig 1), whereas the SI method is nearly unbiased.

While some existing infectious disease time series span several centuries [15], in practice, averaging as in Fig 3B and 3C is sensible only over time intervals during which the underlying seasonal pattern in transmission is roughly stationary.

### 3.4 Smoothing the raw estimate of *β*(*t*)

Regardless of whether averaging is employed, comparison of Fig 3C and 3D shows that it is helpful to smooth the *β*_*k*_ time series by fitting a loess curve *β*_loess_(*t*; *q*) (*cf*. §2.2.6). An appropriate degree of smoothing (*i.e*., choice of loess smoothing parameter *q*) eliminated spurious noise without significantly increasing bias.

**Fig 4:**
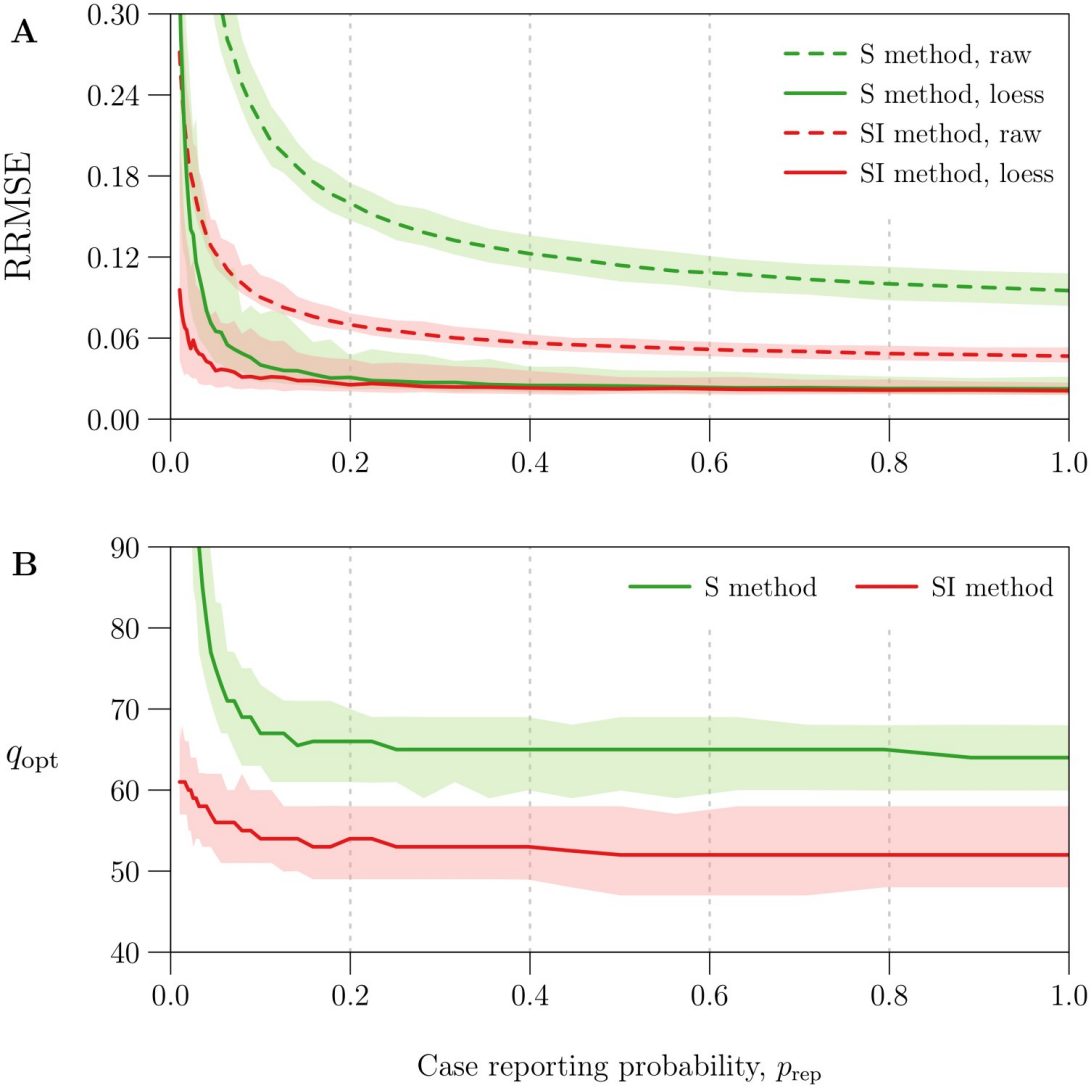
Reduction in *β*(*t*) estimation error with optimal loess smoothing. The horizontal axis measures the case reporting probability *p*_rep_, for which 41 values equally spaced on a logarithmic scale between 0.01 and 1 were considered. Using each value of *p*_rep_ and reference values (Table 1) for all other parameters, 100 reported incidence time series (Δ*t* = 1 week, *n* = 1042) were simulated accounting for environmental noise in transmission (*ϵ* = 0.5), demographic stochasticity, and random under-reporting of cases (measured by *p*_rep_). The underlying seasonally forced *β*(*t*) (Eq (27)) was estimated from reported incidence using the S and SI methods, both applied without input error, yielding two raw estimates *β*_*k*_ per simulation. Smooth loess curves *β*_loess_(*t*; *q*) (*q* = 10, …, 110; *cf*. §2.2.6) were fit to each *β*_*k*_ time series. The optimal *q* for a given time series, denoted by *q*_opt_, was defined as the value that minimized RRMSE (Eq (33)) in *β*_loess_(*t*_*k*_; *q*). Overall, for each value of *p*_rep_ and each *β*(*t*) estimation method (S and SI), 100 values of *q*_opt_ were obtained corresponding to 100 *β*_*k*_ time series. Plotted on the vertical axis as functions of *p*_rep_ are the median and 5th and 95th percentiles of **[Panel A]** RRMSE in the raw estimates *β*_*k*_ [dashed lines] and optimal loess estimates *β*_loess_(*t*_*k*_; *q*_opt_) [solid lines] and **[Panel B]** *q*_opt_. Lines and bands indicate the median and 5th–95th percentile range, respectively. Results for the S and SI methods are shown in green and red, respectively.

Fig 4A quantifies the effect of smoothing *β*_*k*_ using the optimal value *q*_opt_ for parameter *q* (*cf*. §2.2.6). It plots RRMSE before and after smoothing as a function of the amount of noise in the simulated reported incidence data, which was modulated by varying the case reporting probability *p*_rep_ between 0.01 and 1 (more noise for smaller *p*_rep_; see Eq (31)).

Using the optimal loess estimate *β*_loess_(*t*_*k*_; *q*_opt_) instead of the raw estimate *β*_*k*_ significantly reduced RRMSE—by at least 46% for the S method and 17% for the SI method across all simulations. Although raw estimates generated by the SI method were consistently more accurate (expected in light of Fig 3B and 3C), optimal loess estimates were comparable between the S and SI methods for *p*_rep_ > 0.2 (RRMSE ≈ 3%). For *p*_rep_ < 0.2 (severe under-reporting of cases), optimal smoothing failed to an increasing extent to recover the underlying *β*(*t*) from noise in *β*_*k*_. In this setting, the S method was greatly outperformed by the SI method, which is more resilient to noise in reported incidence (*cf*. §3.2).

Fig 4B shows that median *q*_opt_ was roughly constant for *p*_rep_ > 0.1, with

$$\begin{array}{c} \text{median}\;q_{\text{opt}}\approx \{\begin{array}{cc} 65 & \text{for}\;\text{the}\;\text{S}\;\text{method}\;,\\ 53 & \text{for}\;\text{the}\;\text{SI}\;\text{method}\;. \end{array} \end{array}$$ (59)

More smoothing (greater *q*) was required to minimize RRMSE for *p*_rep_ > 0.1. More generally, Fig 4 indicates that the S and SI methods should always include a smoothing step. Hence, in the remaining analysis, we always smooth *β*_*k*_.

### 3.5 Sensitivity to data-generating parameters

Here, we characterize the sensitivity of *β*(*t*) estimation error to parameters of the data-generating process. As in §§3.1–3.4, we consider the ideal case in which the user-specified values of all input parameters are equal to the true (data-generating) values. The details of our analysis are outlined in §2.6.1.

**Fig 5:**
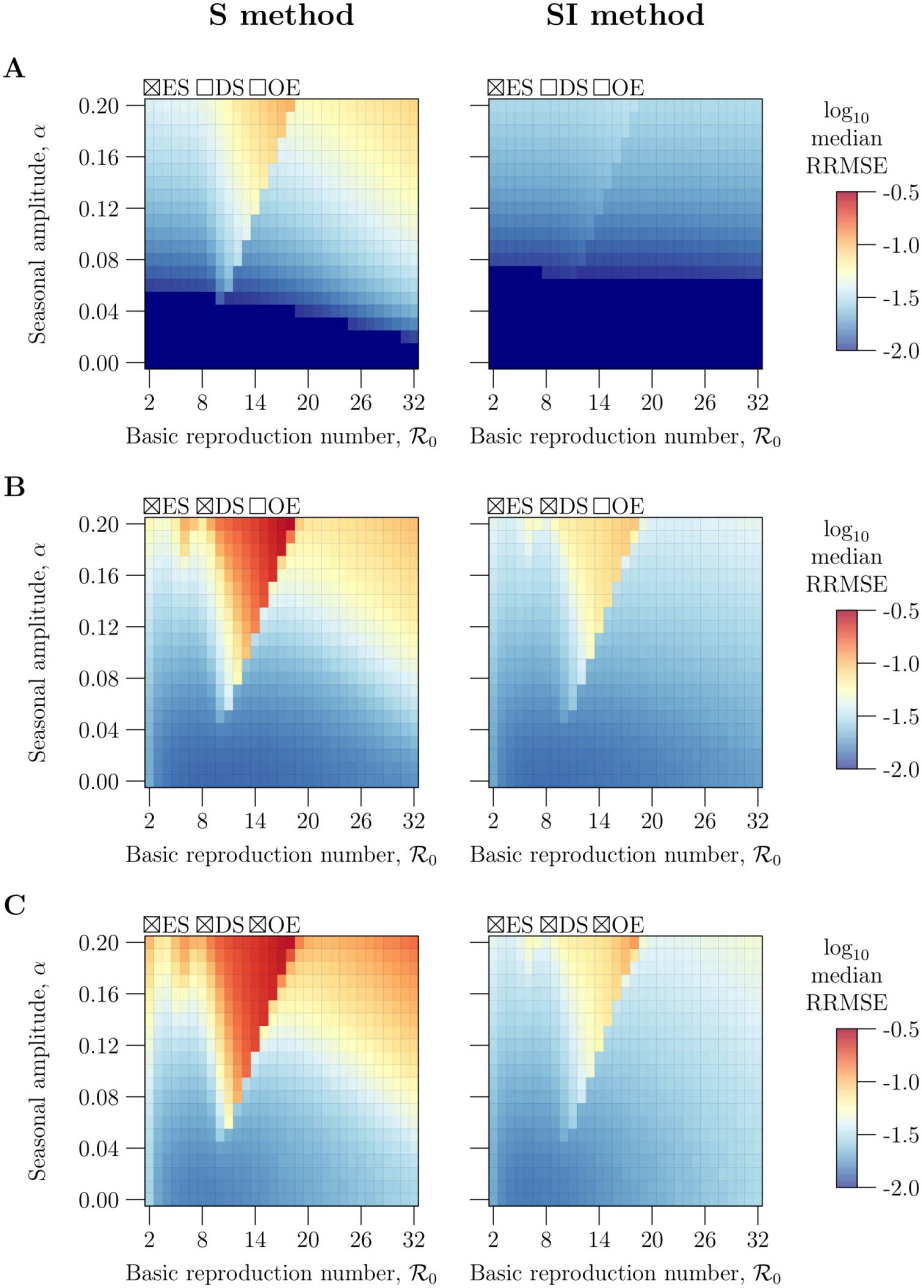
Sensitivity of *β*(*t*) estimation error to the mean 〈*β*〉 and amplitude *α* of seasonal forcing. Contained in each panel are heatmaps of median RRMSE (Eq (33)) in estimates of a seasonally forced *β*(*t*) (Eq (27)) from simulated reported incidence time series, as a bivariate function of the mean 〈*β*〉 and amplitude *α* of seasonal forcing. The 〈*β*〉 axis has been scaled to measure the basic reproduction number $R_{0}$ (Eq (2)). When simulating reported incidence, reference values (Table 1) were assigned to all data-generating parameters except 〈*β*〉 and *α*. A grid of $\left(R_{0},\alpha \right)$ pairs with levels $R_{0}=2,3,\ldots ,32$ and *α* = 0, 0.01, …, 0.2 was considered, with 〈*β*〉 defined for each value of $R_{0}$ via Eq (2). For each parametrization, 1000 simulations were performed with environmental stochasticity [ES] (*ϵ* = 0.5) and with or without demographic stochasticity [DS] and observation error [OE], as indicated by row: **[Row A]** without DS or OE (*p*_rep_ = 1, *t*_rep_ = 0 weeks), **[Row B]** with DS but without OE (*p*_rep_ = 1, *t*_rep_ = 0 weeks), **[Row C]** with DS and OE (*p*_rep_ = 0.25, *t*_rep_ = 2 weeks). Corresponding mock birth and natural mortality time series were created, then *β*(*t*) was estimated from the data using **[Left]** the S method and **[Right]** the SI method, all without input error. For each set of estimates of *β*(*t*) (1000 estimates per parametrization, per simulation method, per estimation method), the median RRMSE was calculated (after smoothing with fixed *q*; see Eq (59)) and displayed as one point in the appropriate heatmap, coloured according to the logarithmic scale on the right. The darkest blue indicates median RRMSE less than 0.01.

Fig 5 plots the median RRMSE in estimates of a seasonally forced *β*(*t*) (Eq (27)) from 1000 realizations of a reported incidence time series, as a bivariate function of the mean 〈*β*〉 and amplitude *α* of seasonal forcing. To aid interpretation, the 〈*β*〉 axis was scaled to measure the basic reproduction number $R_{0}$ (Eq (2)).

**Fig 6:**
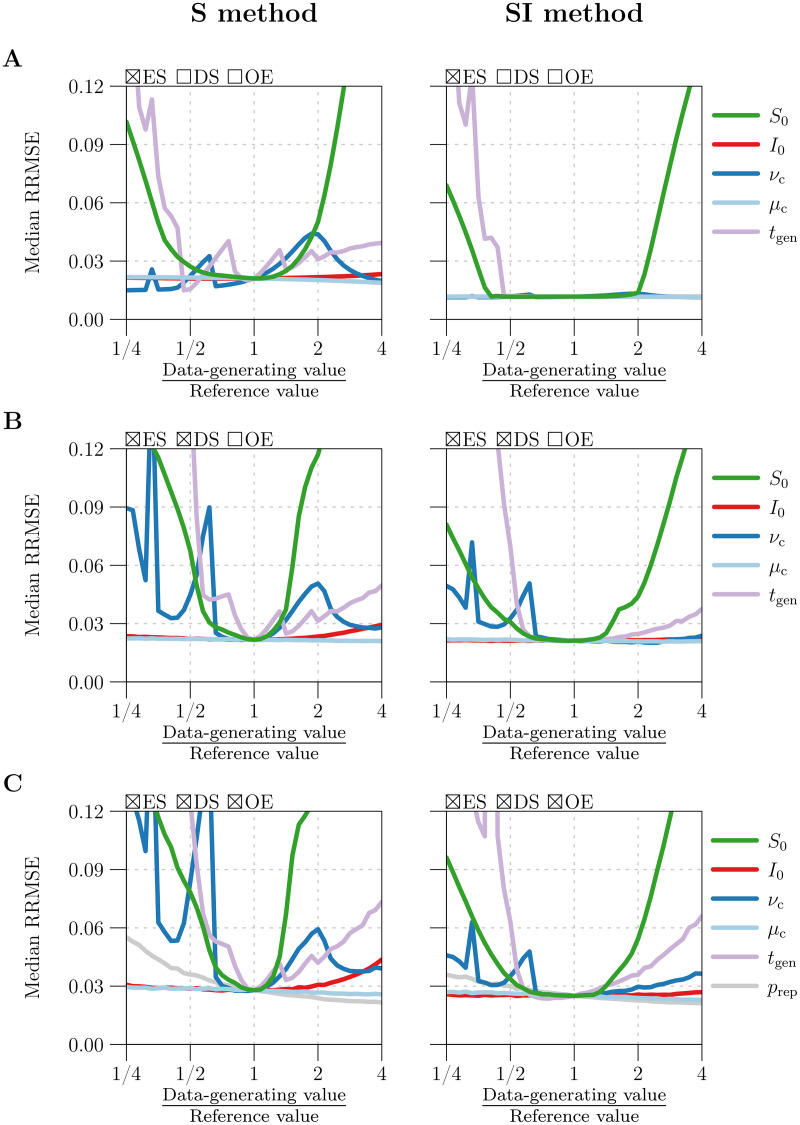
Sensitivity of *β*(*t*) estimation error to data-generating parameters other than 〈*β*〉 and *α*. Plotted in each panel is the median RRMSE (Eq (33)) in estimates of a seasonally forced *β*(*t*) (Eq (27)) from simulated reported incidence time series (Δ*t* = 1 week, *n* = 1042), as a univariate function of each of 5 or 6 data-generating parameters (indicated by the legend). When simulating reported incidence, reference values (Table 1) were assigned to all but the focal parameter, which was assigned 41 values logarithmically spaced between $\frac{1}{4}$ and 4 times its reference value. The horizontal axis (logarithmic scale) measures the ratio of the focal parameter’s true value to its reference value, so that commensurate deviations from the reference case can be compared across parameters. For each parametrization, 1000 simulations were performed with environmental stochasticity [ES] (*ϵ* = 0.5) and with or without demographic stochasticity [DS] and observation error [OE], as indicated by row: **[Row A]** without DS or OE (*p*_rep_ = 1, *t*_rep_ = 0 weeks), **[Row B]** with DS but without OE (*p*_rep_ = 1, *t*_rep_ = 0 weeks), or **[Row C]** with DS and OE (*p*_rep_ = 0.25 except when *p*_rep_ is the focal parameter, *t*_rep_ = 2 weeks). Corresponding mock birth and natural mortality time series were created, then *β*(*t*) was estimated from the data using **[Left]** the S method and **[Right]** the SI method, all without input error. For each set of estimates of *β*(*t*) (1000 estimates per parametrization, per simulation method, per estimation method), the median RRMSE was calculated (after smoothing with fixed *q*; see Eq (59)) and displayed as one point in the appropriate panel and graph.

Fig 6 plots median RRMSE as a univariate function of each of 6 additional parameters—the initial states *S*_0_ and *I*_0_, vital rates *ν*_c_ and *μ*_c_, mean generation interval *t*_gen_, and case reporting probability *p*_rep_—with the focal parameter assigned values between $\frac{1}{4}$ and 4 times its reference value (Table 1). The horizontal axis measures the ratio of the focal parameter’s data-generating value to its reference value, so that commensurate deviations from the reference case can be compared across the 6 parameters.

In order to produce Figs 5 and 6, we assigned reference values (Table 1) to all but the focal data-generating parameter(s) (*e.g*., all except 〈*β*〉 and *α* in Fig 5). We fit loess curves *β*_loess_(*t*; *q*) to all raw estimates *β*_*k*_ of *β*(*t*), and recorded the RRMSE in *β*_loess_(*t*_*k*_; *q*). Motivated by Fig 4B and Eq (59), we fixed *q* = q*, taking q* = 65 with the S method and q* = 53 with the SI method.

A pattern in our interpretation of Figs 5 and 6 below is that error in *β*(*t*) estimation is sensitive to a parameter if changes in that parameter (i) cause incidence *Z*(*t*) or prevalence *I*(*t*) to approach zero more frequently or more closely, or (ii) increase noise in estimated incidence *Z*_*k*_ or estimated prevalence *I*_*k*_. Both outcomes incorrectly increase noise in *β*_*k*_ (*cf*. §3.2).

When the noise in *β*_*k*_ is extreme, setting *q* = q* can undersmooth the time series (q* < *q*_opt_). In this case, smaller RRMSE is attainable by determining *q*_opt_ and setting *q* = *q*_opt_. Nevertheless, we did not find *q*_opt_ for each of the 5 × 10^6^ time series considered by Figs 5 and 6, which would have increased the total computation time by a factor of 100. Consequently, Figs 5 and 6 may overestimate the sensitivity of *β*(*t*) estimation error to data-generating parameters. (In §S5.3 of S1 Text, we show that the quantitative effect of choosing q* over *q*_opt_ is likely to be small.)

#### 3.5.1 Sensitivity to the basic reproduction number $R_{0}$ and seasonal amplitude *α* (Fig 5)

For fixed *α*, median RRMSE was a non-monotonic function of $R_{0}$. The reason is that changes in (effective) $R_{0}$ are responsible for dynamical transitions that alter the structure of solutions of the SIR model (1) [28, 42, 43]. Specifically, as $R_{0}$ is increased from 2 to 32, minimum incidence *Z*_min_ and minimum prevalence *I*_min_ on the attractor varies non-monotonically (see Fig 2 in [28]). Smaller *Z*_min_ and *I*_min_ yield more noise in *β*_*k*_, and correspondingly greater RRMSE. For fixed $R_{0}$, *I*_min_ decreases monotonically as *α* is increased from 0 to 1 (see Fig 11 in [43]), so we expect median RRMSE to increase monotonically with *α*, as observed in Fig 5.

#### 3.5.2 Sensitivity to the initial state (*S*_0_, *I*_0_) (Fig 6)

RRMSE is sensitive to the data-generating *S*_0_, but not *I*_0_. The reference values of *S*_0_ and *I*_0_ are taken from a point (S*, I*, R*) on the attractor of the SIR model (1) with seasonally forced *β*(*t*) and constant vital rates *ν*_c_ and *μ*_c_ (*cf*. §2.3.4). When *S*_0_ is far from S*, the solution of system (1) undergoes extreme fluctuation before relaxing to the attractor, and both *Z* and *I* approach zero during the transient, generating spurious noise at the start of the *β*_*k*_ time series.

Note that *I*_0_ differing from I* has a much smaller effect on dynamics than *S*_0_ differing from S* by the same factor. Since I* ≪ S*, the perturbation of (*S*_0_, *I*_0_, *R*_0_) from the attractor is much smaller.

#### 3.5.3 Sensitivity to vital rates *ν*_c_ and *μ*_c_ (Fig 6)

Median RRMSE was a non-monotonic function of the data-generating birth rate *ν*_c_. This behaviour arises because scaling *ν*_c_ is dynamically equivalent to scaling $R_{0}$ by the same factor [2, 28], and median RRMSE is a non-monotonic function of $R_{0}$ (*cf*. §3.5.1 above).

Changing the data-generating natural mortality rate *μ*_c_ had a negligible effect on RRMSE. This is unsurprising, because natural death is dominated by recovery and disease-induced death in governing the rate of infected decrease. That is, *γ* ≫ *μ*(*t*) in Eq (1b), so changes in *μ*_c_ by up to a factor of 4 have little effect on dynamics.

#### 3.5.4 Sensitivity to the mean generation interval *t*_gen_ (Fig 6)

Median RRMSE increased rapidly as the data-generating *t*_gen_ was made smaller than 2^−4/5^ (roughly 0.57) times its reference value of 13 days. A period-doubling bifurcation occurs near this value of *t*_gen_, and the attractor of the SIR model (1) acquires a 2-year cycle with much smaller *Z*_min_ and *I*_min_ (see §S5.3.1 of S1 Text). Propagation of noise to *β*_*k*_ intensifies, resulting in greater RRMSE.

The performance of the S method fluctuates more as a function of *t*_gen_ than that of the SI method. This occurs because the S method rounds *t*_gen_ in the numerator of Eq (25c) to the nearest integer multiple of Δ*t*, and the rounding error oscillates as a function of *t*_gen_. The SI method does not require rounding, so these fluctuations are not observed.

#### 3.5.5 Sensitivity to the case reporting probability *p*_rep_ (Fig 6)

When the reported incidence data contain observation error (Fig 6C), RRMSE is additionally sensitive to the case reporting probability *p*_rep_. Decreasing *p*_rep_ increases noise in reported incidence *C*_*k*_ (Eq (31)), which is propagated to estimated incidence *Z*_*k*_, estimated prevalence *I*_*k*_, and in turn *β*_*k*_ (*cf*. §3.2).

Fig 6 suggests weak sensitivity to *p*_rep_. However, noise in *Z*_*k*_ and *I*_*k*_ is amplified in *β*_*k*_ *to the extent that* *Z* and *I* are close to zero (*cf*. §3.2). Hence, for example, if the data-generating *t*_gen_ were assigned a value smaller than half its reference value of 13 days, then we would have observed more acute sensitivity to *p*_rep_ as a result of closer approaches to zero by *Z* and *I* (*cf*. §3.5.4 above).

#### 3.5.6 S method versus SI method (Figs 5 and 6)

Both the S and SI methods performed well, estimating *β*(*t*) with median RRMSE less than 10% across most parametrizations. However, by resisting noise propagation (*cf*. §3.2), the SI method was significantly less sensitive to the data-generating parameters and to the addition of demographic stochasticity and observation error.

### 3.6 Sensitivity to mis-specification of input parameters

In §3.5, we considered the ideal situation in which the user knows the true (data-generating) values of the input parameters. Here, we examine the more realistic situation in which the user specifies input parameters with some error. The effect of mis-specification is particularly important for parameters that are difficult to estimate accurately, such as the case reporting probability *p*_rep_. The details of our analysis are outlined in §2.6.2.

We restrict our attention to application of the SI method to reported incidence data simulated with process and observation error. Differences in RRMSE between methods of data simulation and *β*(*t*) estimation are dominated (by an order of magnitude) by the increase in RRMSE resulting from mis-specified input parameters.

**Fig 7:**
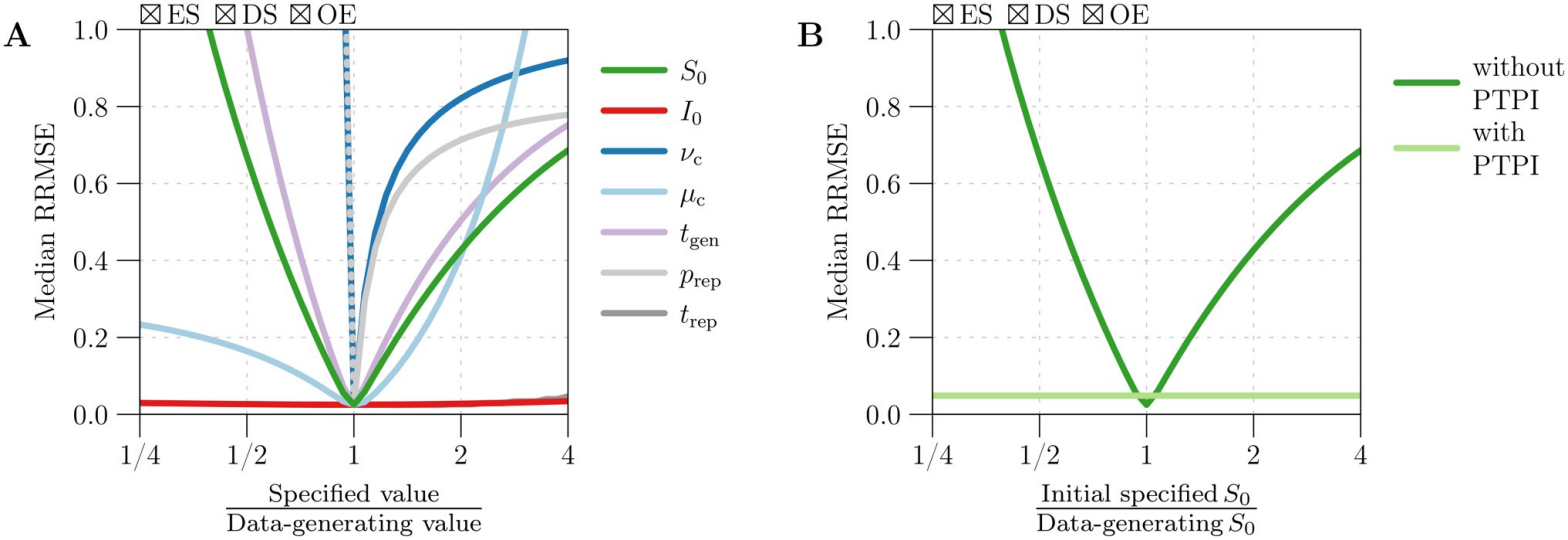
Sensitivity of *β*(*t*) estimation error to the user-specified values of input parameters. **[Panel A]** Median RRMSE (Eq (33)) in estimates of *β*(*t*) from simulated reported incidence time series (Δ*t* = 1 week, *n* = 1042), as a univariate function of the factor by which an input parameter was mis-specified. One thousand simulations were performed using fixed values (Table 1) for all data-generating parameters. The simulations accounted for environmental stochasticity [ES] (*ϵ* = 0.5), demographic stochasticity [DS], and observation error [OE] (*p*_rep_ = 0.25, *t*_rep_ = 2 weeks). For each simulation, corresponding mock birth and natural mortality time series were created, and *β*(*t*) was estimated from the data using the SI method. True (data-generating) values were specified for all input parameters except the focal parameter (indicated by the legend), for which 41 values logarithmically spaced between $\frac{1}{4}$ and 4 times the true value were specified in turn. Each input parametrization yielded 1000 estimates of *β*(*t*), whose median RRMSE was calculated (after smoothing with fixed *q*; see Eq (59)) and displayed as one point in the appropriate graph. **[Panel B]** Result of repeating the analysis from Panel A in which *S*_0_ was specified with varying amounts of error, but with the initially erroneous value of *S*_0_ updated using the method of peak-to-peak iteration (PTPI; 25 iterations) prior to *β*(*t*) estimation. The original result, obtained without PTPI, is presented for comparison.

Fig 7A plots the median RRMSE in estimates of *β*(*t*) from 1000 realizations of a reported incidence time series, as a univariate function of the factor by which an input parameter—one of the initial states *S*_0_ and *I*_0_, mean generation interval *t*_gen_, vital rates *ν*_c_ and *μ*_c_, and case reporting parameters *p*_rep_ and *t*_rep_—was mis-specified. The specified value of the focal parameter was varied between $\frac{1}{4}$ and 4 times its true (data-generating) value, and the remaining parameters were specified without error.

#### 3.6.1 Sensitivity to error in the specified initial state $\left(S_{0}^{\prime },I_{0}^{\prime }\right)$

Fig 7 shows that error in the specified value of *S*_0_ is propagated non-negligibly to estimates of *β*(*t*), while mis-specification of *I*_0_ has practically no effect on *β*(*t*) estimation error. Eqs (40) and (41) show that specifying incorrect values $S_{0}^{\prime }$ and $I_{0}^{\prime }$ for *S*_0_ and *I*_0_ creates errors in *S*_*k*_ and *I*_*k*_ that vanish geometrically as *k* → ∞. However, since *t*_life_ ≫ *t*_inf_, the decay is significantly slower in *S*_*k*_. Indeed, with reference values *μ*_c_ = 0.04 year^−1^, *t*_gen_ = *γ*^−1^ = 13 days, and Δ*t* = 1 week, we find that a factor of 10 reduction in error between times *t*_*k*_ and *t*_*k*+*i*_ requires just *i* = 5 in the infected time series, compared to *i* = 3002 in the susceptible time series (roughly 58 years with Δ*t* = 1 week). Hence, in practice, accurate reconstruction of *S*(*t*), *I*(*t*), and in turn *β*(*t*) relies on accurate specification of *S*_0_, but not *I*_0_. We address sensitivity to mis-specification of *S*_0_ in §3.7 below.

#### 3.6.2 Sensitivity to error in the specified birth rate $\nu_{\text{c}}^{\prime }$ and case reporting probability $p_{\text{rep}}^{\prime }$

Mis-specifying *ν*_c_ or *p*_rep_ by a factor of 2^1/10^ (7.2%) yielded median RRMSE greater than 30%. Mis-specifying by a factor of 2^−1/10^ (−6.7%) led to even worse estimates of *β*(*t*), with median RRMSE exceeding 100% (not visible in Fig 7A). Eqs (42) and (47) show that specifying incorrect values $\nu_{\text{c}}^{\prime }$ and $p_{\text{rep}}^{\prime }$ for *ν*_c_ and *p*_rep_ generates absolute errors in *S*_*k*_ that tend to increase over time (*k*) to a limit. In practice, systematic underestimation of births by the *B*_*k*_ time series (modeled here by $\nu_{\text{c}}^{\prime }<\nu_{\text{c}}$) and overestimation of incidence by the *Z*_*k*_ time series ($p_{\text{rep}}^{\prime }<p_{\text{rep}}$) can cause *S*_*k*_ to eventually take negative values. Once this happens, attempts by the S and SI methods to reconstruct *β*(*t*) fail completely.

While this failure may seem concerning, it should be viewed as a tool for diagnosing incorrect birth and case reporting rates: if the S or SI method yields negative *S*_*k*_ for any *k*, then one should speculate that births were underestimated or that incidence was overestimated, and retry the algorithm with a scaled up *B*_*k*_ time series and/or with greater *p*_rep_ (as *Z*_*k*_ is computed by scaling reported incidence by a factor of $p_{\text{rep}}^{-1}$; see Eqs (25a) and (26a)). Of course, overcorrection is also undesirable (*cf*. right half of Fig 7A). In our work, we have found that a brief exploration of possible adjustments—factors by which to increase *B*_*k*_ and/or *p*_rep_—suffices to identify ones that prevent both negative *S*_*k*_ and pronounced transient dynamics at the start of the susceptible time series (indicating under- or overcorrection).

### 3.7 Solution of the *S*_*0*_ estimation problem using PTPI

In §3.6, we showed that the performance of the S and SI methods is highly sensitive to mis-specification of the initial number of susceptibles *S*_0_. Here, we assess PTPI as a way to iteratively improve initially poor estimates of *S*_0_ prior to reconstruction of *S*(*t*) and *β*(*t*).

**Fig 8:**
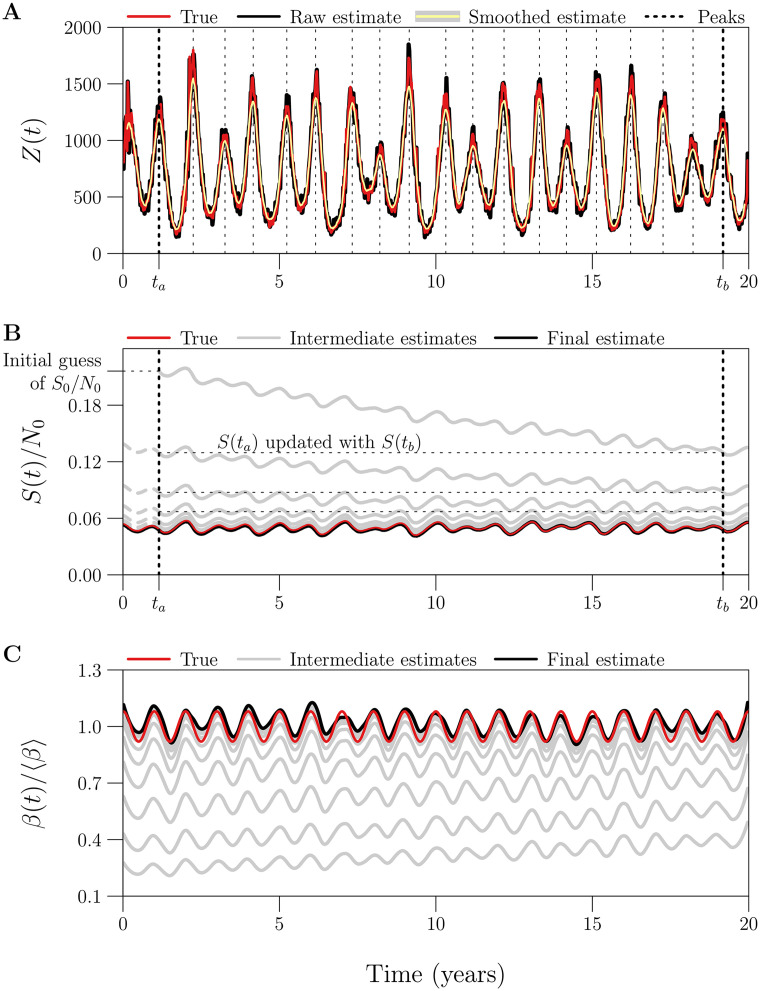
Example of *S*(*t*) and *β*(*t*) reconstruction with an overestimate of *S*_0_ corrected by peak-to-peak iteration (PTPI). **[Panel A]** Truncation step of PTPI (Box 5). Plotted is a reconstruction of true incidence *Z*(*t*) from a simulated reported incidence time series, before [*Z*_*k*_, black] and after [${\bar{Z}}_{k}$, yellow] smoothing with a 13-point central moving average. Vertical lines indicate peaks in ${\bar{Z}}_{k}$. The times of the first peak in ${\bar{Z}}_{k}$ and the last peak occurring at the same phase of the cycle (in this case, the last peak) are denoted by *t*_*a*_ and *t*_*b*_. **[Panel B]** Iteration step of PTPI (Box 6), where the initial estimates of both *S*_0_ = *S*(0) and *S*(*t*_*a*_) were taken to be 4 times the true (data-generating) value of *S*_0_. Plotted in grey are successive reconstructions of *S*(*t*) between times *t*_*a*_ and *t*_*b*_, generated by updating the estimate of *S*(*t*_*a*_) with the estimate of *S*(*t*_*b*_) obtained in the previous iteration. Dashed continuations to the left of *t*_*a*_ display estimation of *S*_0_ backwards in time from estimates of *S*(*t*_*a*_). Plotted in black is the result of reconstructing *S*(*t*) starting from the final estimate of *S*_0_, which was obtained after 25 iterations and had a relative error of roughly 1.4% (compared to 300% in the initial estimate). **[Panel C]** The sequence of reconstructions of *β*(*t*) corresponding to the estimates of *S*_0_ shown in Panel B. **[Details]** Twenty years of weekly reported incidence (Δ*t* = 1 week, *n* = 1042) were simulated with environmental noise in transmission (*ϵ* = 0.5), demographic stochasticity, and random under-reporting of cases (*p*_rep_ = 0.25), using reference values (Table 1) for the remaining parameters. *Z*(*t*), *S*(*t*) and *β*(*t*) were reconstructed from reported incidence using the SI method without input error (apart from mis-specification of *S*_0_).

**Fig 9:**
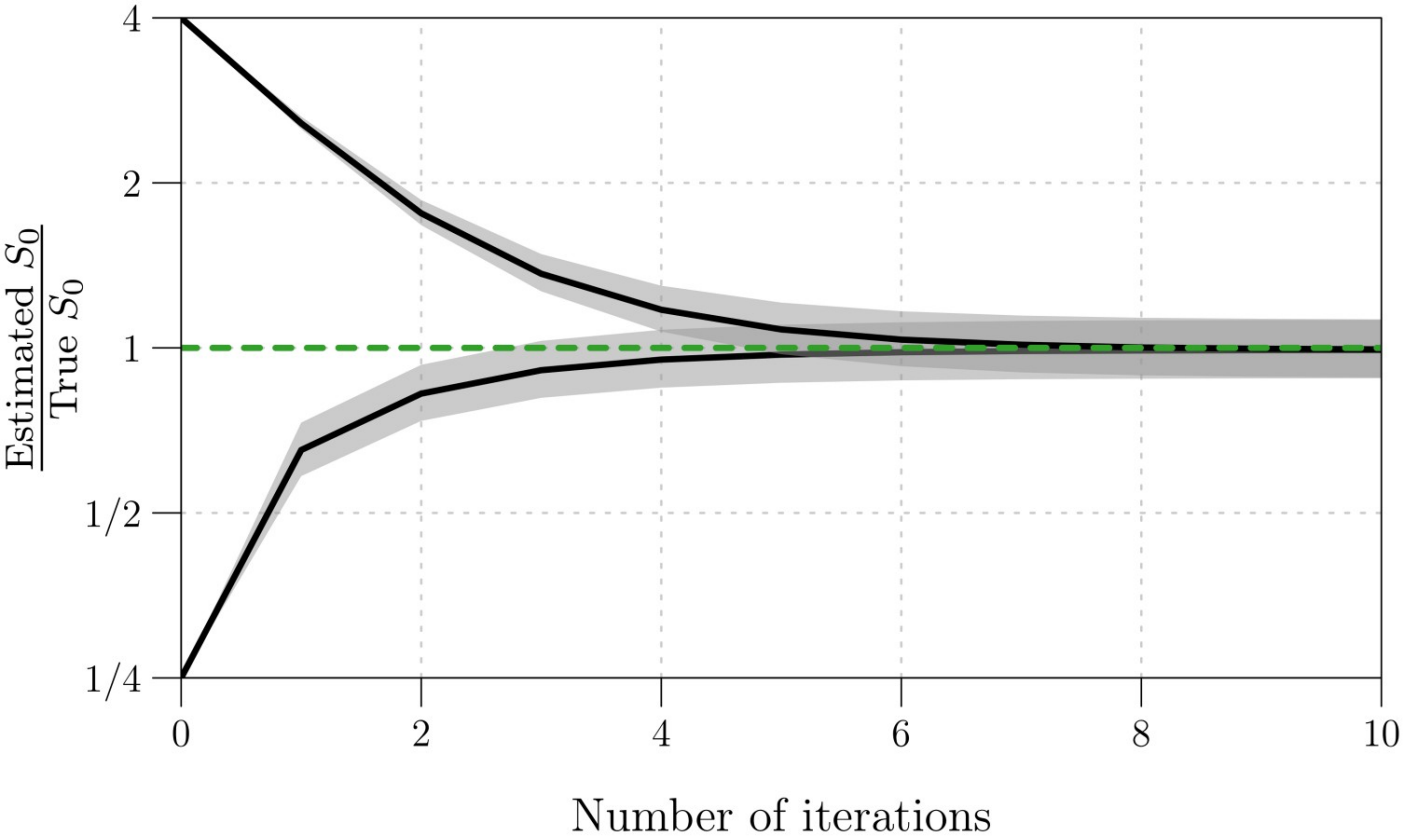
Convergence of estimates of *S*_0_ obtained using peak-to-peak iteration (PTPI). *S*_0_ was estimated by applying PTPI (25 iterations) to 1000 incidence time series (*i.e*., 1000 realizations of a reported incidence time series, scaled by $p_{\text{rep}}^{-1}$). An initial guess for *S*_0_ was taken to be $\frac{1}{4}$ or 4 times the true (data-generating) value. For each initial guess, this process generated 1000 sequences of 26 estimates of *S*_0_. Plotted are the median [black lines] and 5th–95th percentile range [grey bands] of the estimate of *S*_0_ at each iteration, for the first 10 iterations. The vertical axis measures (on a logarithmic scale) the ratio of the estimated and true values of *S*_0_, hence convergence close to 1 [dashed green line] represents convergence of the estimates close to the true value. **[Details]** One thousand reported incidence time series (Δ*t* = 1 week, *n* = 1042) were simulated with environmental noise in transmission (*ϵ* = 0.5), demographic stochasticity, and random under-reporting of cases (*p*_rep_ = 0.25), using reference values (Table 1) for the remaining parameters, including *S*_0_ (hence *S*_0_ was the same in all simulations). True incidence was estimated from reported incidence via Eq (26a) (with reporting parameters *p*_rep_ and *t*_rep_ correctly specified), yielding 1000 time series of estimated incidence. Corresponding mock (constant) birth and natural mortality time series were created (with vital rates *ν*_c_ and *μ*_c_ correctly specified), and these data (estimated incidence, births, natural mortality) were passed to the PTPI algorithm, allowing for iterative re-estimation of *S*_0_.

Fig 8 demonstrates PTPI for an example in which *S*_0_ was overestimated by a factor of 4 by a user of the SI method. PTPI yielded increasingly accurate estimates of *S*_0_ and correspondingly more accurate reconstructions of *S*(*t*) (Fig 8B) and *β*(*t*) (Fig 8C). Fig 7B repeats our analysis from §3.6, except using PTPI (25 iterations) to update the incorrect estimate of *S*_0_ prior to reconstructing *β*(*t*). We see that application of PTPI in conjunction with the SI method enables accurate *β*(*t*) reconstruction independently of errors in the initial estimate of *S*_0_. This result is unsurprising in light of Fig 9, which shows that PTPI converges rapidly (in fewer than 10 iterations) to an accurate estimate of *S*_0_ independently of the initial guess. Due to process error in the underlying dynamics, the relative error in the limiting estimate of *S*_0_ varied between the 1000 realizations of reported incidence considered (5th–95th percentile range [−11.9, 12.5]%, median 0.9%). Process error creates variance in the time between peaks in incidence (see Fig 8A), violating the periodicity assumption of PTPI (the theoretical basis of the technique; *cf*. §2.8). Nevertheless, Figs 7–9 demonstrate that PTPI can significantly improve *S*(*t*) and *β*(*t*) reconstruction from roughly periodic incidence data.

### 3.8 Run time

We implemented the S and SI methods and PTPI in R and ran them on a MacBook Pro with a 2.4 GHz Quad-Core Intel Core i5 chip. The S and SI methods are both extremely fast, requiring a total of 0.124 and 0.376 seconds, respectively, to generate a reconstruction of *β*(*t*) from 1000 years of weekly reported incidence (Δ*t* = 1 week, *n* = 52142). Application of PTPI in conjunction with either method increases the run time with each iteration, but the total run time remains inconsequential due to the rate of convergence of the iterations to a limiting estimate of *S*_0_. For example, when we applied PTPI to the same simulated data, the truncation step (Box 5) added 0.094 seconds to the total run time, while the iteration step (Box 5) added 1.01 seconds per iteration on average.

## 4 Discussion

We have compared three fast methods of estimating the time-varying transmission rate *β*(*t*) from reported incidence time series, all based on discretizations of the SIR model (1). Fine and Clarkson’s method [6], referred to here as the FC method, fails rapidly in practice, because it treats natural mortality in the susceptible population as negligible. Although Krylova’s method [24], adapted here as the S method, corrects this limitation of the FC method and is accurate for certain simulated data, her method suffers from extreme sensitivity to process and observation error. Specifically, noise in reported incidence is spuriously propagated to its estimates of *β*(*t*). Our algorithm for transmission rate estimation, referred to here as the SI method and based on deJonge’s method [25], is much more resilient to noise in reported incidence and therefore superior to the S method.

Like its predecessors, the SI method is sensitive to (i) certain input parameters: the initial number of susceptible individuals *S*_0_, the case reporting probability *p*_rep_, and the mean generation interval *t*_gen_; as well as (ii) vital data: times series of births and natural mortality without substantial systematic errors.

The requirement of a good estimate of *S*_0_ has been a major barrier to use of existing fast methods of *β*(*t*) estimation (including those presented in [6, 24, 25]). We have proposed and demonstrated PTPI as a valid and fast technique for obtaining accurate estimates of *S*_0_ from poor initial guesses, conditional on periodic dynamics (epidemic recurrence with a fixed period). Use of the SI method in conjunction with PTPI represents a major advance over the existing fast methods.

Estimation of the case reporting probability *p*_rep_ is possible using maximum likelihood approaches, including trajectory matching. However, a fast way to obtain a crude estimate of *p*_rep_ is to divide cumulative reported incidence over the time interval [*t*_0_, *t*_*n*_], by the cumulative incidence that is expected from the unforced SIR model (system (1) with *β* ≡ 〈*β*〉, *ν* ≡ *ν*_c_, and *μ* ≡ *μ*_c_) at equilibrium:

$$\begin{array}{c} p_{\text{rep}}\approx \frac{\sum_{k=1}^{n}C_{k}}{\nu_{\text{c}}{\hat{N}}_{0}\left(1-\frac{1}{R_{0}}\right)\left(t_{n}-t_{0}\right)}\;. \end{array}$$ (60)

This approximation can be made in temporal subintervals to obtain a time-varying reporting rate, which would replace the constant *p*_rep_ in Eq (26a). Sensitivity of the SI method to mis-specification of the mean generation interval (*t*_gen_) may be of greater concern, though if the distribution of the incubation period (time from infection to onset of symptoms) is narrow, then *t*_gen_ will be well approximated by the (observable) mean serial interval [44].

Overall, the SI method, in conjunction with PTPI, represents a highly tractable approach to reconstructing susceptibles and *β*(*t*) from infectious disease time series that span decades or centuries. It makes fewer assumptions about the disease and population of interest than the regression-based tSIR method [7, 23] (*i.e*., it does not require an infectious period equal to the observation interval, ignore susceptible mortality, or assume that cumulative incidence approximates cumulative births). Moreover, it is significantly less complex and much less computationally demanding than simulation-based methods of inference, such as iterated filtering [8, 19, 20] and generalized profiling [21, 22].

Even when the observed infectious disease time series is short enough that simulation-based methods are tractable, the approach to transmission rate reconstruction that we promote here can be usefully employed to provide better starting conditions at negligible computational cost.

## Supporting information

S1 Text Text supplement. A .pdf document containing annotated R code, making the results reported here completely reproducible by the reader.
(PDF)

S1 File Source files. A .zip archive containing all of the source files needed to compile S1 Text.
(ZIP)
