# Supplementary material for: Fast estimation of time-varying infectious disease transmission rates
Source: PLoS Comput Biol. 2020 Sep 21;16(9):e1008124. doi: 10.1371/journal.pcbi.1008124 (PMC7549798; doi:10.1371/journal.pcbi.1008124)
Supplement: S1 Text — A .pdf document containing annotated R code, making the results reported here completely reproducible by the reader. (PDF) [file pcbi.1008124.s001.pdf]

Supporting Information

Fast estimation of time-varying infectious disease transmission rates

PLOS Computational Biology

Mikael Jagan, Michelle S. deJonge, Olga Krylova, David J. D. Earn

September 23, 2020

Contents

S0 Preliminaries **1**

S1 Example of $\beta(t)$ estimation using the FC, S, and SI methods **2**
S1.1 Creating a list of parameter values 2
S1.2 Simulating time series data 4
S1.3 Estimating the time-varying transmission rate 5
S1.4 Measuring estimation error 9

S2 Effect of process and observation error **10**

S3 Averaging the raw estimate of $\beta(t)$ **13**

S4 Smoothing the raw estimate of $\beta(t)$ **16**

S5 Sensitivity to data-generating parameters **22**
S5.1 Sensitivity to $\mathcal{R}_0$ and α 23
S5.2 Sensitivity to S_0, I_0, ν_c, μ_c, t_gen, and p_rep 27
S5.3 A note on smoothing 30
S5.3.1 C_k and β_k for $t_\mathrm{gen} = (2^{-1.5}, 1, 2^{1.5}) \cdot 13$ days 30
S5.3.2 $\beta_\mathrm{loess}(t; q^*)$ and $\beta_\mathrm{loess}(t; q_\mathrm{opt})$ for $t_\mathrm{gen} = (2^{-1.5}, 1, 2^{1.5}) \cdot 13$ days 32
S5.3.3 Discussion 35

S6 Sensitivity to error in input parameters **36**
S6.1 A note on smoothing 39

S7 Estimating S_0 via PTPI: Example **39**
S7.1 Truncation step 40
S7.2 Iteration step 43

S8 Estimating S_0 via PTPI: Convergence **47**

S9 Appendix: Choice of discretization in the SI method **50**
S9.1 Nine discretization schemes 50
S9.2 Comparison of RRMSE, bias, and variance 51
S9.2.1 RRMSE 51
S9.2.2 Bias and variance 55

S0 Preliminaries

This supplement to the manuscript is compiled from the source file `S1_Text.Rnw` using R version 4.0.2 (2020-06-22) [1] and these R package versions:

```
##         knitr   tikzDevice   colorRamps RColorBrewer       scales
##          1.29     0.12.3.1          2.3        1.1-2        1.1.1
##       deSolve  adaptivetau
##          1.28        2.2-3
```

Our primary aim here is to make our results entirely reproducible by the reader. Our secondary aim is to make our methods available to potential users. To this end, we introduce our R package **fastbeta**, which implements

- the FC, S, and SI methods for estimating time-varying transmission rates $\beta(t)$ from time series data; and
- peak-to-peak iteration (PTPI) for estimating the initial number of susceptible individuals S_0 from time series data.

All methods are based on the SIR model with time-varying rates of birth, death, and transmission:

$$\frac{\mathrm{d}S}{\mathrm{d}t} = \nu(t)\widehat{N}_0 - \beta(t)SI - \mu(t)S\,, \tag{1a}$$

$$\frac{\mathrm{d}I}{\mathrm{d}t} = \beta(t)SI - \gamma I - \mu(t)I\,, \tag{1b}$$

$$\frac{\mathrm{d}R}{\mathrm{d}t} = \gamma I - \mu(t)R\,, \tag{1c}$$

where $\gamma = 1/t_{\mathrm{gen}}$.

The most recent version of **fastbeta** is located in our GitHub repository and can be installed using `install_github()` from the **remotes** package.

```r
if (!require(remotes)) install.packages("remotes")
remotes::install_github("davidearn/fastbeta")
```

However, readers attempting to compile this document from source must install fastbeta from the `plos` branch of the repository, which houses the version used at the time of this writing.

```r
if (!require(remotes)) install.packages("remotes")
remotes::install_github("davidearn/fastbeta", ref = "plos")
```

For complete compilation instructions, refer to `README.md`.

Here is a list of functions implemented in the `plos` version of fastbeta:

```
library(fastbeta)
ls("package:fastbeta")

##  [1] "compute_rrmse"    "estimate_beta_FC" "estimate_beta_S"
##  [4] "estimate_beta_SI" "get_peak_times"   "make_data"
##  [7] "make_par_list"    "ptpi"             "test_s2dgbeta"
## [10] "test_s2dgpars"    "test_s2inpars"
```

We will introduce these on the fly. More complete documentation can be accessed by running `?fastbeta`.

In the sections to follow, we annotate the R code needed to reproduce results reported in the manuscript. Plotting commands have been suppressed, but are preserved in `S1_Text.Rnw`. Since our work involves millions of simulations, we have retained certain output in the directory `RData/` to significantly reduce compilation time.

S1 Example of $\beta(t)$ estimation using the FC, S, and SI methods

Fig 1 in the manuscript compares the output of the FC, S, and SI methods for a simulated time series of reported incidence. To reproduce Fig 1, we simulate time series data using system (1) with constant vital rates ν_c and μ_c and a seasonally forced transmission rate that includes environmental noise:

$$\beta_\phi(t) = \langle\beta\rangle \left[1 + \alpha \cos\left(\frac{2\pi t}{1\,\text{year}} + \phi(t;\epsilon)\right)\right]. \tag{2}$$

Doing so, we obtain observations of reported incidence at equally spaced time points

$$t_k = t_0 + k\Delta t\,, \qquad k = 0, \ldots, n\,. \tag{3}$$

We then apply each algorithm (FC, S, SI) to reconstruct the seasonally forced transmission rate from the data.

As this simulate-estimate routine is our main investigative approach going forward, we describe each step in detail here (and reproduce Fig 1 in the process), but we do not repeat these details in later sections.

S1.1 Creating a list of parameter values

Simulating a reported incidence time series from system (1) requires a list of values for all data-generating parameters. Our function `make_par_list()` simplifies the task of

creating such a list. It does this by "filling in the blanks" when we want $\mathcal{R}_0$, $\langle\beta\rangle$, N_0, S_0, or I_0 to depend in a complicated way on other parameters' values.

Below, we call `make_par_list()`, defining all arguments explicitly. Except for `dt_weeks`, which is the observation interval Δt in weeks, the values of time and rate parameters must be supplied in units Δt and per unit Δt. In this call to `make_par_list()`, we indicate $\Delta t = 1$ week, $t_0 = 2000$ years, $t_{\text{gen}} = 13$ days, and $\nu_c = \mu_c = 0.04\,\text{year}^{-1}$.

```
## List of parameter values
par_list <- make_par_list(
  dt_weeks  = 1,                      # observation interval
  t0        = 2000 * (365 / 7) * 1, # time of first observation
  prep      = 1,                      # case reporting probability
  trep      = 0,                      # case reporting delay
  hatN0     = 1e06,                   # population size     at 0 years
  N0        = NA,                     # population size     at `t0`
  S0        = NA,                     # number susceptibles at `t0`
  I0        = NA,                     # number infecteds    at `t0`
  nu        = 0.04 * (7 / 365) * 1, # birth rate (relative to `hatN0`)
  mu        = 0.04 * (7 / 365) * 1, # natural mortality rate (per capita)
  tgen      = 13 * (1 / 7) / 1,      # mean generation interval
  Rnaught   = 20,                     # basic reproduction number
  beta_mean = NA,                     # mean of seasonal forcing
  alpha     = 0.08,                   # amplitude of seasonal forcing
  epsilon   = 0                       # s.d. of environmental noise
)
unlist(par_list) # printed as a named vector

##     dt_weeks           t0         prep         trep        hatN0
## 1.000000e+00 1.042857e+05 1.000000e+00 0.000000e+00 1.000000e+06
##           N0           S0           I0           nu           mu
## 1.000000e+06 5.405182e+04 1.318586e+03 7.671233e-04 7.671233e-04
##         tgen      Rnaught    beta_mean        alpha      epsilon
## 1.857143e+00 2.000000e+01 1.078457e-05 8.000000e-02 0.000000e+00
```

`make_par_list()` requires that exactly one of $\mathcal{R}_0$ and $\langle\beta\rangle$ (arguments `Rnaught` and `beta_mean`) is defined in the function call. It sets the undefined parameter internally by enforcing the identity

$$\mathcal{R}_0 = \frac{\nu_c \widehat{N}_0}{\mu_c} \cdot \frac{\langle\beta\rangle}{\gamma + \mu_c}\,. \tag{4}$$

Above, we "omitted" `beta_mean` from the function call (by supplying a value `NA`) and obtained the desired value in the output.

Values for N_0, S_0, and I_0 (arguments `N0`, `S0`, and `I0`) were also set internally. This was done via numerical integration of system (1) with constant vital rates ν_c and μ_c and a seasonally forced transmission rate that *excludes* environmental noise:

$$\beta(t) = \langle \beta \rangle \left[1 + \alpha \cos\left(\frac{2\pi t}{1\,\text{year}} \right) \right] . \tag{5}$$

After integrating between times $t = 0$ years and $t = t_0$ (in this case 2000 years) `make_par_list()` chooses for N_0, S_0 and I_0 the values of $N^* = N(t_0)$, $S^* = S(t_0)$, and $I^* = I(t_0)$. This ensures that the initial state of any simulation using `par_list` is a point very near the attractor of the system being simulated.

In the above call to `make_par_list()`, we indicated the default values of all arguments. In future calls to this function, we will not specify arguments explicitly except for clarity or to request a value different from the default.

S1.2 Simulating time series data

To reproduce the simulation considered in Fig 1, we pass `par_list` to our function `make_data()`, which returns the simulated time series data in a data frame.

```
## Data frame containing time series data
df <- make_data(
  par_list       = par_list,     # parametrization
  n              = 20 * 365 / 7, # time series length: ~20 years
  with_dem_stoch = FALSE         # no demographic stochasticity
)
head(df)

##          t  t_years         beta     beta_phi     N        S        I
## 1 104285.7 2000.000 1.164734e-05 1.164734e-05 1e+06 54052.00 1319.000
## 2 104286.7 2000.019 1.164108e-05 1.164108e-05 1e+06 53910.11 1442.447
## 3 104287.7 2000.038 1.162241e-05 1.162241e-05 1e+06 53692.54 1573.095
## 4 104288.7 2000.058 1.159158e-05 1.159158e-05 1e+06 53398.91 1708.214
## 5 104289.7 2000.077 1.154904e-05 1.154904e-05 1e+06 53031.36 1844.220
## 6 104290.7 2000.096 1.149543e-05 1.149543e-05 1e+06 52594.95 1976.772
##          R         Q         Z    C
## 1 944629.0    0.0000        NA   NA
## 2 944647.4  867.5997  867.5997  868
## 3 944734.4 1811.0116  943.4119  943
## 4 944892.9 2830.6905 1019.6789 1020
## 5 945124.4 3924.5379 1093.8474 1094
## 6 945428.3 5087.5473 1163.0094 1163
```

The output contains the following information:

`t`	$\frac{t_k}{\Delta t}$	Time in units of the observation interval Δt. Equal to $\frac{t_0}{\Delta t} + (0, 1, \ldots, n)$.
`t_years`	t_k	Time in years. Equal to $t_0 + (0, 1, \ldots, n)\Delta t$.
`beta`	$\beta(t_k)\Delta t$	Seasonally forced transmission rate without environmental noise (Eq (5)), expressed per unit Δt per susceptible per infected.
`beta_phi`	$\beta_\phi(t_k)\Delta t$	Seasonally forced transmission rate with environmental noise (Eq (2)), expressed per unit Δt per susceptible per infected.
`N`	$N(t_k)$	Population size.
`S`	$S(t_k)$	Number of susceptible individuals.
`I`	$I(t_k)$	Number of infected individuals.
`R`	$R(t_k)$	Number of removed individuals.
`Q`	$Q(t_k)$	Cumulative incidence.
`Z`	$Z(t_k)$	Incidence.
`C`	$C(t_k)$	Reported incidence.

In this example, there is no environmental noise, simply because `par_list` specified `epsilon = 0`. Hence `beta` and `beta_phi` are identical in the returned data frame.

S1.3 Estimating the time-varying transmission rate

We apply the FC, S, and SI methods to estimate incidence Z, susceptibles S, infecteds I, and the seasonally forced transmission rate β from reported incidence and vital data. We have implemented these methods in our functions `estimate_beta_FC()`, `estimate_beta_S()`, and `estimate_beta_SI()`. We will refer to these collectively as `estimate_beta()`, but note that there is no function by that name.

The first argument of `estimate_beta()` expects a data frame `df` with columns `t`, `C`, `B`, and (for the S and SI methods) `mu`. These specify equally spaced observation times t_k (Eq (3)) in units of the observation interval Δt (*i.e.*, $t_k/\Delta t$) and, at those times, reported incidence C_k, observed births B_k, and the observed *per capita* natural mortality rate μ_k expressed per unit Δt.

The second argument expects a list `par_list` with elements `prep`, `trep`, `tgen`, `S0`, and (for the SI method) `I0`. These specify values for input parameters p_{rep}, t_{rep}, t_{gen}, S_0, and I_0.

Here, we supply the data frame `df` obtained earlier using `make_data()`. Note that this data frame does *not* have columns `B` and `mu`. When these columns are missing in the function call, `estimate_beta()` creates mock (constant) time series as follows: (i) it looks in `par_list` for additional elements `hatN0`, `nu`, and `mu`, specifying a population size $\widehat{N}_0$ and constant vital rates ν'_{c} and μ'_{c}, then (ii) it sets $B_k = \nu'_{\mathrm{c}}\widehat{N}_0\Delta t$ and $\mu_k = \mu'_{\mathrm{c}}$ for all k.

In practice, the argument `par_list` contains the user's potentially incorrect estimates of the input parameters, such as the initial number of susceptibles individuals S_0. For this example, there is no input error: we assign each input parameter its true (data-generating) value. That is, the `par_list` that we pass to `estimate_beta()` is precisely the `par_list` that we passed to `make_data()` earlier.

```
## List of functions
estimate_beta <- list(
  FC = estimate_beta_FC,
  S  = estimate_beta_S,
  SI = estimate_beta_SI
)

## List of returned data frames
df_est <- lapply(estimate_beta, function(f) f(df, par_list))
lapply(df_est, head, n = 10)

## $FC
##            t    C    Z Z_agg        B    B_agg        S    I         beta
## 1  104285.7   NA   NA    NA 767.1233       NA 54051.82   NA           NA
## 2  104286.7  868  868    NA 767.1233       NA       NA   NA           NA
## 3  104287.7  943  943  1811 767.1233 1534.247 53775.06 1811 1.085364e-05
## 4  104288.7 1020 1020    NA 767.1233       NA       NA   NA           NA
## 5  104289.7 1094 1094  2114 767.1233 1534.247 53195.31 2114 1.061314e-05
## 6  104290.7 1163 1163    NA 767.1233       NA       NA   NA           NA
## 7  104291.7 1224 1224  2387 767.1233 1534.247 52342.56 2387 1.034483e-05
## 8  104292.7 1274 1274    NA 767.1233       NA       NA   NA           NA
## 9  104293.7 1311 1311  2585 767.1233 1534.247 51291.80 2585 1.006868e-05
## 10 104294.7 1332 1332    NA 767.1233       NA       NA   NA           NA
##
## $S
##            t    C    Z        B           mu        S        I
## 1  104285.7   NA   NA 767.1233 0.0007671233 54051.82       NA
## 2  104286.7  868  868 767.1233 0.0007671233 53909.48       NA
## 3  104287.7  943  943 767.1233 0.0007671233 53692.25 1609.707
## 4  104288.7 1020 1020 767.1233 0.0007671233 53398.18 1748.794
```

```
## 5  104289.7 1094 1094 767.1233 0.0007671233 53030.34 1891.591
## 6  104290.7 1163 1163 767.1233 0.0007671233 52593.78 2028.824
## 7  104291.7 1224 1224 767.1233 0.0007671233 52096.56 2156.784
## 8  104292.7 1274 1274 767.1233 0.0007671233 51549.72 2269.909
## 9  104293.7 1311 1311 767.1233 0.0007671233 50966.30 2362.634
## 10 104294.7 1332 1332 767.1233 0.0007671233 50362.32 2431.251
##            beta
## 1            NA
## 2            NA
## 3  1.180163e-05
## 4  1.171527e-05
## 5  1.159386e-05
## 6  1.147104e-05
## 7  1.133845e-05
## 8  1.120387e-05
## 9  1.106177e-05
## 10 1.092750e-05
##
## $SI
##           t    C    Z        B           mu        S        I
## 1  104285.7   NA   NA 767.1233 0.0007671233 54051.82 1318.586
## 2  104286.7  868  868 767.1233 0.0007671233 53909.53 1442.230
## 3  104287.7  943  943 767.1233 0.0007671233 53692.38 1572.434
## 4  104288.7 1020 1020 767.1233 0.0007671233 53398.43 1707.986
## 5  104289.7 1094 1094 767.1233 0.0007671233 53030.73 1844.252
## 6  104290.7 1163 1163 767.1233 0.0007671233 52594.34 1976.990
## 7  104291.7 1224 1224 767.1233 0.0007671233 52097.31 2101.398
## 8  104292.7 1274 1274 767.1233 0.0007671233 51550.68 2212.350
## 9  104293.7 1311 1311 767.1233 0.0007671233 50967.48 2305.321
## 10 104294.7 1332 1332 767.1233 0.0007671233 50363.73 2375.346
##            beta
## 1            NA
## 2  1.164631e-05
## 3  1.162533e-05
## 4  1.158944e-05
## 5  1.153862e-05
## 6  1.147834e-05
## 7  1.140877e-05
## 8  1.133293e-05
## 9  1.124715e-05
## 10 1.115929e-05
```

The output contains the following information:

`t`	$\frac{t_k}{\Delta t}$	Time in units of the observation interval Δt. Equal to $\frac{t_0}{\Delta t} + (0, 1, \ldots, n)$. Identical to input `df$t`.
`C`	C_k	Reported incidence. Identical to input `df$C`, except that missing values and zeros (treated like missing values) have been imputed by linear interpolation.
`B`	B_k	Observed births. Identical to input `df$B` if supplied. Otherwise, every element is `with(par_list, hatN0 * nu * 1)`.
`mu`	$\mu_k \Delta t$	Observed *per capita* natural mortality rate, expressed per unit Δt. Identical to input `df$mu` if supplied. Otherwise, every element is `with(par_list, mu)`.
`Z`	Z_k	Estimate of incidence $Z(t_k)$.
`S`	S_k	Estimate of susceptibles $S(t_k)$.
`I`	I_k	Estimate of infecteds $I(t_k)$.
`beta`	$\beta_k \Delta t$	Raw estimate of the time-varying transmission rate $\beta(t_k)$ expressed per unit Δt per susceptible per infected.

Note that the FC method's output has missing values in alternating rows. This is not a mistake: the FC method aggregates incidence and births over the mean generation interval (roughly 2 weeks in this example), instead of the observation interval (1 week). As a result, estimation of $S(t_k)$ and $\beta(t_k)$ is only possible at every other observation time. Aggregation is not a requirement of the S and SI methods.

Be warned that `estimate_beta()` returns *raw* estimates of $\beta(t)$. Smoothing the β_k time series by fitting a loess curve $\beta_{\text{loess}}(t; q)$ is recommended in the event that it displays unwanted noise. Determining what degree of smoothing is optimal (*i.e.*, choosing a good value for the loess smoothing parameter q) is non-trivial, and therefore not undertaken by `estimate_beta()`. We discuss this issue in more detail in §S4.

Plotting the S_k and β_k time series returned by `estimate_beta()`, we reproduce Fig 1. Note that we are simply plotting (with a scaling) `df_est$FC$S`, `df_est$S$S`, and `df_est$SI$S`, and separately `df_est$FC$beta`, `df_est$S$beta`, and `df_est$SI$beta`.

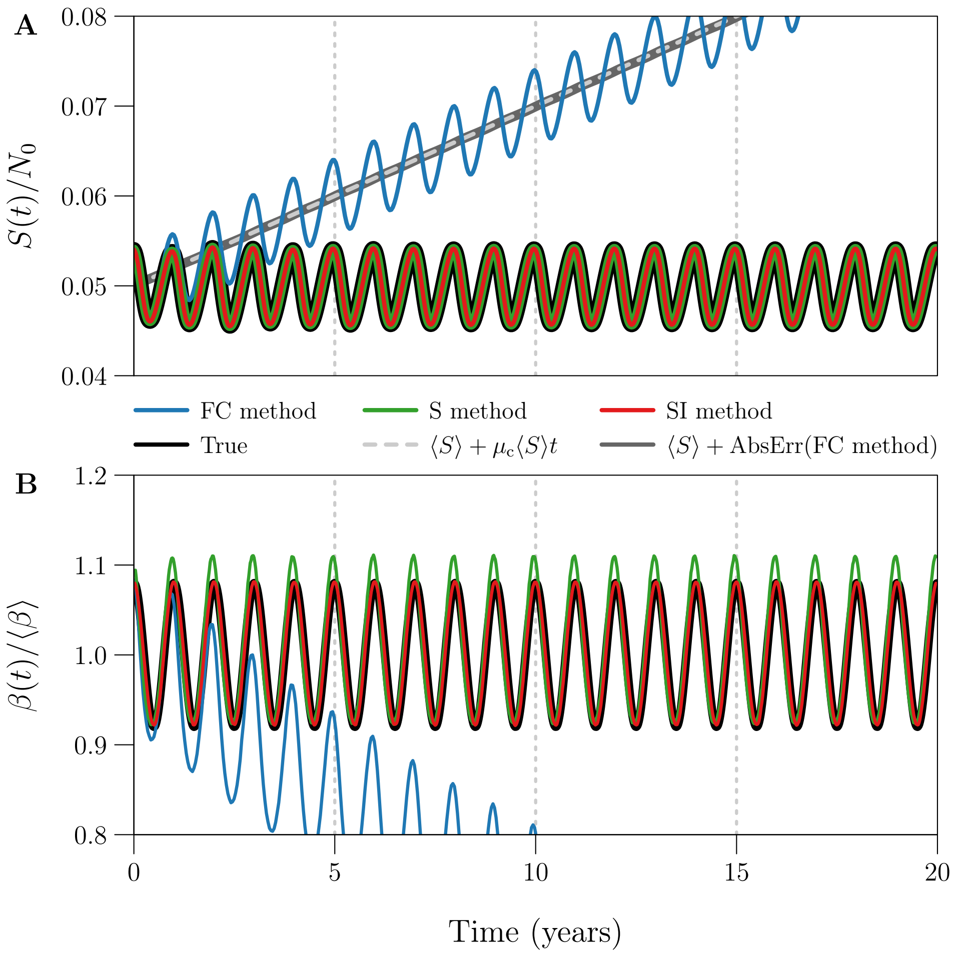

Fig 1. Example of $S(t)$ and $\beta(t)$ estimation using the FC, S, and SI methods.

S1.4 Measuring estimation error

In the manuscript, we report the relative root mean square error (RRMSE) in each β_k time series. We compute RRMSE using `compute_rrmse()`, which takes as arguments the two vectors we wish to compare, with the estimate passed second.

```
sapply(df_est, function(x) compute_rrmse(df$beta, x$beta))

##          FC           S          SI
## 0.335487234 0.024010753 0.002139994
```

S2 Effect of process and observation error

Fig 1 in the manuscript shows the output of the S and SI methods for idealized reported incidence data. Those data were simulated deterministically, *i.e.*,

- without environmental stochasticity [ES] (`par_list` with `epsilon = 0` passed to `make_data()`),
- without demographic stochasticity [DS] (`with_dem_stoch = FALSE` passed to `make_data()`), and
- without observation error [OE] (`par_list` with `prep = 1` passed to `make_data()`).

In Fig 2, we consider the effect of adding ES, DS, and OE in turn to the original deterministic simulation, on the β_k time series generated by the S and SI methods. We produce these four simulations as follows:

```
## List of lists of parameter values
par_list <- list(
  xxxxxx = make_par_list(epsilon = 0, prep = 1),     # deterministic
  esxxxx = make_par_list(epsilon = 0.5, prep = 1),   # ES
  esdsxx = make_par_list(epsilon = 0.5, prep = 1),   # ES+DE
  esdsoe = make_par_list(epsilon = 0.5, prep = 0.25) # ES+DE+OE
)

## List of data frames containing time series data
df <- mapply(make_data,
  par_list = par_list,
  n = 3 * 365 / 7,
  with_dem_stoch = c(FALSE, FALSE, TRUE, TRUE),
  seed = 1305,
  SIMPLIFY = FALSE
)
names(df)

## [1] "xxxxxx" "esxxxx" "esdsxx" "esdsoe"

names(df$esdsoe)

##  [1] "t"        "t_years"  "beta"     "beta_phi" "N"        "S"
##  [7] "I"        "R"        "Q"        "Z"        "C"
```

Here, `par_list` is a *list of lists*, containing the parameter values desired for each simulation, and `df` is a list of data frames, containing the corresponding simulated data.

The next code chunk applies the S and SI methods, without input error, to each simulated reported incidence time series.

```
## List of lists of data frames containing estimation output
df_est <- list(
  ## List of data frames returned by S method
  S  = mapply(estimate_beta_S,
    df = df,
    par_list = par_list,
    SIMPLIFY = FALSE
  ),
  ## List of data frames returned by SI method
  SI = mapply(estimate_beta_SI,
    df = df,
    par_list = par_list,
    SIMPLIFY = FALSE
  )
)
names(df_est)

## [1] "S"  "SI"

names(df_est$SI)

## [1] "xxxxxx" "esxxxx" "esdsxx" "esdsoe"

names(df_est$SI$esdsoe)

## [1] "t"    "C"    "Z"    "B"    "mu"   "S"    "I"    "beta"
```

Hence each element of `df_est` contains the output of one of the S and SI methods for all 4 reported incidence time series. Plotting the resulting 8 Z_k, I_k, and β_k time series (estimates of incidence, prevalence, and the seasonally forced transmission rate) yields Fig 2.

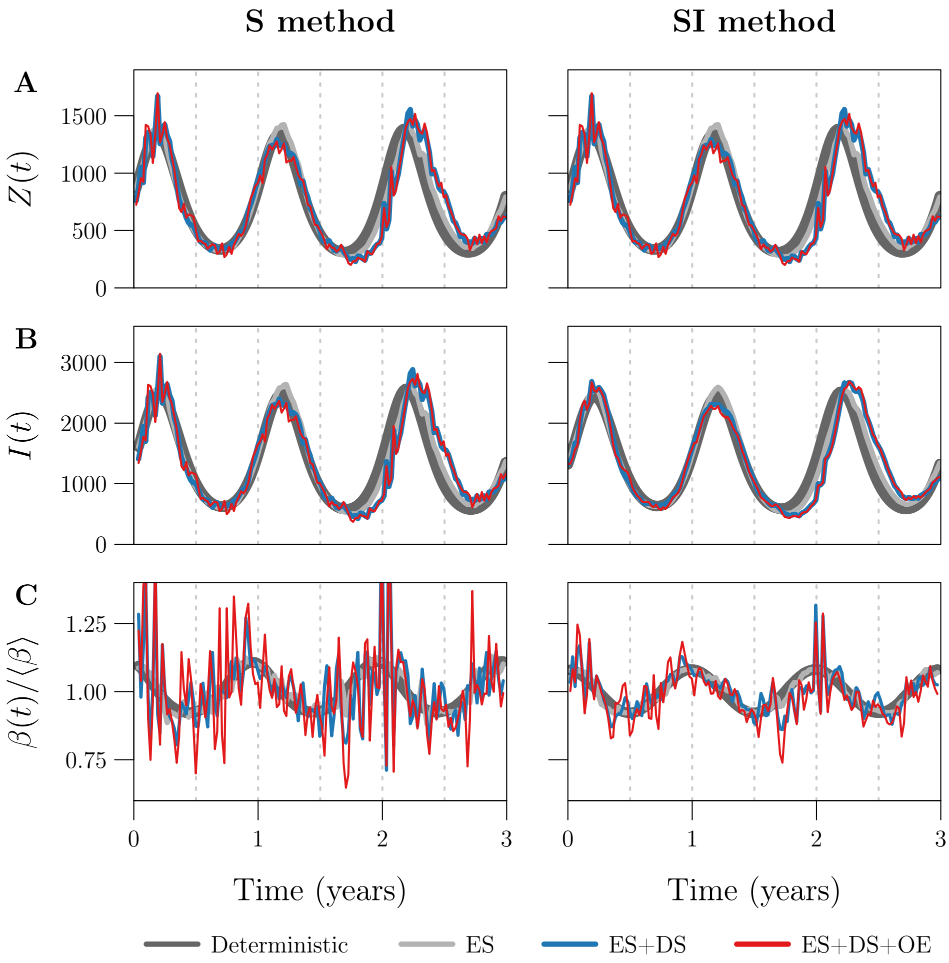

Fig 2. Effects of process and observation error on the S and SI methods.

131 We obtain the RRMSE in each β_k time series with the following line of code.

```
lapply(df_est, function(x) {
  mapply(function(y, z) compute_rrmse(y$beta, z$beta), y = df, z = x)
})

## $S
##     xxxxxx     esxxxx     esdsxx     esdsoe
## 0.02393485 0.03352897 0.12261568 0.15458400
##
## $SI
##      xxxxxx      esxxxx      esdsxx      esdsoe
## 0.002145247 0.015053954 0.053576843 0.070091436
```

S3 Averaging the raw estimate of $\beta(t)$

Fig 3 in the manuscript considers the seasonally forced $\beta(t)$ (Eq (5)) and three estimates, each spanning 1000 years. It overlays the 1000 1-year cycles embedded in each estimate and plots their 1-year average. To reproduce Fig 3, we simulate 1000 years of weekly reported incidence, including in the simulation environmental noise in transmission ($\epsilon = 0.5$), demographic stochasticity, and random under-reporting of cases ($p_{\text{rep}} = 0.25$).

```
## List of parameter values
par_list <- make_par_list(epsilon = 0.5, prep = 0.25)

## Data frame containing time series data
df <- make_data(
  par_list       = par_list,
  n              = 1000 * 365 / 7 + 1,
  with_dem_stoch = TRUE,
  seed           = 1217
)
```

We estimate the seasonally forced $\beta(t)$ using the S and SI methods, without input error.

```
## List of functions
estimate_beta <- list(
  S  = estimate_beta_S,
  SI = estimate_beta_SI
)

## List of returned data frames. Column `beta` in `df_est[[i]]`
## stores the raw estimate generated by `estimate_beta[[i]]`.
df_est <- lapply(estimate_beta, function(f) f(df, par_list))
```

The raw time series estimates β_k contain spurious noise due to process and observation error. Hence, for comparison, we fit a loess curve $\beta_{\text{loess}}(t; q)$ to the time series returned by the SI method, where q is the loess smoothing parameter indicating (roughly) the number of nearest neighbours weighted in local regression. It turns out that $q = 53$ is a good choice for this parameter in this example (see §S4). To do the fitting, we call `loess()` with argument `span` indicating q as a proportion of time series length. Additional arguments are fully explained in the documentation, accessible by running `?loess` and `?loess.control`.

```r
## Object of class `loess` defining the fitted loess curve
SI_loess <- stats::loess(
  formula   = beta ~ t,
  data      = df_est$SI,
  span      = 53 / nrow(df_est$SI),
  degree    = 2,
  na.action = "na.exclude",
  control   = loess.control(surface = "direct")
)
```

We will calculate the average 1-year cycle in the linear interpolant $\beta_{\text{int}}(t)$ of β_k (S and SI methods), and in the loess curve $\beta_{\text{loess}}(t;q)$ fit to the same time series (SI method only).

```r
## List of interpolants. `fits[[i]]` is the interpolant
## of column `beta` in `df_est[[i]]`.
fits <- lapply(df_est, function(x) {
  approxfun(x$t, x$beta, method = "linear", rule = 1)
})

## Appending the `loess` object from earlier
fits$SI_loess <- SI_loess
```

Before proceeding, we should verify that the β_k time series contain 1000 1-year cycles.

```r
## First and last time points
t0 <- df_est$SI$t[1]
tn <- df_est$SI$t[nrow(df_est$SI)]

## 1-year period in units of the observation interval
period <- with(par_list, (365 / 7) / dt_weeks)

## Number of cycles
m <- floor((tn - t0) / period)
m

## [1] 1000
```

We must also specify the true 1-year cycle that each average 1-year cycle should estimate. We specify the true cycle with a reference time indicating the initial phase. For simplicity, we consider the cycle between times `t0` and `t0 + period` to be the true cycle. Phases of this cycle are specified by times `s` between 0 and `period`. To estimate the value of the true cycle at phase `s`, we evaluate each fit in `fits` (2 linear interpolants and 1 loess

curve) at times `t0 + s + (0:(m-1))* period` and average the resulting `m` values. `get_phase_average()` computes this estimate for any `s`, for a given fit `f`.

```
get_phase_average <- function(s, f) {
  times <- t0 + (s %% period) + (0:(m-1)) * period
  x <- if (inherits(f, "loess")) predict(f, times) else f(times)
  mean(x, na.rm = TRUE)
}
```

Note that, whereas linear interpolants are just functions that we can evaluate at desired times, loess objects must be passed to `predict()` to obtain fitted values. Note also that the modulo operation `s %% period` makes `get_phase_average()` a periodic function of `s`.

We construct the average 1-year cycle in each fit in `fits` by applying `get_phase_average()` on a desired grid of `s` values.

```
s_grid <- seq(0, period, length.out = 150)
average_one_year <- data.frame(
  s_grid,
  lapply(fits, function(f) sapply(s_grid, get_phase_average, f = f))
)
head(average_one_year)

##      s_grid            S           SI     SI_loess
## 1 0.0000000 1.193014e-05 1.153366e-05 1.148309e-05
## 2 0.3499521 1.194339e-05 1.154372e-05 1.148619e-05
## 3 0.6999041 1.193943e-05 1.155337e-05 1.148787e-05
## 4 1.0498562 1.191978e-05 1.155581e-05 1.148843e-05
## 5 1.3998082 1.190497e-05 1.155318e-05 1.148760e-05
## 6 1.7497603 1.189256e-05 1.154800e-05 1.148552e-05
```

We reproduce Fig 3 by plotting, for each 1000-year estimate of $\beta(t)$, the 1000 embedded 1-year cycles and their 1-year average, all on the same 1-year axis.

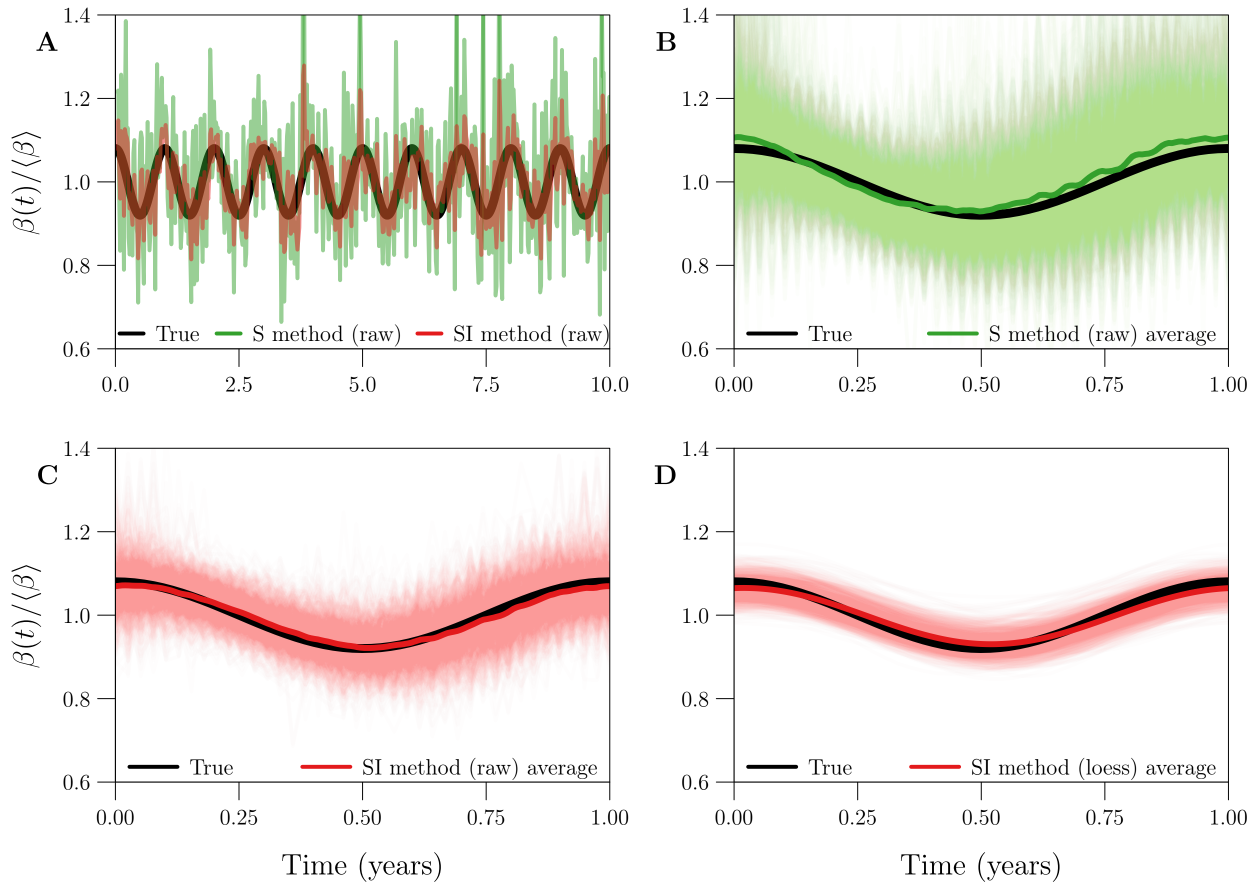

Fig 3. Bias and variance in 1-year cycles embedded in three estimates of a seasonally forced $\beta(t)$, in the absence of input error.

S4 Smoothing the raw estimate of $\beta(t)$

Figs 2C, 3B, and 3C demonstrate that, in the absence of input error, much of the error in raw estimates of $\beta(t)$ is attributable to noise in the time series. The S and SI methods produce the correct temporal pattern, but it is distorted by noise. Comparison of Figs 3C and 3D shows that fitting a smooth loess curve $\beta_{\text{loess}}(t; q)$ to the raw time series estimate β_k can lead to substantial improvement in both accuracy and interpretability.

In practice, we would like to choose the value q_{opt} of the loess smoothing parameter q that minimizes the error in $\beta_{\text{loess}}(t; q)$ relative to $\beta(t)$. However, we cannot calculate (and therefore cannot minimize) the error in $\beta_{\text{loess}}(t; q)$ when $\beta(t)$ is not known. In this situation, we can still estimate q_{opt} using statistical methods (most notably time series cross-validation [2]) or by direct inspection of $\beta_{\text{loess}}(t; q)$ for each value of q on a grid.

In our simulated data setting, we do know $\beta(t)$ and can therefore determine q_{opt} exactly. In this setting, it is instructive to quantify the reduction in error that is achieved

when the optimal loess estimate $\beta_{\text{loess}}(t; q_{\text{opt}})$ is chosen over the raw time series estimate β_k. Fig 4 in the manuscript addresses this issue.

To reproduce Fig 4, we consider 41 values of the case reporting probability p_{rep} and fix all other data-generating parameters. (Smaller values of p_{rep} generate noisier reported incidence time series, resulting in noisier β_k.) Using each value of p_{rep}, we perform 100 simulations of reported incidence.

```
prep <- 10^seq(-2, 0, length.out = 41)
par_list <- make_par_list(epsilon = 0.5)
nsim <- 100
q <- 10:110
```

For each of these 41×100 simulations, we carry out the following steps. We estimate the seasonally forced $\beta(t)$ (Eq (5)) from the simulated reported incidence time series, without input error. (The underlying $\beta(t)$ was the same across all simulations.) For each q between 10 and 110, we fit a loess curve $\beta_{\text{loess}}(t; q)$ to the raw time series estimate β_k. Finally, we record

$$\begin{aligned}
\text{RRMSE}_{\text{raw}} &= \text{RRMSE in } \{(t_k, \beta_k)\}_{k=0}^{n}\,, \\
\text{RRMSE}_{\text{loess,min}} &= \min_{q \in \{10,\ldots,110\}} \left[\text{RRMSE in } \{(t_k, \beta_{\text{loess}}(t_k; q))\}_{k=0}^{n}\right], \\
q_{\text{opt}} &= \operatorname*{arg\,min}_{q \in \{10,\ldots,110\}} \left[\text{RRMSE in } \{(t_k, \beta_{\text{loess}}(t_k; q))\}_{k=0}^{n}\right].
\end{aligned}$$

Hence, for each value of p_{rep}, we obtain 100 values for each of $\text{RRMSE}_{\text{raw}}$, $\text{RRMSE}_{\text{loess,min}}$, and q_{opt}. We can preallocate space for this output.

```
out <- array(NA,
  dim      = c(length(prep), nsim, 3, 2),
  dimnames = list(NULL, NULL,
    c("rrmse_raw", "rrmse_loess_min", "qopt"),
    c("S", "SI")
  )
)
```

The fourth dimension of the output array enables our comparison of the distributions of $\text{RRMSE}_{\text{raw}}$, $\text{RRMSE}_{\text{loess,min}}$, and q_{opt} for different methods of $\beta(t)$ estimation—in this case, the S and SI methods. Our expectation based on Figs 2C, 3B, and 3C is that the S method requires more smoothing.

The next code chunk does all of the hard work. Simulations are saved in the directory `RData/loess/`. Main results are saved in the file `RData/loess.RData`.

```r
for (i in seq_along(prep)) {

  ## Update `par_list` with current value of `prep`
  par_list$prep <- prep[i]

  ## Create a directory for this loop's `.RData`
  dirname <- paste0(
    "RData/loess/",
    ## log10 current value of `prep`
    "prep_log10v-", sprintf("%+05.0f", log(prep[i], 10) * 1000), "/"
  )
  if (!dir.exists(dirname)) {
    dir.create(dirname, recursive = TRUE)
  }

  for (j in seq_len(nsim)) {

    message(
      "`prep` value ", i, " of ", length(prep), ", ",
      "sim ", j, " of ", nsim
    )

    ## File name for simulation
    filename <- paste0(dirname, "sim", sprintf("%04.0f", j), ".RData")

    ## Simulate reported incidence data, if you haven't already
    if (file.exists(filename)) {
      load(filename)
    } else {
      df <- make_data(
        par_list       = par_list,
        n              = 20 * 365 / 7,
        with_dem_stoch = TRUE,
        seed           = j
      )
      save(df, file = filename)
    }

    ## Estimate the seasonally forced transmission rate from
    ## reported incidence
    estimate_beta <- list(
```

```r
    S  = estimate_beta_S,
    SI = estimate_beta_SI
  )
  df_est <- lapply(estimate_beta, function(f) f(df, par_list))

  ## Compute the error in each raw estimate
  out[i, j, "rrmse_raw", ]  <- sapply(df_est, function(x) {
    compute_rrmse(df$beta, x$beta)
  })

  ## Preallocate memory for the error in each loess estimate
  ## (one for each value of `q`, for each raw estimate)
  rrmse_after_loess  <- array(NA,
    dim      = c(length(q), 2),
    dimnames = list(NULL, c("S", "SI"))
  )

  ## Standardize missing values in the raw estimates. `loess()`
  ## handles `NA` but complains about `NaN` and `Inf`.
  df_est <- lapply(df_est, function(x) {
    x$beta[!is.finite(x$beta)] <- NA
    x
  })

  for (k in seq_along(q)) {

    ## Fit a smooth loess curve to each raw estimate
    loess_fit <- lapply(df_est, function(x) {
      stats::loess(
        formula   = beta ~ t,
        data      = x,
        span      = q[k] / nrow(x),
        degree    = 2,
        na.action = "na.exclude",
        control   = loess.control(
          surface    = "direct",
          statistics = "none"
        )
      )
    })
```

```r
      ## Compute the error in each loess estimate
      rrmse_after_loess[k, ] <- sapply(loess_fit, function(x) {
        compute_rrmse(df$beta, predict(x))
      })

    }

    ## Find the minimum error over all loess estimates.
    ## Also retrieve the value of `q` that minimized error.
    out[i, j, "rrmse_loess_min", ] <- apply(rrmse_after_loess, 2, min)
    out[i, j, "qopt", ] <- apply(rrmse_after_loess, 2, function(x) {
      q[which.min(x)]
    })

  }

}


attr(out, "arg_list") <- list(
  prep     = prep,
  par_list = par_list,
  q        = q
)
save(out, file = "RData/loess.RData")
```

For each value of p_{rep}, we desire the median and 5th and 95th percentiles of $\text{RRMSE}_{\text{raw}}$, $\text{RRMSE}_{\text{loess,min}}$, and q_{opt}.

```r
pct <- apply(out, c(1, 3, 4), quantile, probs = c(0.05, 0.5, 0.95))
```

Plotting these as functions of p_{rep}, we reproduce Fig 3.

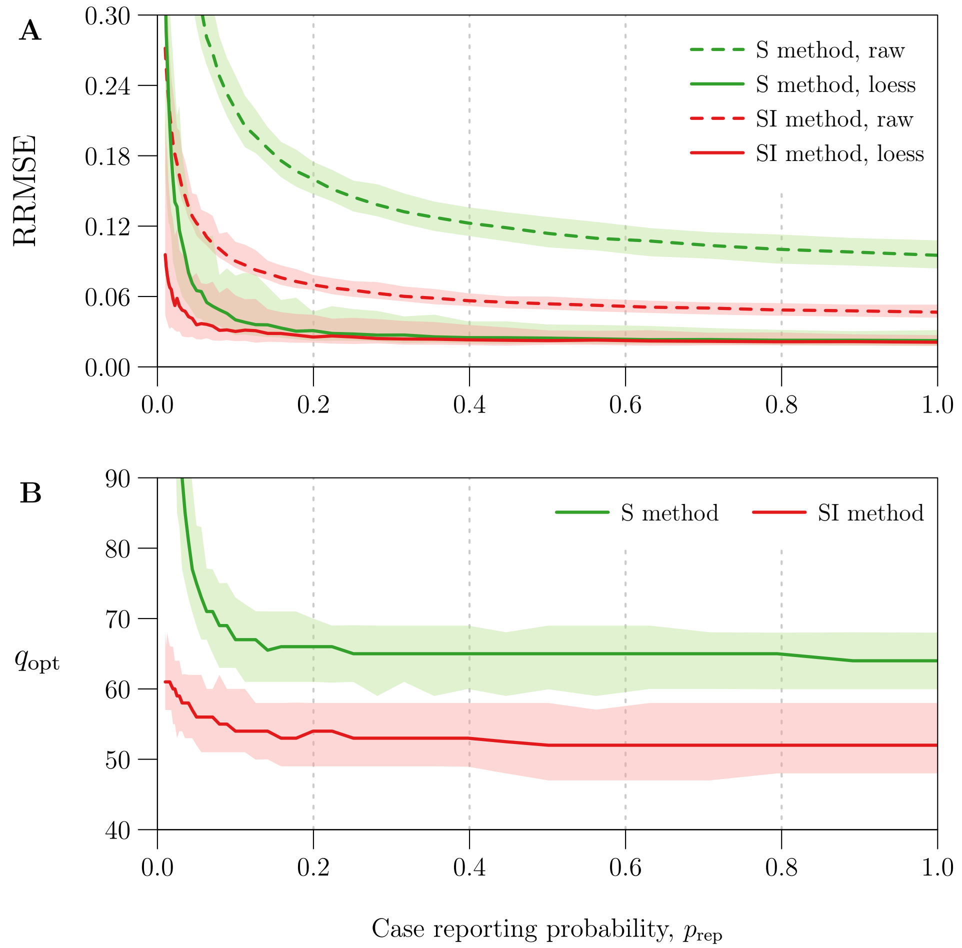

Fig 4. Reduction in $\beta(t)$ estimation error with optimal loess smoothing.

In the manuscript, we report the minimum percentage reduction in RRMSE across all simulations. Borrowing our earlier notation, this is the minimum value of

$$100 \times \left(1 - \frac{\text{RRMSE}_{\text{loess,min}}}{\text{RRMSE}_{\text{raw}}}\right) . \tag{6}$$

```
apply(out, 4, function(x) {
  min(100 * (1 - x[, , "rrmse_loess_min"] / x[, , "rrmse_raw"]), na.rm = TRUE)
})

##        S       SI
## 46.38507 17.47202
```

We also report the median value of q_{opt} across all simulations for which $p_{\text{rep}} \geq 0.1$.

```r
qstar <- apply(out[prep >= 0.1, , "qopt", ], 3, quantile,
  probs = 0.5,
  names = FALSE
)
qstar

##  S SI
## 65 53
```

In our remaining analysis, we set $q = q^*$ when smoothing any β_k time series, taking

$$q^* = \begin{cases} 65 & \text{with the S method,} \\ 53 & \text{with the SI method.} \end{cases} \tag{7}$$

For a given time series, this setting may not be optimal ($q^* \neq q_{\text{opt}}$), but can be justified, as we explain in the sections to follow.

S5 Sensitivity to data-generating parameters

Error in estimates of the seasonally forced $\beta(t)$ (Eq (5)) from simulated reported incidence data is a function of data-generating parameters, given by

$$\boldsymbol{\theta} = (\langle\beta\rangle,\, \alpha,\, \epsilon,\, \widehat{N}_0,\, S_0,\, I_0,\, \nu_{\text{c}},\, \mu_{\text{c}},\, t_{\text{gen}},\, p_{\text{rep}},\, t_{\text{rep}},\, t_0,\, \Delta t,\, n)\,. \tag{8}$$

In order to measure the sensitivity of $\beta(t)$ estimation error to $\boldsymbol{\theta}$, we must define grids of parameter values. For this task, it is helpful to associate with each parameter a reference value:

$$\begin{array}{r|l} \langle\beta\rangle & \beta^* \\ \alpha & 0.08 \\ \epsilon & 0.5 \end{array} \qquad \begin{array}{r|l} \widehat{N}_0 & 10^6 \\ S_0 & S^* \\ I_0 & I^* \end{array} \qquad \begin{array}{r|l} \nu_{\text{c}} & 0.04\ \text{year}^{-1} \\ \mu_{\text{c}} & 0.04\ \text{year}^{-1} \\ t_{\text{gen}} & 13\ \text{days} \end{array} \qquad \begin{array}{r|l} t_0 & 2000\ \text{years} \\ \Delta t & 1\ \text{week} \\ n & 1042 \\ p_{\text{rep}} & p_{\text{rep}}^* \\ t_{\text{rep}} & t_{\text{rep}}^* \end{array} \tag{9}$$

Here, S^* and I^* are the values of S and I at a point very near the attractor of system (1) with constant vital rates ν_{c} and μ_{c} and a seasonally forced transmission rate (Eq (5)). It follows that S^* and I^* vary with parameters of the system (specifically, $\langle\beta\rangle$, α, ν_{c}, μ_{c}, and t_{gen}). See §S1.1 for details on how S^* and I^* are obtained using `make_par_list()` given values for other parameters.

β^* is the value of $\langle\beta\rangle$ that satisfies Eq (4) with $\mathcal{R}_0 = 20$, $\widehat{N}_0 = 10^6$, $\nu_{\text{c}} = \mu_{\text{c}} = 0.04\,\text{year}^{-1}$, and $t_{\text{gen}} = \gamma^{-1} = 13$ days:

$$\begin{aligned} \beta^* &= 20 \cdot \frac{0.04\,\text{year}^{-1}}{10^6 \cdot 0.04\,\text{year}^{-1}} \cdot \left(\tfrac{1}{13}\,\text{day}^{-1} + 0.04\,\text{year}^{-1}\right) \\ &\approx 5.6234 \times 10^{-4}\,\text{year}^{-1}\,. \end{aligned} \tag{10}$$

Finally,

$$p^*_{\text{rep}} = \begin{cases} 1 & \text{for analysis without OE} \\ 0.25 & \text{for analysis with OE} \end{cases} \qquad t^*_{\text{rep}} = \begin{cases} 0 \text{ weeks} & \text{for analysis without OE} \\ 2 \text{ weeks} & \text{for analysis with OE} \end{cases} \tag{11}$$

where an "analysis with OE" is one in which we desire simulations of reported incidence with observation error.

S5.1 Sensitivity to $\mathcal{R}_0$ and α

Fig 5 in the manuscript describes how $\beta(t)$ estimation error depends on features of $\beta(t)$ itself. For the seasonally forced $\beta(t)$ (Eq (5)), these features are the mean $\langle\beta\rangle$ and amplitude α. Fig 5 casts error as a function of $\mathcal{R}_0$ and α, rather than $\langle\beta\rangle$ and α, but this formulation is equivalent, because $\mathcal{R}_0$ is proportional to $\langle\beta\rangle$ (Eq (4)). It is also more interpretable: unlike $\langle\beta\rangle$, $\mathcal{R}_0$ has a natural epidemiological meaning and is dimensionless (its numerical value does not depend on the chosen units of time).

To reproduce Fig 5, we set all data-generating parameters other than $\langle\beta\rangle$ and α equal to their reference value in (9). We consider the grid of $(\mathcal{R}_0, \alpha)$ pairs with levels $\mathcal{R}_0 = 2, 3, \ldots, 32$ and $\alpha = 0, 0.01, \ldots, 0.2$—defining $\langle\beta\rangle$ for each $\mathcal{R}_0$ using Eq (4)—and simulate 1000 reported incidence time series using each of these 31×21 parametrizations.

```
Rnaught <- seq(2, 32, by = 1)
alpha <- seq(0, 0.2, by = 0.01)
nsim <- 1000
```

We estimate $\beta(t)$ from each simulated reported incidence time series, without input error, fit a loess curve $\beta_{\text{loess}}(t; q)$ to the raw estimate β_k, and record the RRMSE in $\beta_{\text{loess}}(t_k; q)$. For comparison, this is done using both the S and SI methods. We fix $q = q^*$ (Eq (7)) independently of the β_k time series being smoothed. (See §S5.3 for discussion of the consequences of using fixed q in this analysis.)

This algorithm is implemented in our function `test_s2dgbeta()` ("sensitivity to data-generating $\beta(t)$"), which takes as arguments

- `par_list_ref`, a list containing the reference values of all data-generating parameters;
- `Rnaught` and `alpha`, numeric vectors specifying the desired grid of $(\mathcal{R}_0, \alpha)$ pairs;
- `with_dem_stoch`, a logical scalar indicating whether simulations should account for demographic stochasticity;
- `nsim`, the number of simulations to perform using each $(\mathcal{R}_0, \alpha)$ pair;

- `loess_par`, a numeric vector of length 2 specifying the value of the loess smoothing parameter q used when fitting loess curves to raw transmission rate estimates β_k. `loess_par[1]` is used with the S method. `loess_par[2]` is used with the SI method.

`s2dgbeta()` returns a 4-dimensional array, whose `[i, j, k, m]`th entry is the RRMSE in an estimate of $\beta(t)$ (S method if `m = 1`, SI method if `m = 2`) from simulation k of `nsim` using the (i, j)th $(\mathcal{R}_0, \alpha)$ grid point.

First, we consider simulations with environmental stochasticity ($\epsilon = 0.5$), without demographic stochasticity, and without observation error ($p_{\text{rep}} = 1$, $t_{\text{rep}} = 0$).

```
rrmse_esxxxx <- test_s2dgbeta(
  par_list_ref   = make_par_list(epsilon = 0.5, prep = 1, trep = 0),
  Rnaught        = Rnaught,
  alpha          = alpha,
  with_dem_stoch = FALSE,
  nsim           = nsim,
  loess_par      = qstar
)
save(rrmse_esxxxx, file = "RData/s2dgbeta_esxxxx.RData")
```

Second, we consider simulations with environmental stochasticity ($\epsilon = 0.5$), with demographic stochasticity, and without observation error ($p_{\text{rep}} = 1$, $t_{\text{rep}} = 0$).

```
rrmse_esdsxx <- test_s2dgbeta(
  par_list_ref   = make_par_list(epsilon = 0.5, prep = 1, trep = 0),
  Rnaught        = Rnaught,
  alpha          = alpha,
  with_dem_stoch = TRUE,
  nsim           = nsim,
  loess_par      = qstar
)
save(rrmse_esdsxx, file = "RData/s2dgbeta_esdsxx.RData")
```

Third, we consider simulations with environmental stochasticity ($\epsilon = 0.5$), with demographic stochasticity, and with observation error ($p_{\text{rep}} = 0.25$, $t_{\text{rep}} = 2$ weeks).

```
rrmse_esdsoe <- test_s2dgbeta(
  par_list_ref   = make_par_list(epsilon = 0.5, prep = 0.25, trep = 2),
  Rnaught        = Rnaught,
  alpha          = alpha,
  with_dem_stoch = TRUE,
```

```
  nsim            = nsim,
  loess_par       = qstar
)
save(rrmse_esdsoe, file = "RData/s2dgbeta_esdsoe.RData")
```

We apply `get_rrmse_50pct()` to the output of `test_s2dgbeta()` in order to compute the median RRMSE in each set of `nsim` estimates of $\beta(t)$.

```
get_rrmse_50pct <- function(rrmse, method) {
  apply(rrmse[, , , method], c(1, 2), quantile, probs = 0.5)
}
```

`get_rrmse_50pct()` takes as arguments `rrmse` (any of the three arrays defined earlier) and `method` (`"S"` or `"SI"`). It returns an array whose `[i, j]`th entry is the median RRMSE for the (i, j)th $(\mathcal{R}_0, \alpha)$ grid point.

Fig 5 displays heatmaps of median RRMSE obtained from the output of `get_rrmse_50pct()`. There is one heatmap for each choice of the arguments `rrmse` and `method` (3×2 heatmaps in total). Navy fill indicates median RRMSE less than the minimum shown on the colour scale.

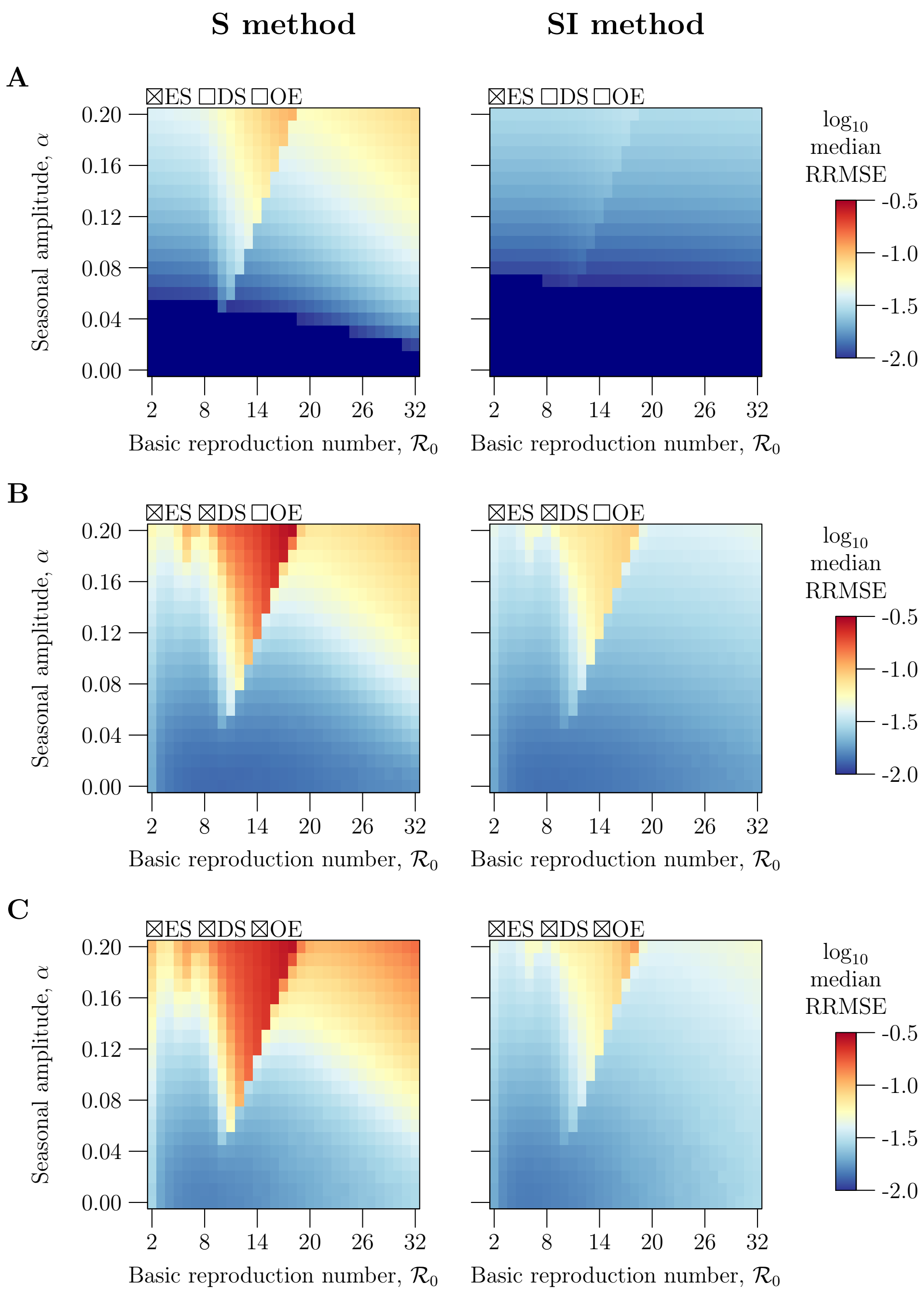

Fig 5. Sensitivity of $\beta(t)$ estimation error to the mean $\langle\beta\rangle$ and amplitude α of seasonal forcing. The $\langle\beta\rangle$ axis has been scaled to measure the basic reproduction number $\mathcal{R}_0$ (Eq (4)).

S5.2 Sensitivity to S_0, I_0, ν_c, μ_c, t_{gen}, and p_{rep}

Fig 6 describes how $\beta(t)$ estimation error varies as a function of data-generating parameters other than $\langle\beta\rangle$ and α: the initial states S_0 and I_0, vital rates ν_c and μ_c, mean generation interval t_{gen}, and case reporting probability p_{rep}.

To reproduce Fig 6, we explore lines in the data-generating parameter space by assigning all parameters their reference value in (9), except a focal parameter (one of S_0, I_0, ν_c, μ_c, t_{gen}, and p_{rep}), which we assign each of 41 values logarithmically spaced between $\frac{1}{4}$ and 4 times its reference value. Using each of these 5×41 or 6×41 parametrizations (we fix $p_{rep} = 1$ when we desire simulations without observation error), we simulate 1000 reported incidence time series.

```
scale_factors <- 2^seq(-2, 2, length.out = 41)
nsim <- 1000
```

We estimate $\beta(t)$ from each simulated reported incidence time series, without input error, fit a loess curve $\beta_{loess}(t; q)$ to the raw estimate β_k, and record the RRMSE in $\beta_{loess}(t_k; q)$. For comparison, this is done using both the S and SI methods. We fix $q = q^*$ (Eq (7)) independently of the β_k time series being smoothed. (See §S5.3 for discussion of the consequences of using fixed q in this analysis.)

This algorithm is implemented in our function `test_s2dgpars()`. ("sensitivity to data-generating parameters"). Its arguments are identical to those of `test_s2dgpars()`, except, instead of `Rnaught` and `alpha`, `test_s2dgpars()` has arguments

- `pars_to_vary`, a character vector listing the data-generating parameters to be treated as a focal parameter;
- `scale_factors`, a numeric vector listing the factors by which the reference value of each focal parameter is scaled to obtain the grid of values considered for that parameter.

`test_s2dgpars()` returns a 4-dimensional array, whose `[i, j, k, m]`th entry is the RRMSE in an estimate of $\beta(t)$ (S method if `m = 1`, SI method if `m = 2`) from simulation k of `nsim` in which `pars_to_vary[j]` is assigned its reference value times `scale_factors[i]`.

First, we consider simulations with environmental stochasticity ($\epsilon = 0.5$), without demographic stochasticity, and without observation error ($p_{rep} = 1$, $t_{rep} = 0$ weeks).

```
rrmse_esxxxx <- test_s2dgpars(
  pars_to_vary   = c("S0", "I0", "nu", "mu", "tgen"),
  par_list_ref   = make_par_list(epsilon = 0.5, prep = 1, trep = 0),
  scale_factors  = scale_factors,
```

```
  with_dem_stoch = FALSE,
  nsim           = nsim,
  loess_par      = qstar
)
save(rrmse_esxxxx, file = "RData/s2dgpars_esxxxx.RData")
```

Second, we consider simulations with environmental stochasticity ($\epsilon = 0.5$), with demographic stochasticity, and without observation error ($p_{\mathrm{rep}} = 1$, $t_{\mathrm{rep}} = 0$ weeks).

```
rrmse_esdsxx <- test_s2dgpars(
  pars_to_vary   = c("S0", "I0", "nu", "mu", "tgen"),
  par_list_ref   = make_par_list(epsilon = 0.5, prep = 1, trep = 0),
  scale_factors  = scale_factors,
  with_dem_stoch = TRUE,
  nsim           = nsim,
  loess_par      = qstar
)
save(rrmse_esdsxx, file = "RData/s2dgpars_esdsxx.RData")
```

Third, we consider simulations with environmental stochasticity ($\epsilon = 0.5$), with demographic stochasticity, and with observation error ($p_{\mathrm{rep}} = 0.25$, $t_{\mathrm{rep}} = 2$ weeks).

```
rrmse_esdsoe <- test_s2dgpars(
  pars_to_vary   = c("S0", "I0", "nu", "mu", "tgen", "prep"),
  par_list_ref   = make_par_list(prep = 0.25, trep = 2),
  scale_factors  = scale_factors,
  with_dem_stoch = TRUE,
  nsim           = nsim,
  loess_par      = qstar
)
save(rrmse_esdsoe, file = "RData/s2dgpars_esdsoe.RData")
```

In this third analysis, when S_0, I_0, ν_{c}, μ_{c}, or t_{gen} is varied, p_{rep} is fixed and assigned its reference value, 0.25. When p_{rep} itself is varied, we consider for p_{rep} each value in the vector `scale_factors * 0.25`.

As with `test_s2dgbeta()`, we apply `get_rrmse_50pct()` to the output of `test_s2dgpars()` in order to compute the median RRMSE in each set of `nsim` estimates of $\beta(t)$.

Fig 6 displays the output of `get_rrmse_50pct()`, plotting median RRMSE as a function of each data-generating parameter. To be precise, the horizontal axis measures the ratio of the data-generating and reference values of the focal parameter, which ranges from

$\frac{1}{4}$ to 4 regardless of the focal parameter. This allows results for different parameters to be compared in one panel.

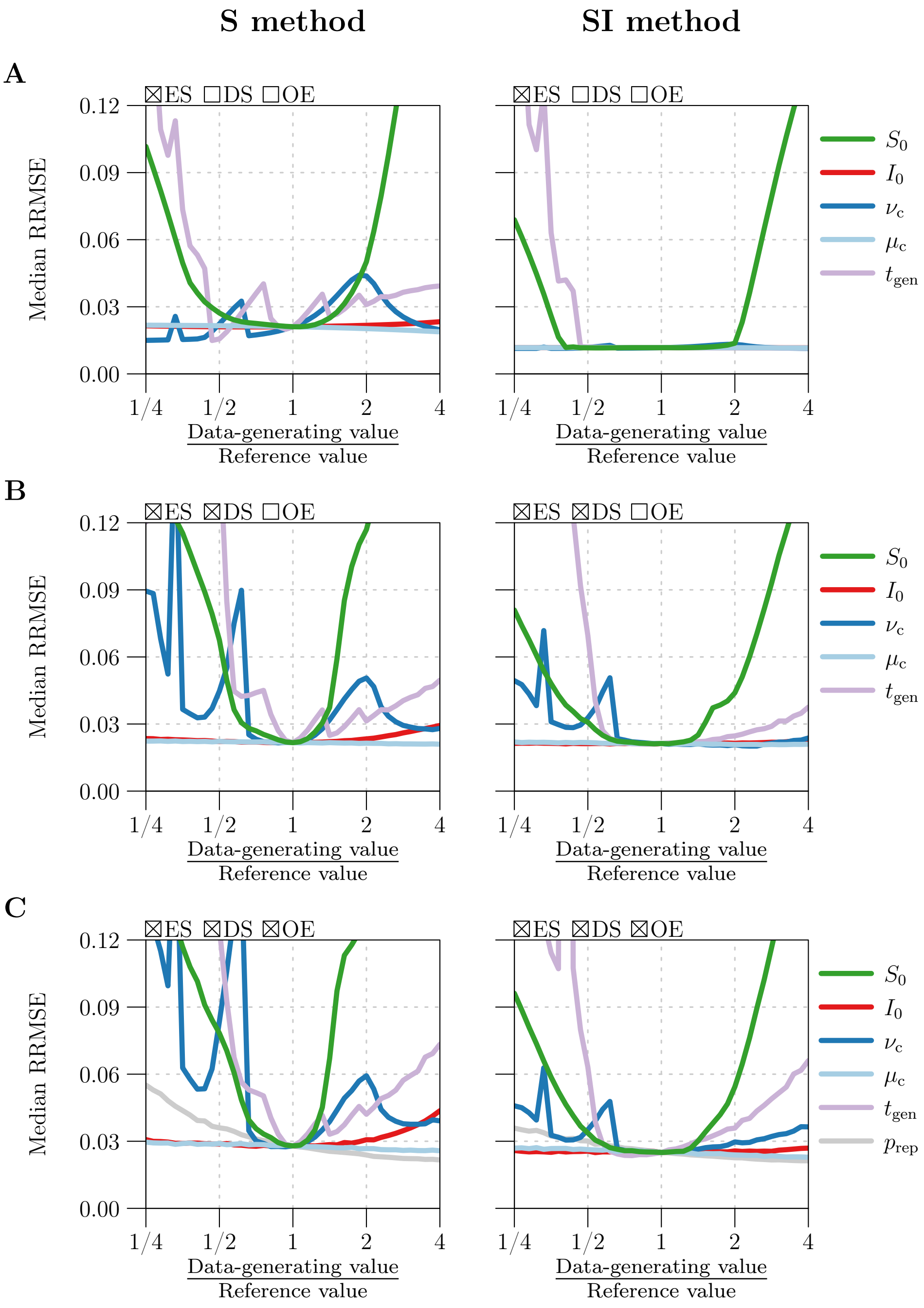

Fig 6. Sensitivity of $\beta(t)$ estimation error to data-generating parameters other than $\langle\beta\rangle$ and α.

S5.3 A note on smoothing

To generate Figs 5 and 6, we fixed $q = q^*$ (Eq (7)) when fitting loess curves $\beta_{\text{loess}}(t; q)$ to raw transmission rate estimates β_k. For a given β_k time series, this setting may not have been optimal ($q^* \neq q_{\text{opt}}$), meaning that the RRMSE calculated for $\beta_{\text{loess}}(t_k; q)$ was greater with $q = q^*$ than it would have been had we found q_{opt} and used $q = q_{\text{opt}}$.

This is potentially problematic, because sensitivity to data-generating parameters is mediated by propagation of noise from the simulated reported incidence data to β_k. We may have observed less sensitivity to a parameter (for example, t_{gen} in Fig 6) had we smoothed more when there was extreme noise in β_k (*i.e.*, had we set $q = q_{\text{opt}}$ when $q_{\text{opt}} > q^*$). We did not do this, because finding q_{opt} for each of the 5×10^6 time series considered by Figs 5 and 6 would have increased the total computation time by a factor of 100. Hence Figs 5 and 6 may overestimate the sensitivity of $\beta(t)$ estimation error to data-generating parameters.

Nevertheless, we expect the quantitative effect of choosing q^* over q_{opt} to be relatively small. Consider the graph corresponding to t_{gen} in the right panel of Fig 6C, which displays median RRMSE close to $(0.12, 0.03, 0.045)$ when t_{gen} is $(2^{-1.5}, 1, 2^{1.5}) \cdot 13$ days, respectively (13 days being the reference value). For these values of t_{gen}, it is instructive to compare (i) simulated reported incidence time series C_k, (ii) raw transmission rate estimates β_k from C_k, and (iii) the corresponding loess estimates $\beta_{\text{loess}}(t; q^*)$ and $\beta_{\text{loess}}(t; q_{\text{opt}})$.

S5.3.1 C_k and β_k for $t_{\text{gen}} = (2^{-1.5}, 1, 2^{1.5}) \cdot 13$ days

First, we simulate a reported incidence time series C_k using each of $t_{\text{gen}} = (2^{-1.5}, 1, 2^{1.5}) \cdot 13$ days. All three simulations account for environmental stochasticity ($\epsilon = 0.5$), demographic stochasticity, and observation error ($p_{\text{rep}} = 0.25$).

```r
## List of reference parameter values
par_list_ref <- make_par_list(epsilon = 0.5, prep = 0.25)

## List of lists of data-generating parameter values
par_list <- mapply(make_par_list,
  tgen      = 2^c(-1.5, 0, 1.5) * par_list_ref$tgen,
  beta_mean = par_list_ref$beta_mean,
  Rnaught   = NA,
  epsilon   = 0.5,
  prep      = 0.25,
  SIMPLIFY  = FALSE
)

## List of data frames containing time series data
df <- mapply(make_data,
```

```
  par_list = par_list,
  n = 20 * 365 / 7,
  with_dem_stoch = TRUE,
  seed = c(1836, 6183, 3618),
  SIMPLIFY = FALSE
)
```

Plotting each C_k time series yields the following result. Note that we are simply plotting `df[[i]]$C` for $i = 1, 2, 3$.

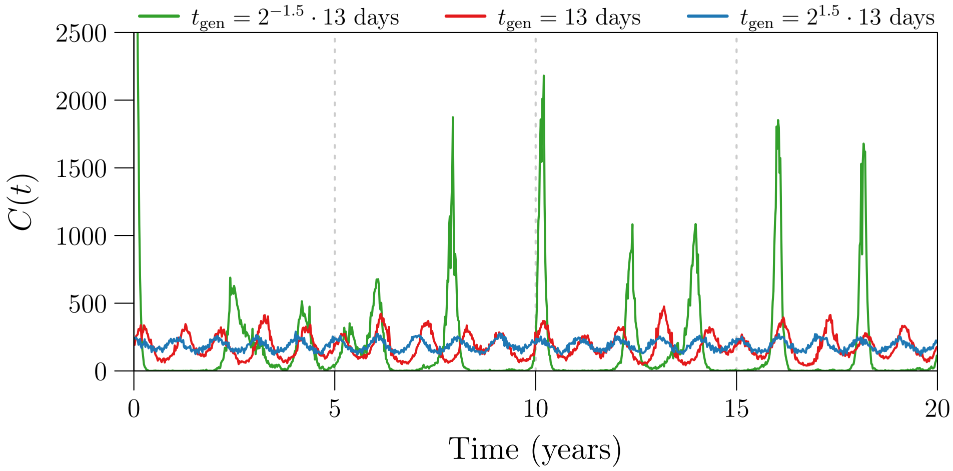

We see that a period-doubling bifurcation occurs between $t_{gen} = 13$ days and $t_{gen} = 2^{-1.5} \cdot 13$ days, with C_k attaining a much smaller minimum in the time series with a 2-year cycle (generated by $t_{gen} = 2^{-1.5} \cdot 13$ days).

Due to much closer approaches to zero by incidence and prevalence with $t_{gen} = 2^{-1.5} \cdot 13$ days, noise in C_k is amplified to a much greater extent in the raw transmission rate estimate β_k. We show this by applying the SI method without input error to estimate the underlying, seasonally forced transmission rate $\beta(t)$ (Eq (5))—which was the same across simulations—from each C_k time series.

```
## List of data frames containing estimation output
df_est <- mapply(estimate_beta_SI,
  df = df,
  par_list = par_list,
  SIMPLIFY = FALSE
)
```

Plotting β_k shows that, indeed, propagation of noise from C_k to β_k is much more severe when $t_{gen} = 2^{-1.5} \cdot 13$ days. Note that we are simply plotting `df_est[[i]]$beta`, scaled by `with(par_list, 1/beta_mean)`, for $i = 1, 2, 3$.

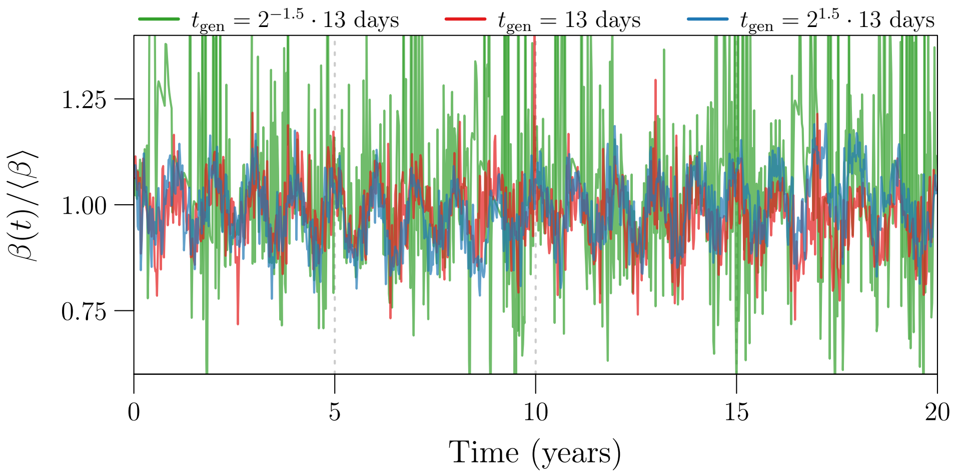

We calculate the RRMSE in each of these estimates as follows.

```
rrmse_raw <- mapply(
  function(x, y) compute_rrmse(x$beta, y$beta),
  x = df,
  y = df_est
)
rrmse_raw

## [1] 0.26917391 0.06715921 0.05893578
```

S5.3.2 $\beta_{\text{loess}}(t; q^*)$ and $\beta_{\text{loess}}(t; q_{\text{opt}})$ for $t_{\text{gen}} = (2^{-1.5}, 1, 2^{1.5}) \cdot 13$ days

All three of these estimates of $\beta(t)$ are greatly improved with loess smoothing. First, we consider smoothing all three β_k time series with $q = q^*$.

```
## List of `loess` objects encoding the fitted loess curves
loess_fit <- lapply(df_est,
  function(x) {
    loess(
      formula   = beta ~ t,
      data      = x,
      span      = qstar["SI"] / nrow(x),
      degree    = 2,
      na.action = "na.exclude",
      control   = loess.control(surface = "direct")
    )
  }
)
```

Plotting these loess estimates $\beta_{\text{loess}}(t; q^*)$ yields the following result. Note that we are plotting (with a scaling) `predict(loess_fit[[i]])` for $i = 1, 2, 3$.

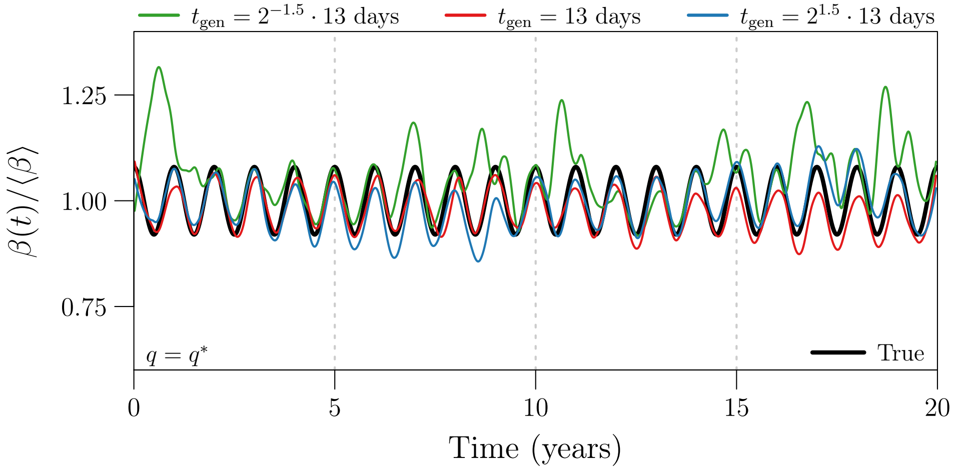

We calculate the RRMSE in each of these estimates as follows.

```
rrmse_loess_qstar <- mapply(
  function(x, y) compute_rrmse(x$beta, predict(y)),
  x = df,
  y = loess_fit
)
rrmse_loess_qstar

## [1] 0.10502016 0.03356297 0.03478337
```

Next, we consider smoothing each β_k time series with $q = q_{\text{opt}}$. Of course, we must first find q_{opt}.

```
q <- 10:150

## Array of values of RRMSE. Entry `[i, j]` contains the RRMSE
## in the loess estimate obtained from `df_est[[j]]$beta` using
## `q[i]` for the loess smoothing parameter.
rrmse_loess <- mapply(
  function(x, y) {
    sapply(q, function(z) {
      loess_fit <- loess(
        formula   = beta ~ t,
        data      = y,
        span      = z / nrow(y),
        degree    = 2,
```

```
        na.action = "na.exclude",
        control   = loess.control(
          surface    = "direct",
          statistics = "none"
        )
      )
      compute_rrmse(x$beta, predict(loess_fit))
    })
  },
  x = df, y = df_est, SIMPLIFY = TRUE
)
dim(rrmse_loess)

## [1] 141   3

## Optimal value for loess smoothing parameter is that
## which minimizes RRMSE
qopt <- apply(rrmse_loess, 2, function(x) q[which.min(x)])
qopt

## [1] 150  50  52

## List of `loess` objects encoding the fitted loess curves
loess_fit <- mapply(
  function(x, y) {
    loess(
      formula   = beta ~ t,
      data      = x,
      span      = y / nrow(x),
      degree    = 2,
      na.action = "na.exclude",
      control   = loess.control(surface = "direct")
    )
  },
  x = df_est, y = qopt, SIMPLIFY = FALSE
)
```

As expected, the β_k time series corresponding to $t_{\text{gen}} = 2^{-1.5} \cdot 13$ days requires the most smoothing (greatest q_{opt}). Plotting these *optimal* loess estimates $\beta_{\text{loess}}(t; q_{\text{opt}})$ yields the following result. Once again, we are plotting (with a scaling) `predict(loess_fit[[i]])` for $i = 1, 2, 3$.

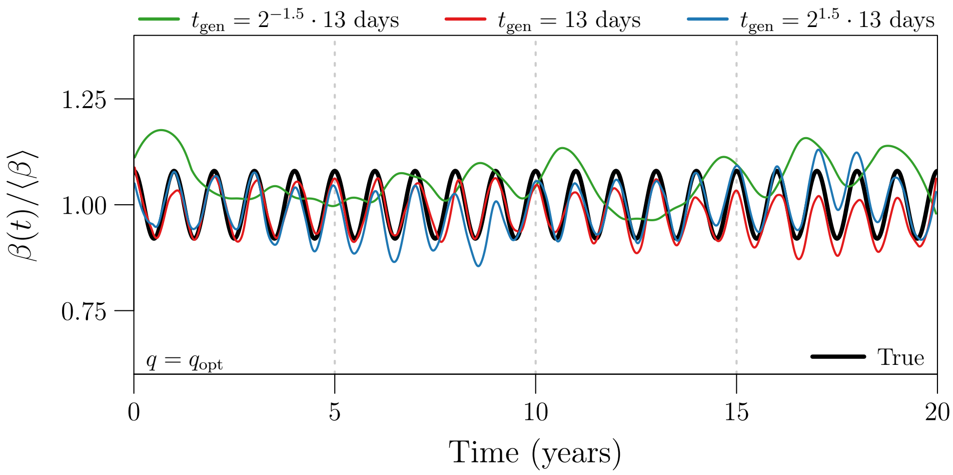

The RRMSE in each of these estimates is calculated as before.

```
rrmse_loess_qopt <- mapply(
  function(x, y) compute_rrmse(x$beta, predict(y)),
  x = df,
  y = loess_fit
)
rrmse_loess_qopt

## [1] 0.09848373 0.03346263 0.03475891
```

S5.3.3 Discussion

Comparing β_k, $\beta_{loess}(t; q^*)$, and $\beta_{loess}(t; q_{opt})$ for each value of t_{gen}, we find that when noise in β_k is severe (in this example, when $t_{gen} = 2^{-1.5} \cdot 13$ days), even an optimal degree of smoothing cannot recover the true $\beta(t)$ from the noise, due to underlying bias. No amount of variance reduction can correct the error due to bias. For this reason, smoothing β_k using q^* for the loess smoothing parameter q was never much worse than smoothing using the optimal value q_{opt}, even when $q^* \ll q_{opt}$ (as was the case with $t_{gen} = 2^{-1.5} \cdot 13$ days):

```
## Summary of results
data.frame(
  rrmse_raw,
  qstar = as.numeric(qstar["SI"]),
  rrmse_loess_qstar,
  qopt,
  rrmse_loess_qopt
)
```

```
##    rrmse_raw qstar rrmse_loess_qstar qopt rrmse_loess_qopt
## 1 0.26917391    53        0.10502016  150       0.09848373
## 2 0.06715921    53        0.03356297   50       0.03346263
## 3 0.05893578    53        0.03478337   52       0.03475891
```

This suggests that the decision to fix $q = q^*$ when generating Figs 5 and 6 does not greatly mischaracterize the effect of parameters like t_{gen} on $\beta(t)$ estimation error. Had we found q_{opt} for each raw estimate β_k, we would have calculated quantitatively similar RRMSE.

S6 Sensitivity to error in input parameters

Error in estimates of the seasonally forced transmission rate (Eq (5)) from simulated reported incidence data is also a function of the user-specified values of input parameters, given by

$$\boldsymbol{\theta}' = (S_0', I_0', \nu_c', \mu_c', t_{\text{gen}}', p_{\text{rep}}', t_{\text{rep}}') \,. \tag{12}$$

Input error arises when the user's input mischaracterizes the data-generating process. In our simulated data setting, this occurs when the specified value of an input parameter differs from the value used to simulate data (*e.g.*, when $S_0' \neq S_0$, and so on).

Fig 7A in the manuscript describes how $\beta(t)$ estimation error varies as a function of input error. To reproduce Fig 7A, we simulate 1000 reported incidence time series using the reference values in (9) for all data-generating parameters. From each reported incidence time series, we estimate the underlying $\beta(t)$ using the S and SI methods with different errors in the input. Specifically, we explore lines in the input parameter space by assigning all input parameters their true (data-generating) value, except a focal parameter (one of S_0, I_0, ν_c, μ_c, t_{gen}, p_{rep}, and t_{rep}), which we assign each of 41 values logarithmically spaced between $\frac{1}{4}$ and 4 times its true value. Hence, in total, we consider 7×41 parametrizations of the S and SI methods. We fit loess curves $\beta_{\text{loess}}(t; q)$ to each raw transmission rate estimate β_k generated in this process, fixing $q = q^*$ (Eq (7)), and record the RRMSE in $\beta_{\text{loess}}(t_k; q^*)$ (See §S6.1 for discussion of the consequences of using fixed q in this analysis.)

The above algorithm is implemented in our function `test_s2inpars()` ("sensitivity to input parameters"), which takes as arguments

- `par_list_ref`, a list containing values for all data-generating parameters, used in all simulations;
- `pars_to_vary`, a character vector listing the input parameters to be treated as a focal parameter;
- `scale_factors`, a numeric vector listing the factors by which the data-generating value of each focal parameter is scaled to obtain the grid of input values considered for that parameter;

- `with_dem_stoch`, a logical scalar indicating whether simulations should account for demographic stochasticity;
- `nsim`, the number of simulations to perform;
- `loess_par`, a numeric vector of length 2 specifying the value of the loess smoothing parameter q used when fitting loess curves to raw transmission rate estimates β_k. `loess_par[1]` is used with the S method. `loess_par[2]` is used with the SI method.

`test_s2inpars()` returns a 4-dimensional array, whose `[i, j, k, m]`th entry is the RRMSE in the estimate of $\beta(t)$ from simulation k of `nsim`, generated by assigning `pars_to_vary[j]` its true (data-generating) value times `scale_factors[i]` in the input to the S (`m = 1`) or SI (`m = 2`) method.

We reproduce Fig 7A starting with the following call to `test_s2inpars()`.

```
rrmse_esdsoe <- test_s2inpars(
  pars_to_vary   = c("S0", "I0", "nu", "mu", "tgen", "prep", "trep"),
  par_list_ref   = make_par_list(epsilon = 0.5, prep = 0.25, trep = 2),
  scale_factors  = 2^seq(-2, 2, length.out = 41),
  with_dem_stoch = TRUE,
  nsim           = 1000,
  loess_par      = qstar
)
save(rrmse_esdsoe, file = "RData/s2inpars_esdsoe.RData")
```

Fig 7A plots the median RRMSE obtained with each parametrization of the SI method. We retrieve medians from `rrmse_esdsoe` in the next code chunk. Note that some parametrizations cause the SI method to fail. For example, modest underestimation of ν_c by ν_c' or of p_{rep} by p_{rep}' causes S_k—the SI method estimate of $S(t_k)$—to become negative. When this happens, `test_s2inpars()` assigns RRMSE the value `NA`. Below, we calculate the median RRMSE only for those parametrizations that yield a full set of 1000 values of RRMSE (no `NA`s).

```
## Preallocate memory for median RRMSE:
## one value for each parametrization of the SI method
rrmse_50pct <- NA * rrmse_esdsoe[, , 1, "SI"]
dim(rrmse_50pct)

## [1] 41  7

## Define vector indexing parametrizations for which RRMSE
```

```r
## was never `NA`
ind_no_na <- which(
  apply(!is.na(rrmse_esdsoe[, , , "SI"]), c(1, 2), all)
)

## Calculate median RRMSE for the indexed parametrizations
rrmse_50pct[ind_no_na] <- sapply(ind_no_na, function(i) {
  ai <- arrayInd(i, dim(rrmse_50pct))
  quantile(rrmse_esdsoe[ai[1], ai[2] , , "SI"],
    probs = 0.5,
    na.rm = TRUE
  )
})
```

Fig 7B repeats the analysis from Fig 7A concerning mis-specification of S_0, except with initially erroneous estimates of S_0 corrected using peak-to-peak iteration (PTPI; see §S7 below for an actual illustration of this technique) before being passed to the S and SI methods. We generate results with PTPI by repeating the last call to `test_s2inpars()` with the additional argument `ptpi_iter = 25`, indicating that PTPI should be employed and stopped after 25 iterations. Since PTPI only affects results for S_0, we set `pars_to_vary = "S0"`.

```r
rrmse_esdsoe_ptpi <- test_s2inpars(
  pars_to_vary   = "S0",
  par_list_ref   = make_par_list(epsilon = 0.5, prep = 0.25, trep = 2),
  scale_factors  = 2^seq(-2, 2, length.out = 41),
  with_dem_stoch = TRUE,
  nsim           = 1000,
  loess_par      = qstar,
  ptpi_iter      = 25
)
save(rrmse_esdsoe_ptpi, file = "RData/s2inpars_esdsoe_ptpi.RData")
```

There are no issues with RRMSE being assigned `NA` in this analysis, so calculating median RRMSE is more straightforward.

```r
rrmse_ptpi_50pct <- apply(
  rrmse_esdsoe_ptpi[, "S0", , "SI"], 1, quantile,
  probs = 0.5,
  names = FALSE
)
```

We reproduce Fig 7 by plotting median RRMSE—saved in `rrmse_50pct` and `rrmse_ptpi_50pct`—as a function of the ratio of the specified value of the focal parameter to the true (data-generating) value.

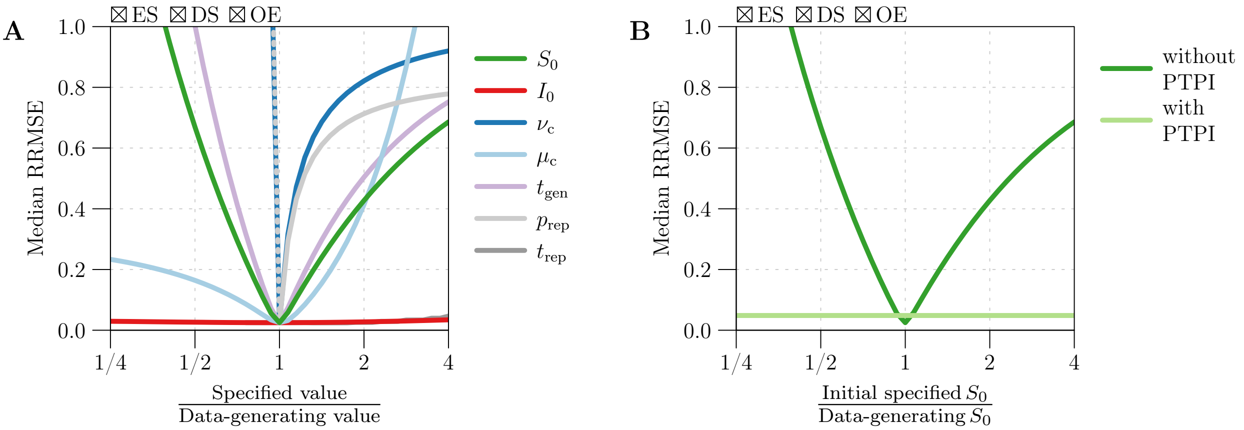

Fig 7. Sensitivity of $\beta(t)$ estimation error to the user-specified values of input parameters.

S6.1 A note on smoothing

The exact choice of the loess smoothing parameter q in this analysis is not critical, because error in the raw transmission rate estimate β_k is primarily due to bias caused by mis-specified input parameters. Moderate oversmoothing or undersmoothing of β_k has a negligible effect on RRMSE when β_k is extremely biased. (Fig 8 in the manuscript shows this clearly for the case of mis-specified S_0.) Hence the decision to fix $q = q^*$ (Eq (7)) as done here does not have a visible quantitative effect.

S7 Estimating S_0 via PTPI: Example

Fig 8 in the manuscript illustrates the use of peak-to-peak iteration (PTPI) to estimate the initial number of susceptible individuals $S_0 = S(t_0)$ from times series Z_k, B_k, and μ_k of (estimated) incidence, births, and the *per capita* natural mortality rate. The PTPI algorithm relies on the following:

(a) Periodicity of Z_k, meaning that Z_k displays recurrent epidemics.

(b) Accuracy of Z_k, B_k, and μ_k. Systematic errors in these time series bias the reconstruction of susceptibles by PTPI, and ultimately the estimate of S_0 to which the iterations converge. This makes sense, given that susceptible dynamics are the direct result of imbalance between susceptible recruitment through birth and susceptible depletion through infection and death.

To reproduce Fig 8, we simulate a reported incidence time series C_k with known underlying S_0 (to be estimated).

```
## List of data-generating parameter values
par_list <- make_par_list(epsilon = 0.5, prep = 0.25)

## Data frame containing time series data
df <- make_data(
  par_list       = par_list,
  n              = 20 * 365 / 7,
  with_dem_stoch = TRUE,
  seed           = 1350
)

## True value of `S0` to be estimated
df$S[1]

## [1] 54052
```

We estimate true incidence Z from reported incidence C_k as in the SI method:

$$Z(t_k) \approx Z_k = \tfrac{1}{p_{\text{rep}}} C_{k+r}\,, \quad r = \tfrac{[t_{\text{rep}}]_{\Delta t}}{\Delta t}\,,. \tag{13}$$

We do this using the true (data-generating) values of p_{rep} and t_{rep}, so that Z_k estimates Z without systematic error. (We consider this ideal case in order to demonstrate the validity of the PTPI algorithm. The sensitivity of the method to input error is not explored here explicitly, but is likely captured by the expressions for $\text{Err}(S_k, \xi \leftarrow \omega\xi)$ derived in §2.7.2 of the manuscript.)

```
## Time series of estimated incidence
Z <- estimate_beta_SI(df, par_list)$Z
```

We will pass this Z_k time series to the PTPI algorithm. The complete algorithm consists of a truncation step, described in Box 4 in the manuscript, followed by an iteration step, described in Box 5 in the manuscript. Below, we explain their implementation in R, generating Fig 8 in the process.

S7.1 Truncation step

The goal of the truncation step is to find the time t_a of the first peak in $\overline{Z}_k$ and the time t_b of the last peak occurring at the same phase of the cycle. Here, $\overline{Z}_k$ is a central moving average applied to the Z_k time series to remove unwanted noise. (Noise creates "peaks" in Z_k that we wish to ignore.)

Our function `get_peak_times()` automates the task of (i) applying a central moving average to any equally spaced, (roughly) periodic time series, then (ii) finding peak times. It takes as arguments

- `x`, a numeric vector specifying an equally spaced, (roughly) periodic time series;
- `period`, a numeric scalar specifying the period of `x` in units of the observation interval;
- `bw_mavg`, an integer scalar (a bandwidth) indicating that the central moving average applied to `x` should include `2 * bw_mavg + 1` points;
- `bw_peakid`, an integer scalar (a bandwidth) indicating that `x_mavg[i]` should be considered a peak if and only if `x_mavg[i]` $>$ `x_mavg[j]` for all j such that $0 < |i - j| <$ `bw_peakid`.

In the last item above, `x_mavg` is a vector of length `length(x)` containing the central moving average applied to `x`. `x_mavg[i]` is equal to `mean(x[(i-bw_mavg):(i+bw_mavg)])` for all i from `bw_mavg+1` to `length(x)-bw_mavg`, and equal to `NA` everywhere else (*i.e.*, at the the edges).

`get_peak_times()` returns a list containing `x_mavg` and two index vectors `all` and `phase`. `all` indexes all peaks in `x_mavg`, while `phase` indexes only those peaks in phase with the first peak (and is therefore a subset of `all`).

Before we construct a call to `get_peak_times()`, we must ascertain that our time series Z_k of estimated incidence is roughly periodic and determine the period. Plotting Z_k, it is clear that it is periodic with a 1-year cycle.

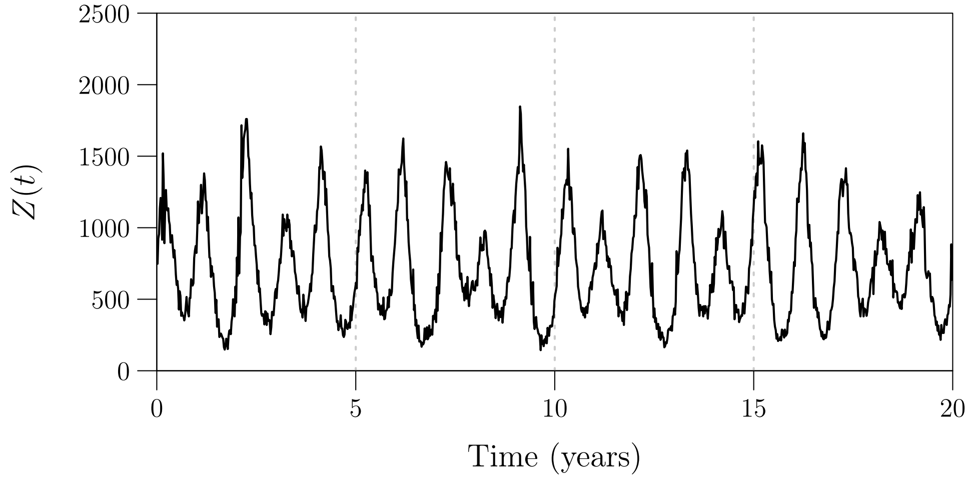

In general, it may be helpful to inspect the power spectrum of Z_k to determine the period, but we do not do this here.

We locate the peaks in $\overline{Z}_k$ with the following call to `get_peak_times()`.

```r
## List of index vectors for peaks in incidence time series
peaks <- get_peak_times(
  x          = Z,
  period     = with(par_list, (365 / 7) / dt_weeks),
  bw_mavg    = 6,
  bw_peakid  = 8
)

## All peaks
peaks$all[1:10]

##  [1]  61 118 171 218 274 322 383 429 478 539

## All peaks in phase with first
peaks$phase[1:10]

##  [1]  61 118 171 218 274 322 383 429 478 539
```

Above, we assigned `period` the value of 1 year in units of the observation interval. We chose bandwidths `bw_mavg = 6` and `bw_peakid = 8` using an simple tuning procedure. First, we chose the smallest value of `bw_mavg` that eliminated noise near peaks in Z_k. This was determined by visual inspection of the moving average $\overline{Z}_k$ (`peaks$x_mavg`). Next, we chose an arbitrary value of `bw_peakid` greater than 5 and less than half of `period` . This ensured that the definition of a peak was meaningful (a point greater than many of its nearest neighbours) and that peaks were not being compared against other peaks. The exact choice of `bw_peakid` tends not to be critical provided $\overline{Z}_k$ is smooth near the peaks.

Note that the two index vectors `all` and `phase` returned by `get_peak_times()` are identical. In this example, all peaks in $\overline{Z}_k$ are in phase, because the time between peaks is precisely the period (1 year). This is not true in general. For example, a 2-year cycle can have major and minor peaks that are out of phase. In this case, `all` would index both major and minor peaks, but `phase` would index either minor peaks or major peaks (but not both).

Plotting true incidence Z (`df$Z`), estimated incidence Z_k (`Z`), and the central moving average $\overline{Z}_k$ (`peaks$x_mavg`), as well as indicators of the times of peaks in $\overline{Z}_k$ in phase with the first peak (`peaks$phase`), we reproduce Fig 8A (see below). Fig 8A verifies that all of the peaks of interest were identified by `get_peak_times()` .

We conclude the truncation step of the PTPI algorithm by retrieving the index of the first peak in $\overline{Z}_k$ and the index of the last peak occurring at the same phase of the cycle.

```r
## Index of first peak
a <- with(peaks, phase[1])
## Index of last peak in phase with first peak
b <- with(peaks, phase[length(phase)])
```

The precise times t_a and t_b of these peaks are given by `df$t[c(a, b)]`.

S7.2 Iteration step

The goal of the iteration step is to use times series Z_k, B_k, and μ_k of (estimated) incidence, births, and the *per capita* natural mortality rate to iteratively update an initial estimate of $S_0 = S(t_0)$. This updating procedure depends on the result of the truncation step. The iteration step is implemented in our function `ptpi()`, which takes as arguments

- `df`, a data frame with columns `Z`, `B`, and `mu` specifying time series Z_k, B_k and μ_k of (estimated) incidence, births, and the *per capita* natural morality rate;
- `par_list`, a list with elements `hatN0`, `nu`, and `mu`, specifying a population size $\widehat{N}_0$ and constant vital rates ν_c and μ_c, which are used to create mock vital data in the event that `df` does not possess columns `B` or `mu` (`ptpi()` will set $B_k = \nu_c \widehat{N}_0 \Delta t$ and $\mu_k = \mu_c$ for all k);
- `a`, an integer scalar indicating the index of the first peak in `df$Z`;
- `b`, an integer scalar indicating the index of the last peak in `df$Z` in phase with the first peak;
- `initial_S0_est`, a numeric scalar indicating an initial estimate of S_0;
- `iter`, an integer scalar indicating the number of iterations to perform before stopping.

We carry out the iteration step with the following call to `ptpi()`. For this example, we suppose that our initial guess of S_0 is 4 times greater than its true value, and ask for our estimate to be updated 25 times. We provide the peak indices `a` and `b` obtained in the truncation step (see above). Finally, in the first argument, we specify our incidence time series and nothing else, and in the second argument, we specify the data-generating values of $\widehat{N}_0$, ν_c, and μ_c. This means that `ptpi()` will construct mock vital data without any systematic error and use it in conjunction with the supplied incidence time series.

```r
## List containing PTPI output
ptpi_out <- ptpi(
  df              = data.frame(Z),
```

```
  par_list       = par_list,
  a              = a,
  b              = b,
  initial_S0_est = df$S[1] * 4,
  iter           = 25
)
```

`ptpi()` returns a list with elements

- `S_mat`, a numeric matrix with `nrow(df)` rows and `iter+1` columns, containing the susceptible time series generated in each iteration;
- `S0`, a numeric vector of length `iter+1` listing the initial estimate of $S_0 = S(t_0)$ and the estimate obtained in each iteration (equivalent to `S_mat[1, ]`);
- `S0_final`, a numeric scalar indicating the final estimate of S_0 (equivalent to `S0[length(S0)]`);
- `SA`, a numeric vector of length `iter+1` listing the initial estimate of $S_a = S(t_a)$ (equal to the initial estimate of S_0) and the estimate obtained in each iteration (equivalent to `S_mat[a, ]`);
- `SA_final`, a numeric scalar indicating the final estimate of S_a (equivalent to `SA[length(SA)]`).

Examining `ptpi_out`, we find that the iterations converged to an accurate estimate of S_0.

```
## Ordered estimates of `S0`
ptpi_out$S0

##  [1] 216208.00 138745.04  94654.54  73216.89  62793.50  57725.45  55261.27
##  [8]  54063.13  53480.58  53197.33  53059.61  52992.64  52960.09  52944.26
## [15]  52936.56  52932.82  52931.00  52930.11  52929.68  52929.47  52929.37
## [22]  52929.32  52929.30  52929.29  52929.28  52929.28

## Relative error in final estimate of S0
(ptpi_out$S0_final - df$S[1]) / df$S[1]

## [1] -0.02077116
```

Fig 8B (see below) displays the `iter+1` susceptible time series S_k obtained in each iteration of PTPI. To reproduce Fig 8B, we plot the columns of `ptpi_out$S_mat`, scaled by `with(par_list, 1/N0)`.

```r
## Matrix with ordered susceptible time series as columns
ptpi_out$S_mat[1:10, 1:5]

##          [,1]     [,2]     [,3]     [,4]     [,5]
##  [1,] 216208 138745.0 94654.54 73216.89 62793.50
##  [2,]     NA 138657.8 94601.07 73179.87 62764.47
##  [3,]     NA 138394.6 94371.71 72966.93 62559.52
##  [4,]     NA 138099.7 94110.54 72722.18 62322.74
##  [5,]     NA 137625.1 93669.64 72297.67 61906.22
##  [6,]     NA 137078.8 93157.10 71801.53 61418.04
##  [7,]     NA 136581.0 92692.94 71353.74 60978.22
##  [8,]     NA 136327.4 92473.04 71150.21 60782.64
##  [9,]     NA 135470.3 91649.54 70343.06 59983.44
## [10,]     NA 134793.7 91006.60 69716.46 59364.78
```

Note that the first column contains `NA`, but only up to index `a` (not shown), where the first iteration starts.

Fig 8C (see below) displays the SI method estimate of the transmission rate corresponding to each estimate of S_0 listed in `ptpi_out$S0`. To reproduce Fig 8C, we pass each estimate of S_0 to `estimate_beta_SI()`, specifying the true (data-generating) value of every other input parameter. We fit a loess curve $\beta_{\text{loess}}(t; q^*)$ to each raw transmission rate estimate β_k, and record $\beta_{\text{loess}}(t_k; q^*)$ as a column in a matrix `beta_mat`. Finally we plot the columns of `beta_mat`, scaled by `with(par_list, 1/beta_mean)`.

```r
## Matrix with ordered transmission rate time series as columns
beta_mat <- sapply(ptpi_out$S0,
  function(x) {
    par_list_with_err <- within(par_list, S0 <- x)
    df_est <- estimate_beta_SI(df, par_list_with_err)
    loess_fit <- loess(
      formula   = beta ~ t,
      data      = df_est,
      span      = qstar["SI"] / nrow(df_est),
      degree    = 2,
      na.action = "na.exclude",
      control   = loess.control(surface = "direct")
    )
    predict(loess_fit)
  }
)
beta_mat[1:10, 1:5]
```

```
##               [,1]         [,2]         [,3]         [,4]         [,5]
##  [1,]           NA           NA           NA           NA           NA
##  [2,] 2.978043e-06 4.632868e-06 6.774538e-06 8.736727e-06 1.016727e-05
##  [3,] 2.925529e-06 4.557144e-06 6.675664e-06 8.623999e-06 1.004922e-05
##  [4,] 2.875193e-06 4.484526e-06 6.580771e-06 8.515711e-06 9.935726e-06
##  [5,] 2.827043e-06 4.415025e-06 6.489875e-06 8.411877e-06 9.826795e-06
##  [6,] 2.781082e-06 4.348645e-06 6.402980e-06 8.312501e-06 9.722428e-06
##  [7,] 2.737311e-06 4.285386e-06 6.320081e-06 8.217575e-06 9.622613e-06
##  [8,] 2.695731e-06 4.225250e-06 6.241182e-06 8.127098e-06 9.527348e-06
##  [9,] 2.656352e-06 4.168251e-06 6.166301e-06 8.041095e-06 9.436658e-06
## [10,] 2.619180e-06 4.114399e-06 6.095452e-06 7.959580e-06 9.350560e-06
```

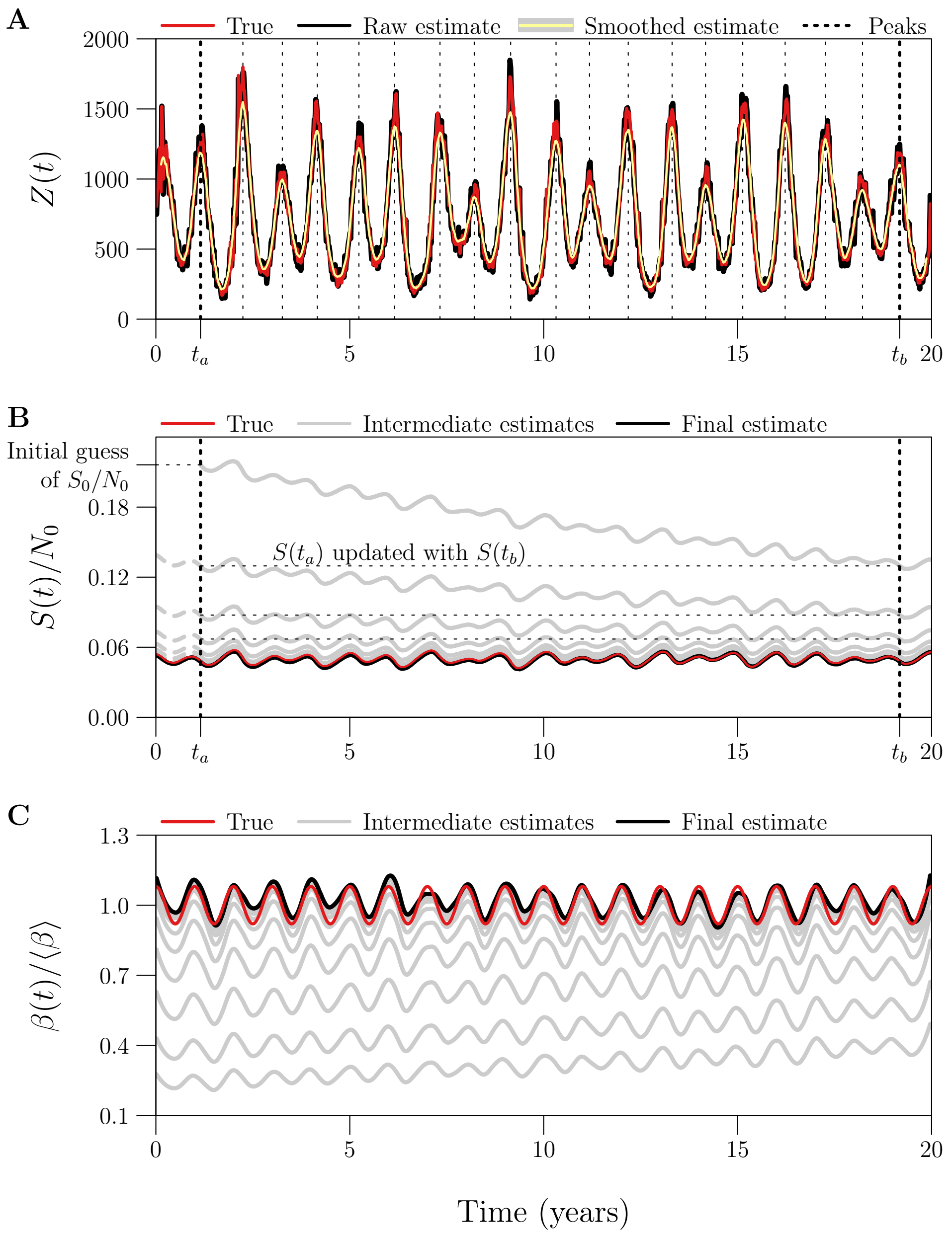

Fig 8. Example of $S(t)$ and $\beta(t)$ reconstruction with an overestimate of S_0 corrected by peak-to-peak iteration.

S8 Estimating S_0 via PTPI: Convergence

Fig 9 in the manuscript displays the result of applying PTPI (25 iterations) to estimate S_0 from 1000 realizations of a reported incidence time series, starting from each of two initial

guesses: $\frac{1}{4}$ and 4 times the true value. The aim of this analysis is to assess the bias and variance in the limiting estimate of S_0.

To reproduce Fig 9, we record the estimate obtained at each iteration (for each initial guess and simulation) in an array. The following code chunk preallocates space for this output and creates a list of parameter values to be used in simulations of reported incidence.

```
## Array with entry `[i, j, k]` equal to the `i`th estimate of `S0`
## generated from the `j`th initial guess and the `k`th simulated
## reported incidence time series
out <- array(NA, dim = c(26, 2, 1000))

## List of data-generating parameter values
par_list <- make_par_list(epsilon = 0.5, prep = 0.25)
```

The next code chunk fills in the `out` array with our desired output and saves it in `RData/ptpi_convergence.RData`.

```
for (k in 1:1000) {
  ## Data frame containing time series data
  df <- make_data(
    par_list       = par_list,
    n              = 20 * 365 / 7,
    with_dem_stoch = TRUE,
    seed = k
  )

  ## PTPI: truncation step
  Z <- estimate_beta_SI(df, par_list)$Z
  peaks <- get_peak_times(
    x         = Z,
    period    = with(par_list, (365 / 7) / dt_weeks),
    bw_mavg   = 6,
    bw_peakid = 8
  )

  ## PTPI: iteration step
  out[, , k] <- sapply(c(0.25, 4),
    function(x) {
      ptpi_out <- ptpi(
        df             = data.frame(Z),
        par_list       = par_list,
```

```
        a              = with(peaks, phase[1]),
        b              = with(peaks, phase[length(phase)]),
        initial_S0_est = with(par_list, S0 * x),
        iter           = 25
      )
      ptpi_out$S0 # all 26 estimates of `S0` in a vector
    }
  )
}
attr(out, "par_list") <- par_list
save(out, file = "RData/ptpi_convergence.RData")
```

We want the median and 5th and 95th percentiles of the estimate of S_0 obtained at each iteration (for each initial guess).

```
pct <- apply(out, c(1, 2), quantile,
  probs = c(0.05, 0.5, 0.95)
)
```

Plotting these as a functions of iteration, we reproduce Fig 9.

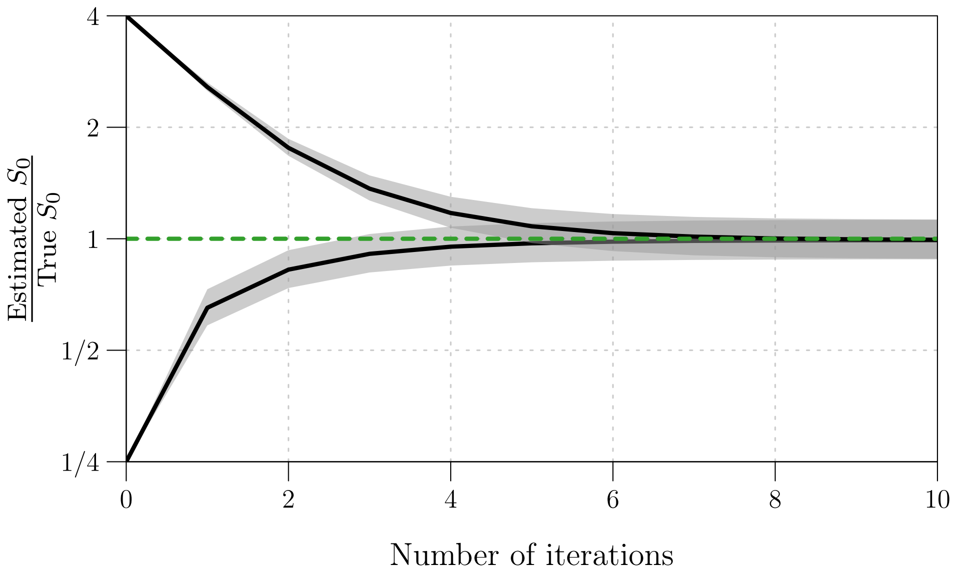

Fig 9. Convergence of estimates of S_0 obtained using peak-to-peak iteration.

In the manuscript, we report the median and 5th and 95th percentiles of the relative error in the estimate of S_0 obtained in the last iteration (for each initial guess). These are calculated as follows.

```r
with(par_list, (pct[, 26, ] - S0) / S0)

##              [,1]         [,2]
## 5%  -0.119343123 -0.119343062
## 50% -0.008528768 -0.008528708
## 95%  0.125379218  0.125379276
```

S9 Appendix: Choice of discretization in the SI method

The SI method modifies a method presented in [3] by deJonge. Here, we cast the SI method and deJonge's method as two possible algorithms from a set of nine, differing according to (i) how

$$\frac{\mathrm{d}S}{\mathrm{d}t} = \nu(t)\widehat{N}_0 - \beta(t)SI - \mu(t)S\,, \tag{14a}$$
$$\frac{\mathrm{d}I}{\mathrm{d}t} = \beta(t)SI - \gamma I - \mu(t)I \tag{14b}$$

are discretized (forward Euler, backward Euler, or trapezoidal method) in order to estimate susceptibles $S(t)$ and infecteds $I(t)$, and (ii) how

$$\frac{\mathrm{d}Q}{\mathrm{d}t} = \beta(t)SI \tag{15}$$

is discretized (forward Euler, backward Euler, or both) in order to estimate the transmission rate $\beta(t)$. ("Both" means that the two estimates of $\beta(t)$ obtained by forward and backward Euler are averaged to generate a final estimate.) DeJonge's method uses forward Euler throughout, whereas the SI method uses the trapezoidal method for Eqs (14) and both forward and backward Euler for Eq (15).

Here, we show that the SI method is more accurate than deJonge's method and the seven other algorithms. We further show that the SI method and deJonge's method are nearly unbiased (asymptotically) in the absence of input error.

S9.1 Nine discretization schemes

Our function `estimate_beta_SI()` takes a third argument `method`, which must be assigned a vector of length 2. `method[1]` has options `"forward"`, `"backward"`, and `"trapezoid"` (default), telling `estimate_beta_SI()` how to numerically integrate Eqs (14). `method[2]` has options `"forward"`, `"backward"`, and `"both"`, (default) telling `estimate_beta_SI()` how to numerically integrate Eq (15).

Eqs (16) below lay out the algorithm carried out by `estimate_beta_SI()`, conditional on `method[1]` and `method[2]`.

$$Z_k \leftarrow \frac{1}{p_{\text{rep}}} C_{k+r} \tag{16a}$$

$$S_k \leftarrow \begin{cases} (1 - \mu_{k-1}\Delta t)S_{k-1} + B_k - Z_k & \text{if } \texttt{method[1] = "forward"} \\ \frac{S_{k-1}+B_k-Z_k}{1+\mu_k \Delta t} & \text{if } \texttt{method[1] = "backward"} \\ \frac{(1-\frac{1}{2}\mu_{k-1}\Delta t)S_{k-1}+B_k-Z_k}{1+\frac{1}{2}\mu_k\Delta t} & \text{if } \texttt{method[1] = "trapezoid"} \end{cases} \tag{16b}$$

$$I_k \leftarrow \begin{cases} [1-(\gamma+\mu_{k-1})\Delta t]I_{k-1} + Z_k & \text{if } \texttt{method[1] = "forward"} \\ \frac{I_{k-1}+Z_k}{1+(\gamma+\mu_k)\Delta t} & \text{if } \texttt{method[1] = "backward"} \\ \frac{[1-\frac{1}{2}(\gamma+\mu_{k-1})\Delta t]I_{k-1}+Z_k}{1+\frac{1}{2}(\gamma+\mu_k)\Delta t} & \text{if } \texttt{method[1] = "trapezoid"} \end{cases} \tag{16c}$$

$$\beta_k \leftarrow \begin{cases} \frac{Z_{k+1}}{S_k I_k \Delta t} & \text{if } \texttt{method[2] = "forward"} \\ \frac{Z_k}{S_k I_k \Delta t} & \text{if } \texttt{method[2] = "backward"} \\ \frac{Z_k+Z_{k+1}}{2S_k I_k \Delta t} & \text{if } \texttt{method[2] = "both"} \end{cases} \tag{16d}$$

Hence the SI method corresponds to `method = c("trapezoid", "both")`, while deJonge's method corresponds to `method = c("forward", "forward")`.

S9.2 Comparison of RRMSE, bias, and variance

We will compare the nine algorithms described in Eqs (16) using two metrics. First, we consider performance as measured by the RRMSE in the raw transmission rate estimates β_k. Second, we consider bias in the average 1-year cycle, calculated from the linear interpolant of β_k as in §S3.

S9.2.1 RRMSE

We simulate 100 reported incidence time series C_k using each of 41 values for the case reporting probability p_{rep}, logarithmically spaced between 0.01 and 1. (Smaller values of p_{rep} generate noisier C_k, leading to noisier β_k.)

```
prep <- 10^seq(-2, 0, length.out = 41)
par_list <- make_par_list(epsilon = 0.5)
nsim <- 100
```

We estimate the underlying, seasonally forced transmission rate $\beta(t)$ (Eq (5)) from each simulated reported incidence time series using each algorithm described in Eqs (16), and record the RRMSE in each raw estimate β_k. We can preallocate space for this output.

```
method1_names <- c("forward", "backward", "trapezoid")
method2_names <- c("forward", "backward", "both")
out <- array(NA,
  dim      = c(length(prep), nsim, 3, 3),
  dimnames = list(NULL, NULL, method1_names, method2_names)
)
```

The next code chunk implements the steps in this routine, saving main results in the file `RData/euler.RData`. We can reuse simulations from §S4, which were saved in the directory `RData/loess/`.

```
for (i in seq_along(prep)) {

  ## Update `par_list` with current value of `prep`
  par_list$prep <- prep[i]

  ## Create a directory for this loop's `.RData`
  dirname <- paste0(
    "RData/loess/",
    ## log10 current value of `prep`
    "prep_log10v-", sprintf("%+05.0f", log(prep[i], 10) * 1000), "/"
  )
  if (!dir.exists(dirname)) {
    dir.create(dirname, recursive = TRUE)
  }

  for (j in seq_len(nsim)) {

    message(
      "`prep` value ", i, " of ", length(prep), ", ",
      "sim ", j, " of ", nsim
    )

    ## File name for simulation
    filename <- paste0(dirname, "sim", sprintf("%04.0f", j), ".RData")

    ## Simulate reported incidence data, if you haven't already
    if (file.exists(filename)) {
      load(filename)
    } else {
      df <- make_data(
        par_list       = par_list,
```

```
        n               = 20 * 365 / 7,
        with_dem_stoch = TRUE,
        seed           = j
      )
      save(df, file = filename)
    }

    for (m1 in method1_names) {
      for (m2 in method2_names) {

        ## Estimate the seasonally forced transmission rate
        ## from reported incidence
        df_est <- estimate_beta_SI(df, par_list, method = c(m1, m2))

        ## Record the error
        out[i, j, m1, m2] <- compute_rrmse(df$beta, df_est$beta)

      }
    }

  }

}


attr(out, "arg_list") <- list(
  prep      = prep,
  par_list  = par_list
)
save(out, file = "RData/euler.RData")
```

We desire the median and 5th and 95th percentiles of RRMSE for each value of p_{rep}, for each of the nine algorithms used to estimate $\beta(t)$.

```
pct <- apply(out, c(1, 3, 4), quantile, probs = c(0.05, 0.5, 0.95))
```

Plotting these as functions of p_{rep}, and stratifying the results by `method[1]` (panel title) and `method[2]` (legend label) yields the following figure.

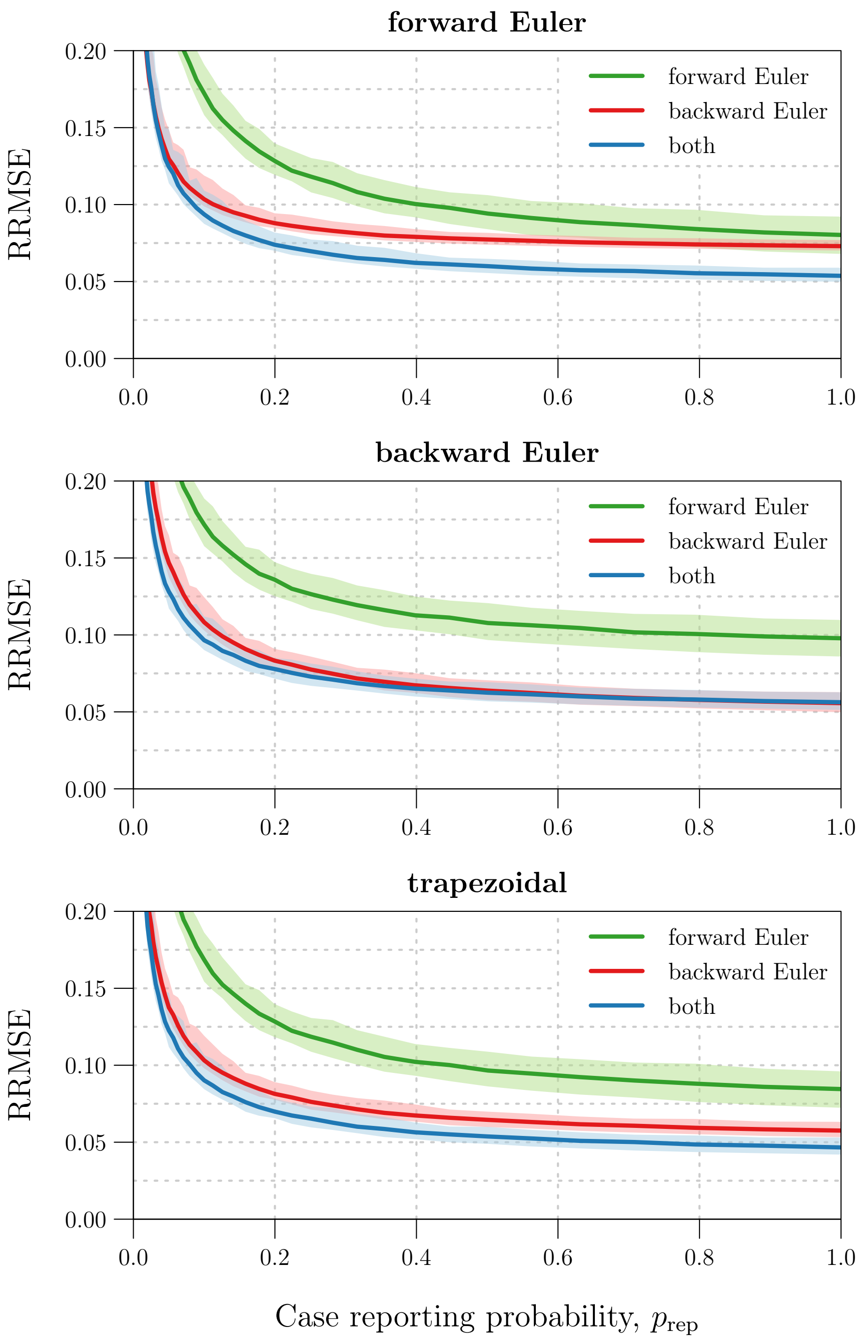

Fig 10. Performance of the nine discretization schemes, as measured by RRMSE in the raw transmission rate estimate β_k. Panel titles specify the discretization of Eqs (14). Legend labels specify the discretization of Eq (15).

For every choice of `method[1]` (panel title), the best choice of `method[2]` (legend label) was typically `"both"`. On the other hand, for a given choice of `method[2]`, the

best choice of `method[1]` (by a small margin) was typically the one that avoided mismatch with `method[2]`. That is, when forward and backward Euler were used to discretize Eq (15), RRMSE was typically smallest when forward and backward Euler, respectively, were used to discretize Eqs (14). Similarly, when both forward and backward Euler were used to discretize Eq (15), RRMSE was typically smallest when the trapezoidal method was used to discretize Eqs (14). This combination, with `method = c("trapezoid", "both")`, gave the best performance overall.

S9.2.2 Bias and variance

In §S3, we looked at bias and variance in the 1-year cycles embedded in raw transmission rate estimates β_k spanning 1000 years. There, we compared the S and SI methods. Here, we compare the nine algorithms described in Eqs (16).

We simulate 1000 years of weekly observations of reported incidence, including in the simulation environmental noise in transmission ($\epsilon = 0.5$), demographic stochasticity, and random under-reporting of cases ($p_{\text{rep}} = 0.25$).

```
par_list <- make_par_list(epsilon = 0.5, prep = 0.25)
df <- make_data(
  par_list       = par_list,
  n              = 1000 * 365 / 7 + 1,
  with_dem_stoch = TRUE,
  seed           = 1352
)
```

We estimate the seasonally forced $\beta(t)$ using all nine discretization schemes, without input error.

```
df_est <- mapply(
  function(x) {
    mapply(
      function(y) {
        estimate_beta_SI(df, par_list, method = c(x, y))
      },
      y = method2_names, SIMPLIFY = FALSE
    )
  },
  x = method1_names, SIMPLIFY = FALSE
)
```

We linearly interpolate each raw time series estimate β_k.

```r
fits <- lapply(df_est, function(x) {
  lapply(x, function(y) {
    approxfun(y$t, y$beta, method = "linear", rule = 1)
  })
})
```

As in §S3, we define the initial observation time `t0`, period `period`, and number of cycles `m`, then use `get_phase_average()` to calculate the average 1-year cycle in the linear interpolants.

```r
## First and last time points, retrievable
## from any data frame in the list
t0 <- df_est[[1]][[1]]$t[1]
tn <- df_est[[1]][[1]]$t[nrow(df_est[[1]][[1]])]

## 1-year period in units of the observation interval
period <- with(par_list, (365 / 7) / dt_weeks)

## Number of cycles
m <- floor((tn - t0) / period)

get_phase_average <- function(s, f) {
  x <- f(t0 + (s %% period) + (0:(m-1)) * period)
  mean(x, na.rm = TRUE)
}

s_grid <- seq(0, period, length.out = 150)
average_one_year <- lapply(fits, function(x) {
  data.frame(
    s_grid,
    lapply(x, function(f) sapply(s_grid, get_phase_average, f = f))
  )
})
```

We plot the 1000 individual cycles and their average on the same 1-year axis, yielding a 9-panel plot.

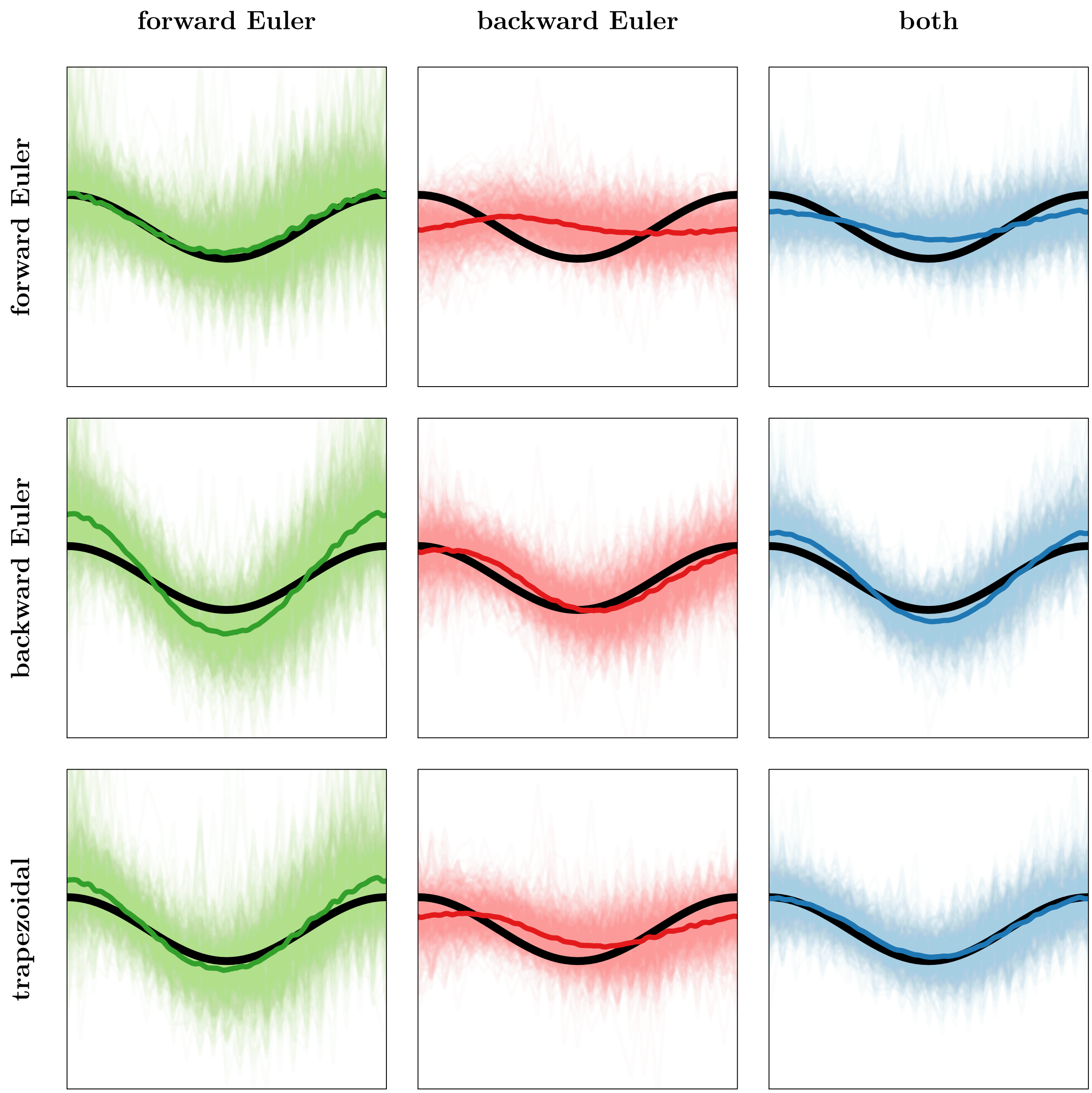

Fig 11. Bias and variance incurred by the nine discretization schemes. Row names specify the discretization of Eqs (14). Column names specify the discretization of Eq (15).

Following the pattern of Fig 10, Fig 11 shows that mismatch between `method[1]` and `method[2]` is detrimental: in the off-diagonal panels, the average 1-year cycle fails to capture the correct seasonal amplitude. In addition, use of backward Euler to discretize Eqs (14) appears ill-advised: in the panels from the second row, the average 1-year cycle lags the true cycle. Finally, it is apparent that the SI method (bottom right panel) and deJonge's method (top left panel) are both nearly unbiased.

All methods appear prone to propagating noise from reported incidence (due to process and observation error) to β_k. However, the SI method and deJonge's method stand out as being the least and most susceptible, respectively, to propagation of spurious noise. This likely accounts for the difference in their performance shown in Fig 10.

References

1. R Core Team. R: A language and environment for statistical computing; 2020. Available from: https://www.R-project.org.

2. Hart JD. Automated kernel smoothing of dependent data by using time series cross-validation. Journal of the Royal Statistical Society B (Statistical Methodology). 1994;56(3):529–542.

3. deJonge MS. Fast estimation of time-varying transmission rates. Hamilton, Ontario, Canada: McMaster University; 2014. Available from: https://macsphere.mcmaster.ca/handle/11375/14230.
