# Supplementary material for: Fast estimation of time-varying infectious disease transmission rates
Source: PLoS Comput Biol. 2020 Sep 21;16(9):e1008124. doi: 10.1371/journal.pcbi.1008124 (PMC7549798; doi:10.1371/journal.pcbi.1008124)
Supplement: S1 File — A .zip archive containing all of the source files needed to compile S1 Text. (ZIP) [file pcbi.1008124.s002.zip › S1_File/README.html]

Compiling S1\_Text.Rnw


# Compiling `S1_Text.Rnw`

## Dependencies

Compilation requires installation of R (see https://www.r-project.org/) and a LaTeX distribution (see https://www.latex-project.org/). It also depends on several R packages, including our package **fastbeta** located on GitHub. These can be installed in R as follows:

```
package_list <- c("remotes", "knitr", "tikzDevice",
                  "colorRamps", "RColorBrewer", "scales")
for (package_name in package_list) {
  if (!require(package_name, character.only = TRUE)) {
    install.packages(package_name)
  }
}
remotes::install_github("davidearn/fastbeta", ref = "plos")
```

## Compilation

### From RStudio

Open `S1_Text.Rnw` with RStudio, so that the working directory contains the file. Alternatively, just run RStudio and set the working directory with `setwd()`.

Compile `S1_Text.Rnw` by running `knitr::knit2pdf("S1_Text.Rnw")` in the console. Compilation should finish in less than 20 minutes, but the exact time is machine-dependent.

The compilation will generate `S1_Text.pdf` in the working directory. Open it with a PDF viewer of your choice.

### From the command line

Navigate to the directory containing `S1_Text.Rnw`, then run:

```
R --no-echo -e 'knitr::knit("S1_Text.Rnw")'
pdflatex S1_Text.tex
pdflatex S1_Text.tex
#evince S1_Text.pdf # Linux
#open S1_Text.pdf # macOS
```
